# Metallic Barium: A Versatile and Efficient Hydrogenation Catalyst

**DOI:** 10.1002/anie.202014326

**Published:** 2020-12-21

**Authors:** Philipp Stegner, Christian Färber, Ulrich Zenneck, Christian Knüpfer, Jonathan Eyselein, Michael Wiesinger, Sjoerd Harder

**Affiliations:** ^1^ Chair of Inorganic and Organometallic Chemistry Universität Erlangen-Nürnberg Egerlandstrasse 1 91058 Erlangen Germany

**Keywords:** alkaline earth metals, barium, hydride, hydrogenation catalysis, metal activation

## Abstract

Ba metal was activated by evaporation and cocondensation with heptane. This black powder is a highly active hydrogenation catalyst for the reduction of a variety of unactivated (non‐conjugated) mono‐, di‐ and tri‐substituted alkenes, tetraphenylethylene, benzene, a number of polycyclic aromatic hydrocarbons, aldimines, ketimines and various pyridines. The performance of metallic Ba in hydrogenation catalysis tops that of the hitherto most active molecular group 2 metal catalysts. Depending on the substrate, two different catalytic cycles are proposed. A: a classical metal hydride cycle and B: the Ba metal cycle. The latter is proposed for substrates that are easily reduced by Ba^0^, that is, conjugated alkenes, alkynes, annulated rings, imines and pyridines. In addition, a mechanism in which Ba^0^ and BaH_2_ are both essential is discussed. DFT calculations on benzene hydrogenation with a simple model system (Ba/BaH_2_) confirm that the presence of metallic Ba has an accelerating effect.

## Graphical abstract

Just a pinch of Ba metal: Efficient hydrogenation of a wide range of substrates is achieved with metallic barium previously activated by metal vapor synthesis. A mechanism in which Ba^0^ and BaH_2_ are both essential is discussed.

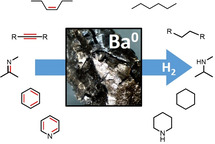

## Introduction

Two major milestones stand at the cradle of heterogeneous metal catalysis.^[1]^ Döbereiner observed in 1823 that hydrogen spontaneously burns in air upon contact with finely divided platinum. In 1912 Sabatier was awarded the Nobel prize in chemistry for the development of nickel catalysed alkene hydrogenation. The scientific basis for both discoveries is bond activation and cleavage at the surface of a metal.

It is hardly known that, following these groundbreaking studies on the catalytic activity of the late transition metals, catalytic ethylene hydrogenation with the much less noble alkaline earth (Ae) metal Ca has been described as early as 1925.^[2]^ Schmidt observed that the larger group 2 metal Ba is an even more reactive hydrogenation catalyst.^[3]^ In his extensive review on heterogeneous catalytic reactions, discrimination between two groups of metal catalysts is made. The first are the more noble transition metals with high ionization potentials that form solid solutions of hydrogen. The second class is represented by electropositive metals that form saline hydrides. It was proposed that the catalytic activity of the latter metals is strongly related to metal hydride formation.^[3]^ Indeed, later studies by Wright and Weller show that also CaH_2_ and BaH_2_ are active in ethylene hydrogenation but only after prior thermal treatment under high vacuum, advocating that the presence of the metallic state is of importance.^[4]^

**Scheme 1:**
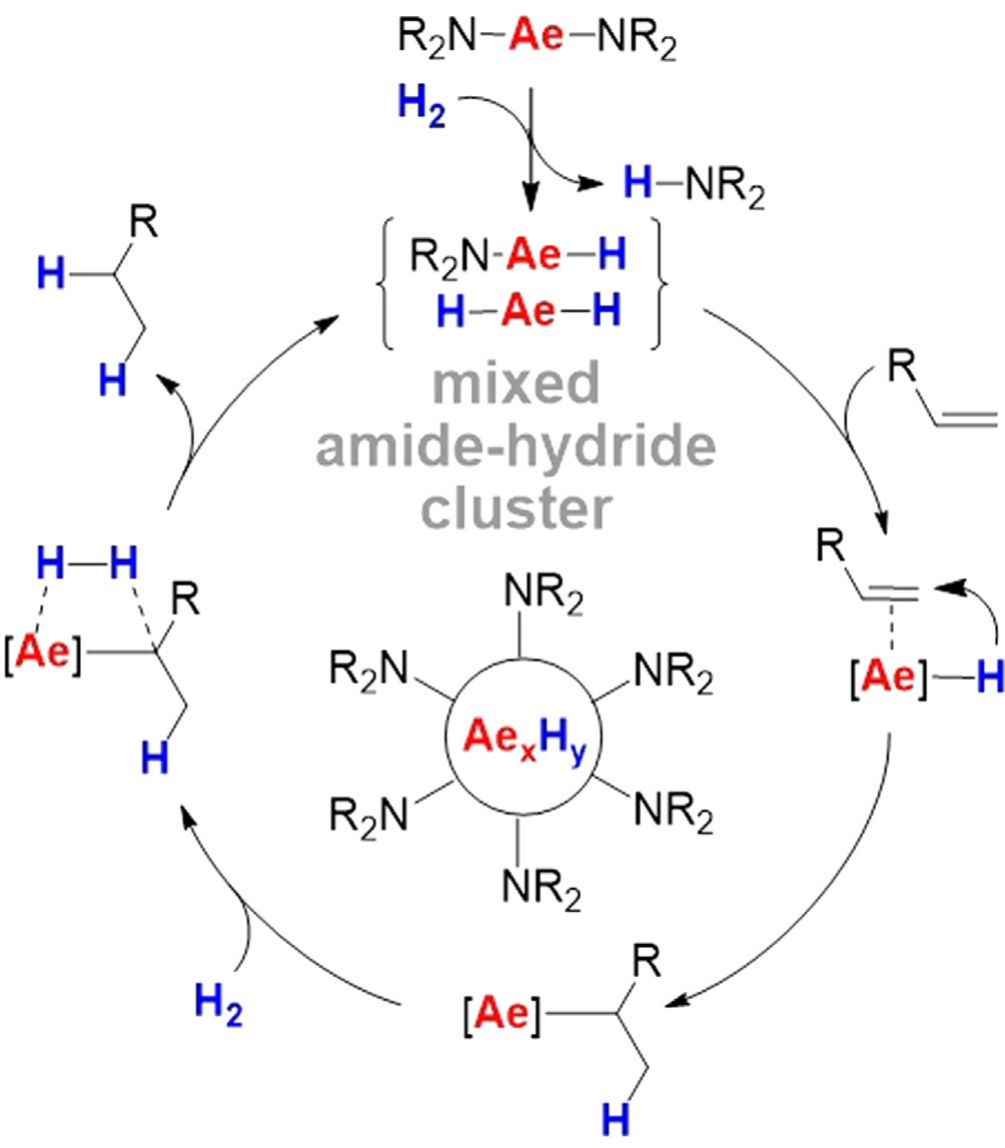
Simplified catalytic cycle for alkene hydrogenation with Ae metal amide (pre)catalysts.

Although these early reports are limited only to ethylene hydrogenation, they become topical again in light of the recent interest in alkene hydrogenation with heavier group 2 metal catalysts.[^5^, ^6^, ^7^, ^8^, ^9^] We reported alkene hydrogenation using simple Ae metal amides like Ae[N(SiMe_3_)_2_]_2_ (Ae=Ca, Sr, Ba)^[5b]^ with activities and scope sharply increasing from Ca to Ba. Contrary to expectation, these metal amides react with hydrogen to give HN(SiMe_3_)_2_ and larger metal hydride clusters of which the surface is decorated with amide ligands (Scheme 1).^[9]^ These capping ligands solubilize the Ae hydride clusters but also prevent further aggregation to insoluble (AeH_2_)_*n*_ salts. It was found that increasing the size of the amide ligand from N(SiMe_3_)_2_ to N(Si*i*Pr_3_)_2_ led to a considerable boost in catalytic activity and broadened the substrate scope from alkenes to arenes.^[5d]^ This has been rationalized by the fact that larger capping ligands like N(Si*i*Pr_3_)_2_ form smaller, more active, metal hydride clusters. Although one would expect that the catalyst activity may be increased further using even bulkier ligands, we now report that ligand‐free metallic Ba is a very simple but highly active hydrogenation catalyst.

## Results and Discussion

In order to shed light on the historically documented catalytic activity of Ba metal and BaH_2_,[^3^, ^4^] we first tested commercially available BaH_2_ (white powder, 60 mesh) in hydrogenation catalysis. Under various conditions (120–150 °C, 20–50 bar H_2_), no conversion of benzene and cyclohexene was found. Also thermal vacuum treatment of BaH_2_ (200 °C, 10^−2^ bar, 17 h) did not result in an active catalyst. Similarly, small pieces of Ba metal freshly cut under N_2_ are not catalytically active for alkene hydrogenation and it was found that commercially available Ba metal is essentially inert to H_2_ gas. This stands in strong contrast with one classical application of Ba in radio or television tubes as a getter, that is, a metal that reacts with hydrogen or other gases in order to maintain the excellent tube vacuum.^[10]^ In this role, a barium mirror is condensed on the inside of the tube as a highly activated thin metal film. With this application in mind, we activated bulk Ba metal by evaporation and cocondensation with heptane under high vacuum, using home‐build metal‐evaporation equipment as described previously.^[11]^ This gave after solvent removal an oxide‐free black Ba metal powder that is a highly potent catalyst for alkene, alkyne, imine and arene hydrogenation (Table 1 and Table 2).

**Table 1:** Hydrogenation of alkenes, alkynes and imines with activated Ba metal. Between squared brackets: results for catalyst Ba[N(Si*i*Pr_3_)_2_]_2_ (10 mol %, 6 bar, 120 °C).

Entry	Substrate	mol %	H_2_ [bar]	*T* [°C]	*t* [h]	Product(s)	Conv. [%]
1	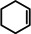	5	12	25	3 [3]	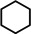	99 [99]
2		5	12	80	0.5 [3]		99 [99]
3		2.5	12	100	1 [3]		99 [99]
4^[a]^	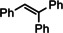	5	20	120	6 [1]	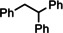	99 [99]
5	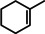	10	20	150	24 [24]	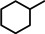	99 [81]
6^[a]^	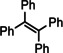	10	20	150	24 [24]	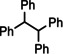	99 [73]
7	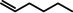	5	12	100	3 [0.5]	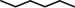	99 [99]
8	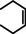	5	12	120	1 [7]	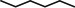	99 [99]
9	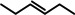	10	20	120	24 [22]	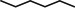	99 [99]
10	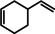	5	12	120	2.5 [3]	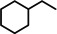	99 [99]
11^[a]^	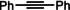	5	12	120	6 [1]	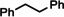	99 [99]
12	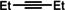	10	20	120	24 [24]	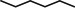	99 [99]
13	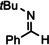	5	12	120	3.5	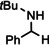	99
14	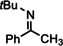	10	20	120	24	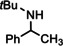	99
15^[a]^	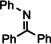	5	20	120	8	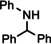	99

[a]=reactions performed in C_6_D_6_.

**Table 2:** Catalytic hydrogenation of arenes and pyridines with activated Ba metal in C_6_D_6_ (1 M, if not stated otherwise). Between squared brackets: results for catalyst Ba[N(Si*i*Pr_3_)]_2_]_2_ (10 mol %, 12 bar, 120 °C).

Entry	Substrate	mol %	H_2_ [bar]	*T* [°C]	*t* [h]	Product(s)	Conv. [%]
1^[a]^	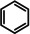	10	50	150	6 days [3 days]	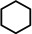	99 [18]^[b]^
2	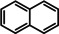	10	20	120	3.5 [2]	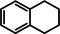	99 [99]
3	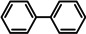	5	20	120	12 [10]	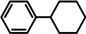	99 [99]
4	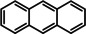	5	12	120	24 [2.5]	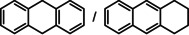	<5 % [94/2]
5	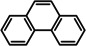	10	50	150	24 [48]	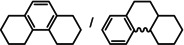	31/69^[c]^ [49/51]
6	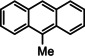	5	20	120	25 [24]	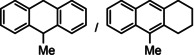	86/13 [84/15]
7	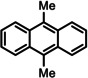	10	50	120	24	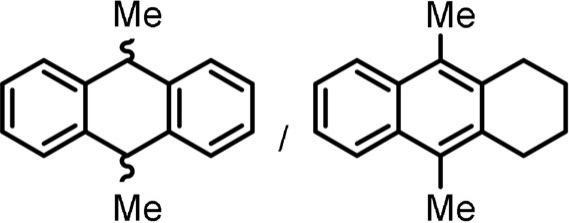	85^[c]^/12^[d]^
8	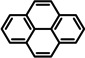	10	50	150	48 [24]	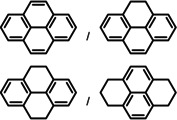	2/34/ 43/21 [94/6/ 0/0]
9	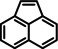	10	50	150	24 [24]	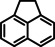	50 [0]
10	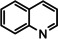	10	20	135	24	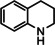	99
11	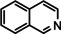	10	50	150	2.5 days	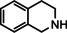	99
12	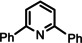	10	50	150	24	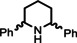	99^[c]^
13^[e]^	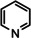	10	50	150	3.7 days	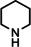	99

[a] neat; [b] Conditions: 2.5 mol % Ba[N(Si*i*Pr_3_)_2_]_2_, 50 Bar H_2_, 140 °C; [c] mixture of *cis*‐ and *trans*‐isomers; [d]+ 3 % octahydro‐dimethyl‐anthracene; [e]=1 M in C_6_D_12_.

As a first test case we chose the reduction of cyclohexene, a challenging substrate^[12]^ that previously only could be converted by the hitherto most reactive group 2 metal catalyst with a superbulky amide ligand: Ba[N(Si*i*Pr_3_)_2_]_2_.^[5d]^ Noticeably, with a catalyst loading of 5 mol % (H_2_ 12 bar, 80 °C) cyclohexene was fully reduced within 0.5 hour (Table 1, entry 1). It is even more remarkable that at room temperature cyclohexene was fully hydrogenated within three hours (entry 2) and the catalyst concentration could easily be lowered to 2.5 mol % (entry 3). Activities generally increase with increasing pressure and temperature (Figures S80,81). Table 1 demonstrates that ligand‐free Ba metal is at least as effective in hydrogenation catalysis than Ba[N(Si*i*Pr_3_)_2_]_2_; values for the latter are shown between squared brackets. Attempts to activate bulk Ba metal by dissolution in liquid ammonia and subsequent solvent evaporation gave a Ba^0^ powder that is essentially non‐active in cyclohexene hydrogenation (Table S5), implying that catalyst synthesis by the metal vapor method is crucial.

Encouraged by these preliminary results, hydrogenation of tri‐substituted alkenes was probed. While the activated (conjugated) alkene Ph_2_C=C(H)Ph could be fully converted at 120 °C (entry 4), the unactivated alkene 1‐Me‐cyclohexene needed a somewhat higher temperature of 150 °C (entry 5). For the first time, also the tetra‐substituted alkene, Ph_2_C=CPh_2_, could be quantitatively reduced with a group 2 metal catalyst (entry 6).

Further comparison (entries 7–10) shows that the catalytic performance of Ba metal is at least at par with that of Ba[N(Si*i*Pr_3_)_2_]_2_, the hitherto most active group 2 metal hydrogenation catalyst. Noticeable, is the unproblematic full conversion of non‐cyclic internal alkenes like *cis*‐ and *trans*‐3‐hexene (entries 8,9). Also di‐substituted alkynes were fully hydrogenated at rates similar to those reported for Ba[N(Si*i*Pr_3_)_2_]_2_ (entries 11,12).

For further comparison of Ba metal and Ba amide catalysts, also imine hydrogenation has been investigated (entry 13). The activity of Ba metal is comparable to that of Ba[N(SiMe_3_)_2_]_2_.^[13]^ Interestingly, while the latter catalyst could only reduce aldimines, Ba metal fully converted more challenging ketimines, further extending the applicability of group 2 metal catalysis (entry 14,15).

As a last showcase for the full scope of metallic Ba in hydrogenation catalysis, the reduction of aromatic rings was demonstrated (Table 2). Under forced conditions (150 °C, 50 bar), benzene was quantitatively converted to cyclohexane (entry 1). Although a prolonged reaction time of six days is needed, it should be noted that with Ba[N(Si*i*Pr_3_)_2_]_2_ after three days only 18 % conversion could be reached.^[5d]^ Conjugated rings in naphthalene or biphenyl reacted smoothly (entries 2,3) but anthracene gave essentially no conversion (entry 4), also not under forced conditions (150 °C, 50 bar). Since Ba amide catalysts gave fast reduction, failure of Ba metal to catalyse anthracene reduction may be related to the formation of insoluble, polymeric, {[Ba^2+^][C_14_H_10_^2−^]}_*n*_ salts. Indeed, hydrogenation of phenanthrene or more soluble alkylated anthracenes was successful (entries 5–7). While Ba[N(Si*i*Pr_3_)_2_]_2_ was not able to reduce pyrene or acenaphthylene, catalytic quantities of Ba metal reduced up to two out of four of the pyrene rings (entry 8) while also acenaphthylene could be reduced (entry 9).

One disadvantage of the bulky Ba amide catalyst Ba[N(Si*i*Pr_3_)_2_]_2_ is its inability to hydrogenate substrates that contain heteroatoms like O or N. This was attributed to substrate‐metal coordination, blocking potential coordinations sites for H_2_ activation.^[5d]^ Metallic Ba, however, is able to catalyze the hydrogenation of (iso)quinoline and pyridines (entries 10–13). Despite the somewhat longer reaction times, even pyridine itself could be fully and cleanly converted to piperidine without formation of a bipyridine by‐product.

It is clear from these data that Ba metal is a highly effective, universal, hydrogenation catalyst. In contrast, it is difficult to fully understand the underlying mechanism(s). Other than in homogeneous catalysis, in heterogenous catalysis intermediates are not easily detected and characterized. Moreover, there is a growing body of opinion that a clear distinction between homogeneous or heterogeneous catalysis is often problematic.^[14]^

**Scheme 2:**
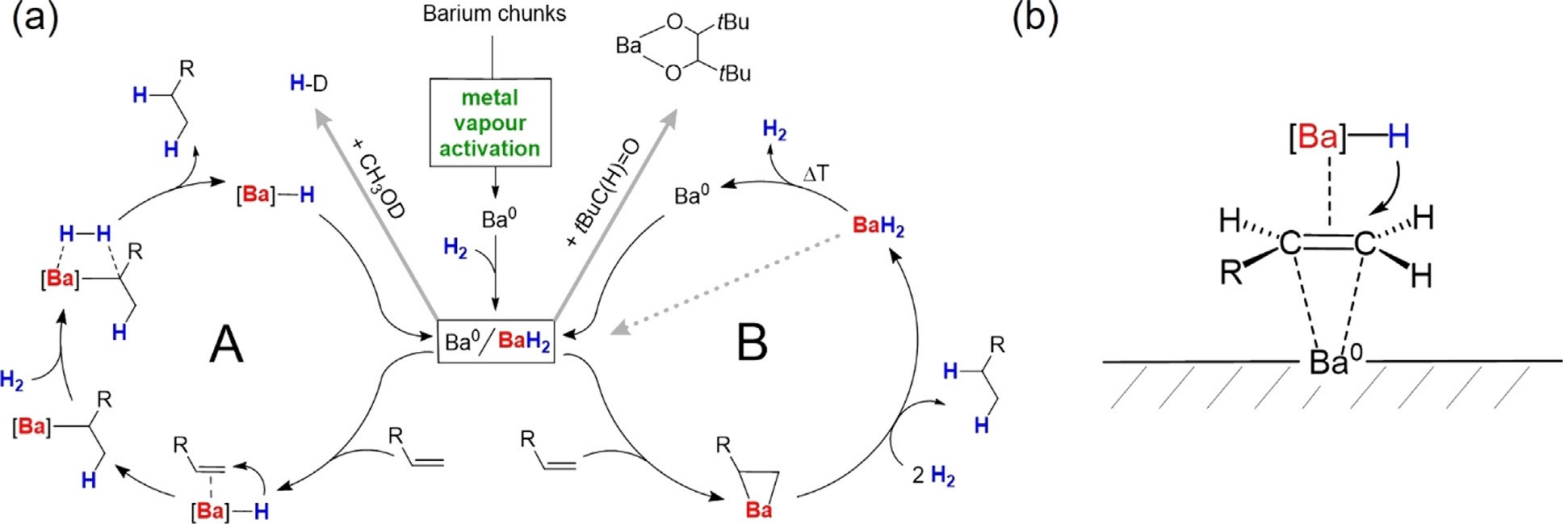
a) Formation of highly active Ba^0^ and reaction with H_2_ to a catalyst for alkene hydrogenation. Cycle A: metal hydride mechanism. Cycle B: metal mechanism. b) Proposed dual site catalysis:^[4a]^ the alkene is activated by Ba^0^ and attacked by Ba hydride.

Given the low ionization potential of Ba, the logical first step is the conversion of Ba^0^ with H_2_ to BaH_2_. This reaction has been reported to start at 120 °C^[15]^ but most recent literature mention an onset point of 80 °C.^[16]^ This would not explain the rather facile cyclohexene hydrogenation at room temperature (Table 1, entry 2). It is, however, also known that oxide‐free thin films of Ba absorb H_2_ already at room temperature which is the basis of its application as a H_2_ getter.^[17]^ Reacting black Ba powder, activated by cocondensation, overnight in a reactor with 20 bar H_2_, either at 20 °C or 120 °C, led in both cases to a color change to grey. It is well‐known that, although barium hydride is referred to as BaH_2_, often this composition is never attained and generally substoichiometric compounds are formed.^[18]^ Therefore, we presume the product to be a Ba^0^/BaH_2_ mixture. Indeed, reaction with CH_3_OD led to formation of H‐D, which proves the presence of hydride. On the other hand, reaction with benzophenone gave a color change to intense deep purple and addition of pivaldehyde led to pinacolate coupling, both providing evidence for metallic Ba; Scheme 2 a (Figures S67–70).

Based on an insoluble Ba^0^/BaH_2_ catalyst, two different catalytic cycles could be envisioned (Scheme 2 a): (A) the classical hydride cycle in which alkene insertion is followed by hydrogenolysis, or (B) the Ba metal cycle in which Ba first reacts with the alkene to a metallacycle after which the product is formed by hydrogenolysis.

The hydride cycle A is proposed for isolated (unactivated) alkenes, for example, 1‐hexene. This cycle might operate on the metal surface but, as previously described for Pd metal catalyzed reactions,^[14c]^ during catalysis also smaller Ba hydride clusters, solubilized by organic groups, may go in solution. After catalytic hydrogenation generally grey turbid suspensions were obtained. Separation of the mother liquor by filtration and evaporation of all volatiles (solvent, educt and product) did not lead to visible residues and the residue can be reused as a catalyst, which suggests a heterogenous mechanism. This is supported by mercury poisoning: addition of liquid Hg to the Ba metal catalyst killed all activity (Ba amalgam reacts with H_2_ at 1400 °C).^[18]^ Addition of Hg after reaction of Ba with H_2_, however, keeps the catalyst active. We propose therefore a heterogenous Ba^0^ catalyst with Ba hydride functions at its surface.

The Ba metal cycle B could be operative for easily reducible substrates like conjugated systems (e.g. Ph_2_C=CPh_2_), extended arenes, alkynes, imines or pyridines. This is exemplified by the well‐known oxidative addition of Ae metals to dienes^[19]^ which especially for the heavier Ae metals (Ca, Sr, Ba) already proceeds at room temperature.^[20]^ Since also imines^[21]^ and extended π‐systems (like anthracene)^[22]^ react with the heavier Ae metals to dianionic ions, cycle B may also in these cases be operative. Note that BaH_2_ is formed in the last step of cycle B. To reenter the cycle as Ba^0^, hydrogen must be released. Although reductive elimination (BaH_2_ → Ba^0^ + H_2_) starts at around 330 °C,^[23]^ similar as for MgH_2_ decreasing particle size will lower the onset temperature.^[24]^ The early work from Weller et al. shows that BaH_2_ can already be partially converted to Ba^0^ above 100 °C.^[4a]^ Indicative for this step is the fact that substrates for which the metal mechanism is expected often need the somewhat higher temperature of 150 °C. A strong argument for metal cycle B is the fact that activated Ba^0^ catalyzes hydrogenation of substrates that are fully inert to Ba amide/hydride catalysts (e.g. Table 1. entries 13–15, Table 2. entries 9–13). It is, however, also possible that in some cases both cycles operate in which case regeneration of Ba^0^ is no longer a requirement.

**Figure 1:**
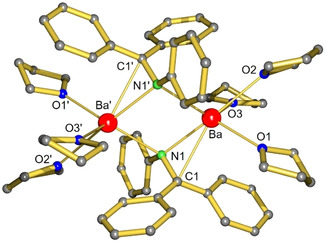
The centrosymmetric crystal structure of [(Ph_2_CNPh)Ba⋅(THF)_3_]_2_; H atoms omitted for clarity. Selected bond distances [Å] and angles [°]: Ba–C1 2.953(2), Ba–N1 2.711(2), Ba–N1′ 2.773(2), Ba–O 2.755(2)–2.821(2); C1‐Ba1‐N1 29.3(7).

The feasibility of the Ba metal mechanism B was validated by stoichiometric conversions. Reaction of Ph_2_C=NPh with activated Ba^0^ metal gave a deep red solution that is typical for the Ph_2_CNPh^2−^ dianion.^[21]^ The resulting azametallacyclopropane complex crystallized as the dimer [(Ph_2_CNPh)Ba⋅(THF)_3_]_2_ which could be described as two C‐N‐Ba metallacycles connected by bridging nitrogen atoms (Figure 1). The doubly charged Ph_2_CNPh^2−^ anion is highly reactive and in contact with H_2_ the expected amine Ph_2_C(H)N(H)Ph was cleanly formed already at room temperature (Figures S75,76), justifying this route.

As a third possibility, one could discuss a mechanism that is a hybrid between cycles A and B (Scheme 2 b): sorption of an alkene at a Ba^0^ surface activates the C=C bond for nucleophilic attack by a neighbouring Ba‐H functionality. Adsorption of ethylene on a Ca metal surface was already described as early as 1923^[2]^ and also has been established for metallic Ba.^[25]^ Mentionable is the recent recognition that *d*‐orbitals play a role in the chemistry of the heavier Ae metals Ca, Sr and Ba. In metallic form they mimic transition metals and low temperature matrix‐isolation of Ae(CO)_8_, Ae(N_2_)_8_ and Ae(C_6_H_6_)_3_ complexes has been reported.^[26]^ Similarity of Ca and Ba with the transition metals was noticed already by Wright and Weller in the 1950’s. Their comprehensive investigations support a catalyst consisting of Ba^0^ and BaH_2_ which in the presence of H_2_ are in a temperature dependent equilibrium.^[4]^ Thermal treatment of BaH_2_ under high vacuum gave in some cases a catalyst that hydrogenates ethylene already at −78 °C.^[4b]^ They stress the point that both, metal and metal hydride, need to be present and “*the catalytic effect occurs at an interface between free metal and metal hydride”*.^[4a]^ These hot spots are called “*dual sites*” and, already at this very early stage, they explain the high activity of Ca and Ba catalysts with “*some overlap of s‐, p‐ and d‐bands*”.^[4a]^

**Scheme 3:**
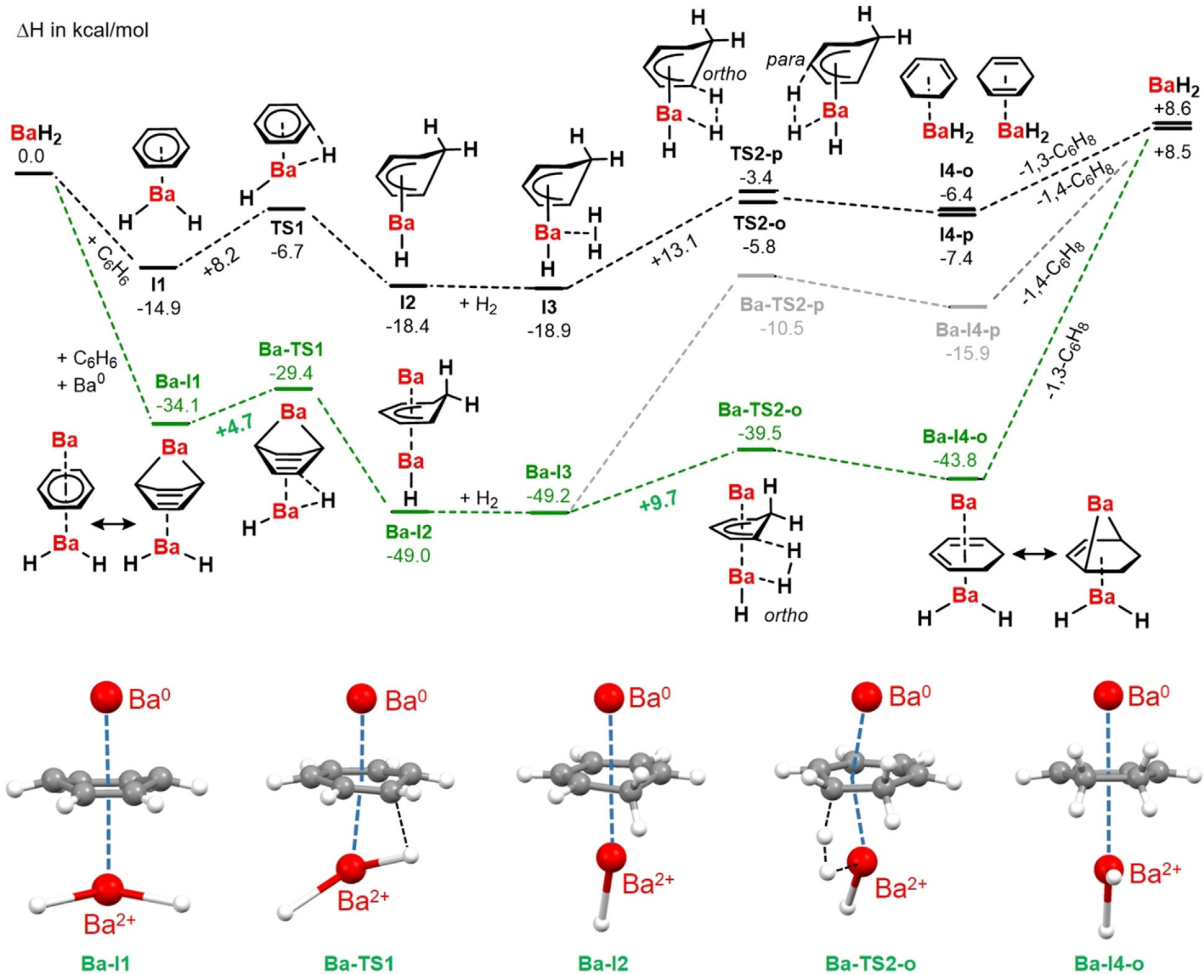
Energy profiles (Δ*H* in kcal mol^−1^) for the hydrogenation of benzene (B3PW91/def2TZVPP + GD3BJ). Black: pathway for catalyst BaH_2_. Green: pathway for catalyst Ba^0^/BaH_2_. Some intermediates for the Ba^0^ assisted route are shown at the bottom.

It is difficult to verify heterogeneous catalytic pathways. The possibility for a Ba^0^/BaH_2_ pathway needs detailed investigations and advanced calculational studies at surfaces. Although gas phase calculations cannot model complicated heterogeneous systems, they do provide insights in basic steps at an atomic level. Here we present preliminary DFT calculations on very simple model systems. These indicate the activating influence of Ba^0^ in benzene hydrogenation with BaH_2_ (Scheme 3). The normal route (in black) shows benzene hydrogenation with BaH_2_. After formation of a benzene⋅⋅⋅BaH_2_ complex (**I1**), an activation energy of 8.2 kcal mol^−1^ is needed to reach the transition state (**TS1**). The Meisenheimer anion formed during this process is bound to Ba (**I2**). Hydrogenolysis in *ortho*‐position (**TS2‐o**) is slightly preferred over attack in the *para*‐position (**TS2‐p**). Note that reduction of the first C=C bond in benzene is endothermic. The exothermic hydrogenation of the two remaining C=C bonds, however, make benzene hydrogenation overall exothermic.^[5d]^

The same mechanism was calculated in presence of a Ba^0^ atom (in green). Due to its high reducing power, an interesting starting complex is formed (**Ba‐I1**) in which the benzene ring is not flat but slightly puckered to a boat form. Different C−C distances in the ring (1.455 Å/1.520 Å) reveal charge transfer from Ba to benzene and the system could be described as having partial Ba^2+^(1,3‐cyclohexadiene^2−^) character. Similar distortions have been observed in Ae(C_6_H_6_)_3_ complexes.^[26c]^ NPA charges (Figure S88) confirm a substantial charge transfer from the Ba atom (+1.48) to the ring (−1.50) while the BaH_2_ unit is close to neutral (Ba +1.53, H −0.76). This reactivity could be compared to the formation of Mg‐anthracene,^[19b]^ the difference being that benzene is much harder to reduce. This process is supported by BaH_2_ coordination which acts as a Lewis acid that stabilizes the anti‐aromatic 1,3‐cyclohexadiene^2−^ anion.^[27]^ The activation barrier for hydrogenation of 4.7 kcal mol^−1^ is nearly halved compared to that for the Ba^0^‐free route (8.2 kcal mol^−1^). Partial loss of aromaticity in the benzene ring assists the hydrogenation reaction. Also the activation energy for subsequent hydrogenolysis is lowered by additional interaction with Ba^0^ (+9.7 vs. +13.1 kcal mol^−1^). Note that this effect is only noticeable for hydrogenation in *ortho*‐position. Hydrogenolysis in *para*‐position leads to a drastic increase of the barrier. The preference for the *ortho*‐position can be explained by the favorable interaction of Ba^0^ with conjugated dienes (**Ba‐I4‐o**) compared to isolated alkenes (**Ba‐I4‐p**). Complex **Ba‐I4‐o** should rather be described as the product of 1,4‐addition of Ba^0^ to a diene.[^19^, ^20^] The positive charge of +1.55 on Ba and negative charge of −1.57 on the ring confirm this view. Although these models are rudimentary, they reinforce the thesis that Ba^0^ can have an accelerating effect on catalytic hydrogenation.

## Conclusion

Metallic barium is shown to be an all‐round catalyst for effective hydrogenation of challenging alkenes, alkynes, arenes and imines. In strong contrast with our previous approach to increase the activity of Ba amide catalysts by increasing ligand size, use of metallic Ba as a versatile, highly active, early main group metal hydrogenation catalyst not only extends the scope of substrates but is also remarkably simple. Although the metal needs to be activated, the current method represents a welcoming short‐cut in respect to the syntheses of superbulky Ba amide catalysts. Precise understanding of the fundamental mechanisms is at this stage difficult but the idea that the combination of Ba metal and Ba hydride dual sites is key to its activity has been demonstrated by DFT calculations on a simplified model system. This important aspect will be comprehensively investigated and exploited further in future research.

## Conflict of interest

The authors declare no conflict of interest.

## Supporting information

As a service to our authors and readers, this journal provides supporting information supplied by the authors. Such materials are peer reviewed and may be re‐organized for online delivery, but are not copy‐edited or typeset. Technical support issues arising from supporting information (other than missing files) should be addressed to the authors.

Supplementary
