# Supplementary material for: Metallic Barium: A Versatile and Efficient Hydrogenation Catalyst
Source: Angew Chem Int Ed Engl. 2020 Dec 21;60(8):4252–8. doi: 10.1002/anie.202014326 (PMC7898525; doi:10.1002/anie.202014326)
Supplement: Supplementary file 1 — Supplementary [file ANIE-60-4252-s001.pdf]

Angewandte Chemie
Eine Zeitschrift der Gesellschaft Deutscher Chemiker

Supporting Information

# Metallic Barium: A Versatile and Efficient Hydrogenation Catalyst

*Philipp Stegner, Christian Färber, Ulrich Zenneck, Christian Knüpfer, Jonathan Eyselein, Michael Wiesinger, and Sjoerd Harder**

anie_202014326_sm_miscellaneous_information.pdf

# Supporting Information:

## Contents


**1. Supporting Experimental Data** S3

**1.1 General Experimental Procedures** S3

**1.2 Metal Vapour Synthesis** S5

**1.3 Catalytic Hydrogenation of Alkenes, Alkynes and Imines** S6

**1.4 Catalytic Hydrogenation of Arenes** S36

**1.5 Deuteration Experiments** S68

**1.6 Stoichiometric Experiments** S71

**1.7 Crystal Structure Determination** S89

**1.8 Temperature and Pressure Dependency** S93

**1.9 Ba Activation using Ammonia** S96

**1.10 DFT calculations** S104

**2. References** S114

# 1. Supporting Experimental Data

## 1.1 General Experimental Procedures

All experiments were conducted under an inert nitrogen atmosphere using standard Schlenk and glovebox techniques (MBraun, Labmaster SP). Toluene, *n*-hexane and benzene were degassed with nitrogen, dried over activated aluminium oxide (Solvent Purification System: Pure Solv 400−4−MD, Innovative Technology) and stored over 3Å molecular sieves under $N_2$. Heptane was purchased from Sigma-Aldrich, degassed and dried over 3Å molecular sieves under $N_2$. THF was degassed, refluxed over a sodium mirror, distilled under $N_2$ and stored over 3Å molecular sieves under $N_2$. Benzene-$d_6$ (99.6% D, Sigma-Aldrich), THF-$d_8$ (99.6% D, Sigma-Aldrich) and cyclohexane-$d_{12}$ (99.6% D, Sigma-Aldrich) were dried over 3Å molecular sieves and stored under $N_2$.

NMR spectra were recorded with a Bruker Avance III HD 400 MHz or a Bruker Avance III HD 600 MHz spectrometer. Crystal structures have been measured on a SuperNova (Agilent) diffractometer with dual Cu and Mo microfocus and an Atlas S2 detector. Elemental analyses were performed with a Hekatech Eurovector EA3000 analyzer.

GC-MS measurements were performed on a Thermo Scientic™ Trace™ 1310 gaschromatography system (carrier gas: helium) with detection by a Thermo Scientic™ ISQ™ LT single quadrupole mass spectrometer. A Thermo Scientific™ TraceGOLD™ TG-5SilMS GC column or Phenomenex® ZebronTM ZB-5 GC column of the dimensions 0.25mm x 30m with a film thickness of 0.25 µm was used. The samples (1µL) were injected with an instant connect-SSL module in the split mode (injector temperature: 280°C). Temperature programs were started at 40°C followed by heating ramps, optimized for the separation problem, until 280 °C. Baseline separation of each analyte was achieved by choosing the different temperature programs. The molecular identities were confirmed by comparison with entries in the NIST/EPA/NIH mass spectral library (v2.2, built June 10 2014).

All hydrogenation experiments were carried out on a high vacuum Schlenk line attached to a hydrogen gas cylinder with a pressure regulator (1-50 bars) using high-pressure miniature autoclaves (15 mL capacity) made of stainless steel (Amtech). Autoclaves were previously dried by heating in an oven at 80°C for at least 12 h. Research grade hydrogen gas ($H_2$; Air Liquide, purity: 99.999%) was used without additional purification.

The liquid unsaturated substrates cyclohexene (Fluka, 99%), 1-hexene (Acros Organics, 97%), 4-vinyl-1-cyclohexene (TCI Chemicals, >95%), *cis*-3-hexene (TCI Chemicals, 97%), *trans*-3-hexene (TCI Chemicals, 97%), 1-methyl-1-cyclohexene (Alfa Aesar, 96%), *N*-benzylidene-*tert*-butylamine (Sigma-Aldrich, 98%) and hex-3-yne (Sigma-Aldrich, 99%) were dried via stirring over freshly ground $CaH_2$ at room temperature for at least two days, distilled under reduced pressure and stored in an $N_2$-filled glovebox over activated 3Å molecular sieves prior to usage. *N*-(tert-butyl)-1-phenylethan-1-imine was synthesized according to a literature procedure[S1] and dried by stirring over freshly ground $CaH_2$. Subsequently the ketimine was distilled under reduced pressure and stored in an $N_2$-filled glovebox over activated 3Å molecular sieves prior to usage. The solid unsaturated substrates triphenylethylene (Alfa Aesar, 98%), tetraphenylethylene (Sigma-Aldrich, 98%) and diphenylacetylene (Sigma-Aldrich, 98%) were dried under high vacuum for two days at 60°C and stored in a $N_2$-filled glovebox. *N*,1,1-triphenylmethanimine was synthesized according to a literature known procedure[S2], and dried by stirring over freshly ground $CaH_2$ in THF. Subsequently the solvent was removed and the compound was dried under reduced pressure.

The aromatic hydrocarbons naphthalene (Sigma-Aldrich, 99%), biphenyl (Sigma-Aldrich, >99%), phenanthrene (Alfa Aesar, 98%), acenaphthylene (TCI Chemicals, >94%), anthracene (Sigma-Aldrich, 97%), 9-methylanthracene (TCI Chemicals, 98%), 9,10-dimethylanthracene (TCI Chemicals, 98%) and pyrene (Alfa Aesar, 98%) were obtained commercially, sublimed under reduced pressure and stored in a glovebox under a $N_2$ atmosphere prior to usage. The aromatic heterocycles pyridine (Acros Organics, >99%), quinoline (TCI Chemicals, >97%) and *iso*-quinoline (Acros Organics, >97%) were dried via stirring over freshly ground $CaH_2$ at room temperature for at least three days, distilled under reduced pressure and stored in an $N_2$-filled glovebox over 3Å molecular sieves

prior to usage. 2,6-diphenylpyridine (TCI Chemicals, >99%) was dissolved in benzene and dried via stirring over $CaH_2$ for one week at room temperature. The $CaH_2$ was filtered off, benzene was removed under reduced pressure and the substance was dried at 60°C. Other commercial reagents were used without further purification.

## 1.2 Metal Vapour Synthesis

**Activation of Ba metal:**

Cocondensation reactions were performed with a self-made metal vapor reactor equipped with a 2 L reaction flask, an effective vacuum pump system consisting of a rotary vane pump as backing combined with an oil diffusion pump and different manifolds on the top of the apparatus for adding solvent (matrix) and a nitrogen inlet.[S3] The design includes an inner heating wire placed in the centre of the flask which holds an aluminium oxide container.

The used Ba (Barium rods, 99+%, approximately 2 cm in diameter, in mineral oil, ABCR) were washed with hexane and dried under vacuum prior to usage. Subsequently the barium rods were divided in small pieces (3.50 g, 25.6 mmol) and filled in the aluminium oxide container which was placed in the metal vapour reactor. The reactor was evacuated at high vacuum ($10^{-5}$ mbar) while the heating wire was heated up to 350°C for ca. 2 h. This generates radiant heat which warms up the reaction flask and dries the glass surface. After cooling the flask to room temperature it was placed in a liquid nitrogen bath. Subsequently, the aluminium oxide container was heated to circa 800 °C ($10^{-4}$ mbar) while heptane (ca. 150 mL) was introduced. Both, the heptane and barium vapors, condensed together onto the glass surface of the reactor forming a black layer. The cocondensation process was stopped after all solvent had been condensed into the flask (ca. 1 h). Typically, also almost all barium had been evaporated while barium oxide residues stayed behind in the aluminum oxide container. The Ba-heptane-suspension was transferred via syringe and finely divided, black barium powder was isolated by centrifugation in a Schlenk centrifuge. Subsequent drying in vacuum over two days yielded a dry, fluffy, dark black, highly pyrophoric powder that was stored in a glove box

under $N_2$. The powder can be stored under $N_2$ at room temperature without loss of activity over several weeks. Yield: 2.50 g, 18.3 mmol, 72 %.

## 1.3 Catalytic Hydrogenation of Alkenes, Alkynes and Imines

**General procedure for the catalytic hydrogenation of alkenes, alkynes and imines.**

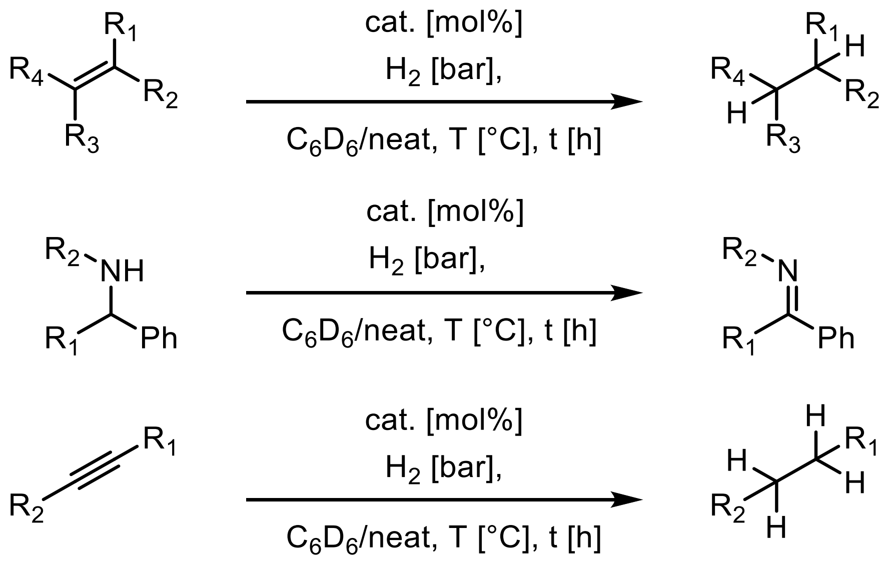


**Figure S1: Catalytic hydrogenation of alkenes, alkynes and imines.**

An oven-dried miniature stainless steel autoclave (15mL) containing a magnetic stir bar was charged with the Ba(0) pre-catalyst (5 mol% or 10 mol%) and the substrate of interest in an $N_2$-filled glovebox. The reactions were performed neat or as $C_6D_6$ solution (1 M, 1 mL). For reactions in $C_6D_6$ generally the following quantities were used: 5 mol% catalyst (0.05 mmol, 6.9 mg) or 10 mol% catalyst (0.10 mmol, 13.7 mg) and 1.0 mmol of substrate. For substrates that were converted neat following quantities were generally used: 5 mol% catalyst (0.25 mmol, 34.5 mg) or 10 mol% catalyst (0.50 mmol, 69 mg) and 5.0 mmol of substrate. The tightly sealed pressure vessel was transferred out of the glovebox and connected to a $H_2$ gas manifold. The supply line was evacuated for 1 minute and subsequently purged with dry gas. This process was repeated for three times using $N_2$ and for three further times using $H_2$ to ensure the exclusion of air and moisture. The reactor was then pressurized with $H_2$ (6 to 50 bar) and heated to the desired temperature (25 to 150 °C) in a pre-heated aluminum metal block. After stirring the reaction mixture for the indicated amount of time, the autoclave was cooled to room temperature in a water

bath and subsequently vented. The reaction mixture was filtered through a glass microfiber filter in a Pasteur pipette and the crude filtrate was analyzed by $^1H$ NMR spectroscopy without additional purification. In case of neat reactions, an aliquote (0.1 mL) was diluted in $C_6D_6$ and after filtration used for $^1H$ NMR analysis without additional purification. The products were identified by comparison of the obtained $^1H$ NMR spectra with corresponding data reported in literature.[S4] The conversion of substrates was estimated via integration of characteristic $^1H$ NMR resonances of the products and their residual counter parts. When using $C_6D_6$ as solvent, a significant degree of deuterium incorporation in the hydrogenated products, resulting from H/D isotope exchange by solvent activation, was observed. Therefore product composition was additionally analyzed by GC/MS if necessary. Yields were determined using the GC/MS trace, via integration of the peaks corresponding to the hydrocarbon products against their unsaturated analogues. Reactions times for essentially full conversion were optimized in 30 min intervals.

**Table S1.** Catalytic alkene hydrogenation; reactions performed neat if not stated otherwise.

| Entry | Substrate | mol% | $H_2$ [bar] | T [°C] | t [h] | Product(s) | Conv. [%] |
|---|---|---|---|---|---|---|---|
| **1** | | 5 | 12 | 25 | 3 [3]* | | 99 [99]* |
| **2** | 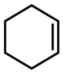 | 5 | 12 | 80 | 0.5 [3]* | 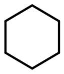 | 99 [99]* |
| **3** | | 2.5 | 12 | 100 | 1 [3]* | | 99 [99]* |
| **4** | 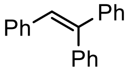 | 5 | 20 | 120 | 6 [1]* | 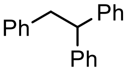 | 99[a] [99]* |
| **5** | 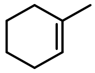 | 10 | 20 | 150 | 24 [24]* | 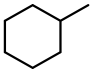 | 99 [81]* |
| **6** | 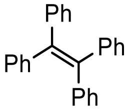 | 10 | 20 | 150 | 24 [24]* | 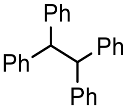 | 99[a] [73]* |
| **7** | 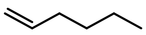 | 5 | 12 | 100 | 3 [0.5]* |  | 99 [99]* |
| **8** | 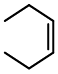 | 5 | 12 | 120 | 1 [7]* |  | 99 [99]* |
| **9** | 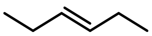 | 10 | 20 | 120 | 24 [22]* |  | 99 [99]* |
| **10** | 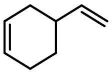 | 5 | 12 | 120 | 2.5 [3]* | 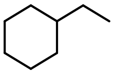 | 99 [99]* |
| **11** | 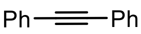 | 5 | 12 | 120 | 6 [1]* | 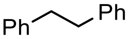 | 99[a] [99]* |
| **12** | 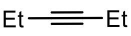 | 10 | 20 | 120 | 24 [24]* |  | 99 [99]* |
| **13** | 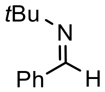 | 5 | 12 | 120 | 3.5 | 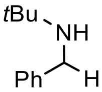 | 99 |
| **14** | 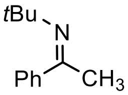 | 10 | 20 | 120 | 24 | 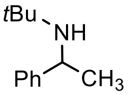 | 99 |
| **15** | 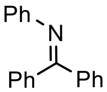 | 5 | 20 | 120 | 8 | 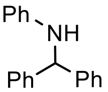 | 99 |

[a] = reactions performed in $C_6D_6$; * = 10 mol% Ba[N(Si*i*Pr$_3$)$_2$]$_2$; 6 Bar $H_2$, 120°C, 1M in $C_6D_6$.

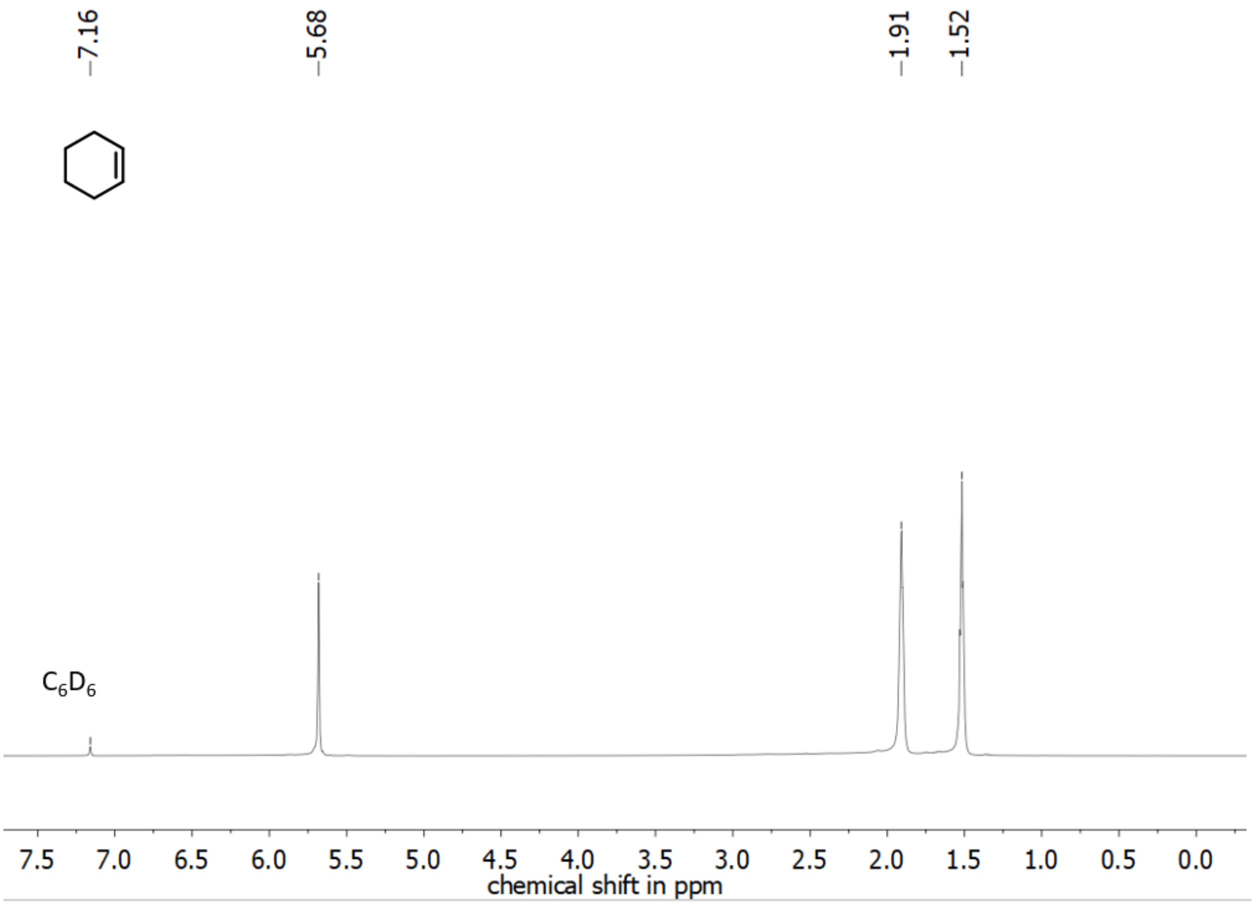


**Figure S2: $^{1}$H NMR spectrum (600 MHz, $C_6D_6$, 25°C) of cyclohexene.**

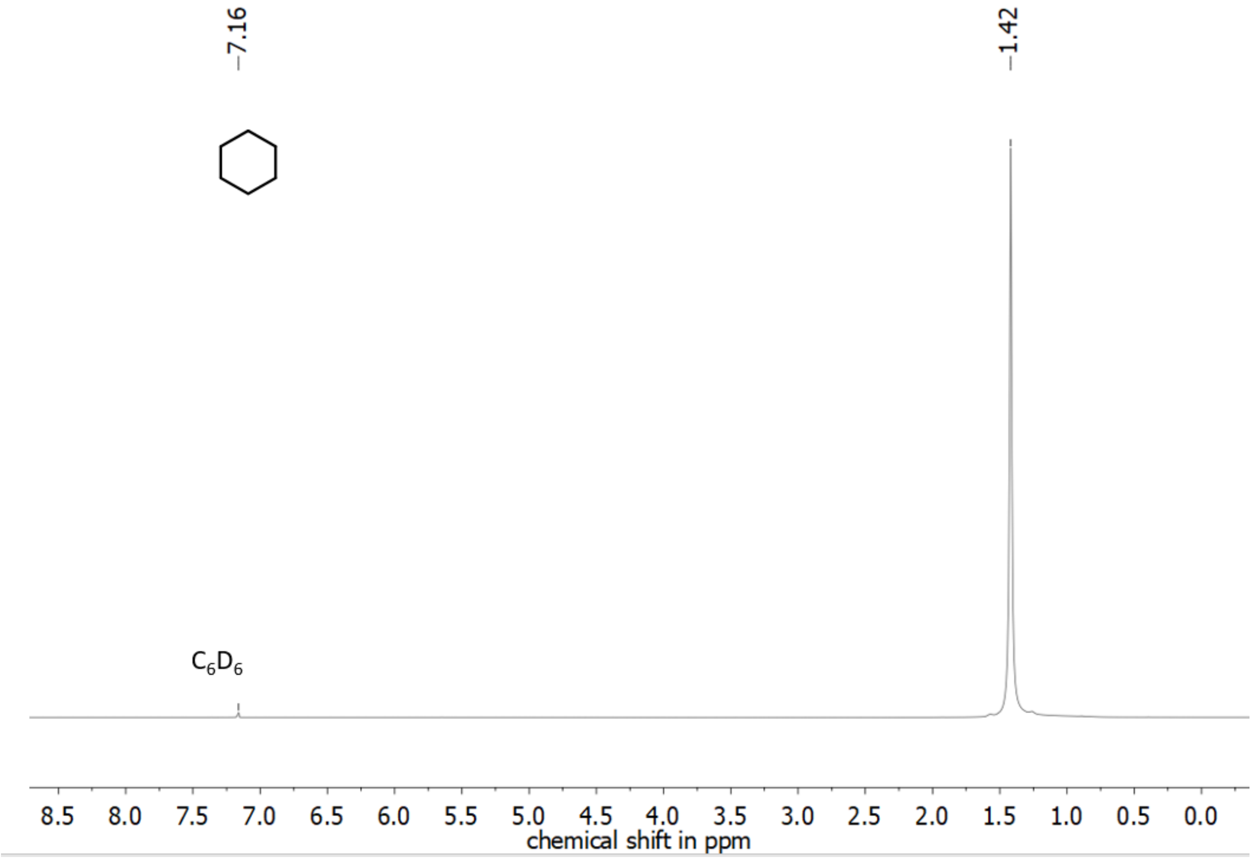


**Figure S3: $^{1}H$ NMR spectrum (600 MHz, $C_6D_6$, 25°C) of cyclohexene after catalytic hydrogenation (neat) using 5 mol% Ba(0) and 12 bars of $H_2$ at 25°C (3 h).**

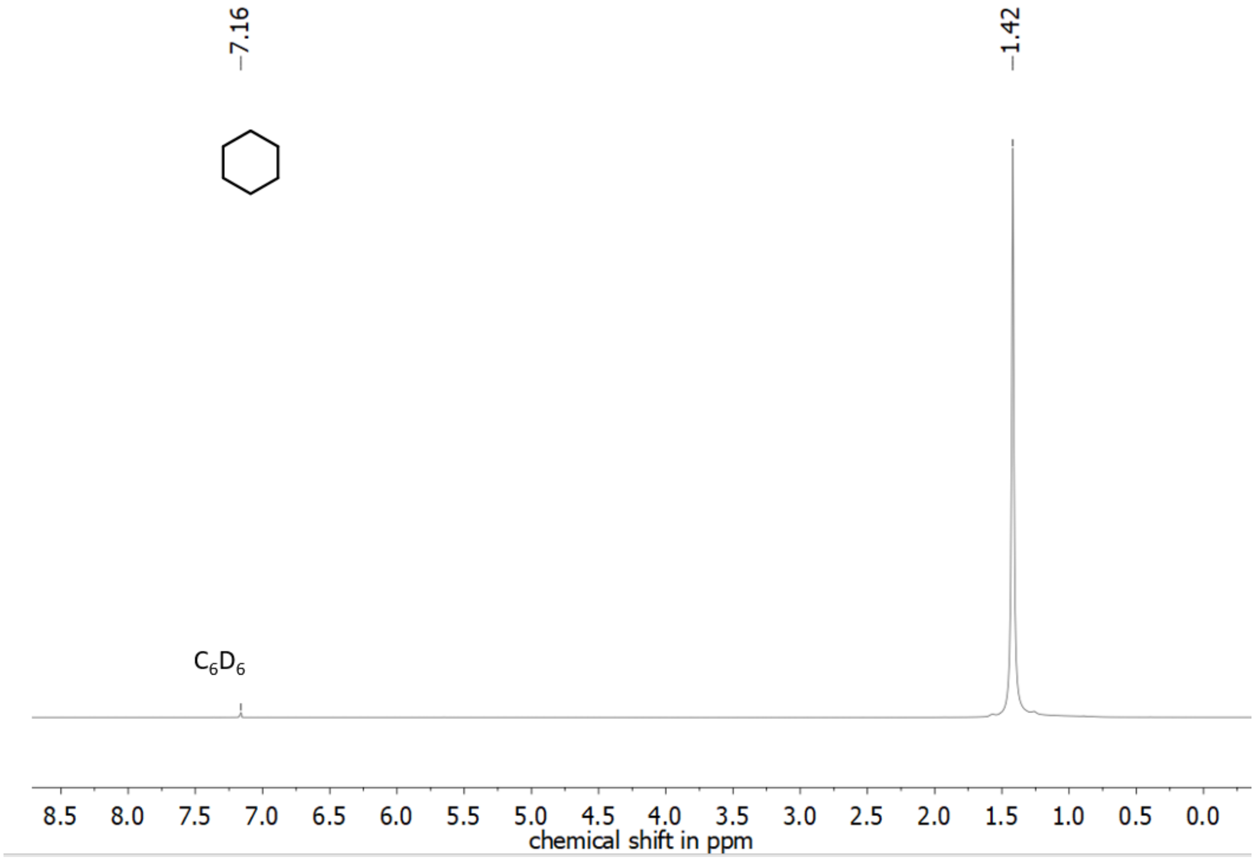


**Figure S4: $^{1}$H NMR spectrum (600 MHz, $C_6D_6$, 25°C) of cyclohexene after catalytic hydrogenation (neat) using 5 mol% Ba(0) and 12 bars of $H_2$ at 80°C (0.5 h).**

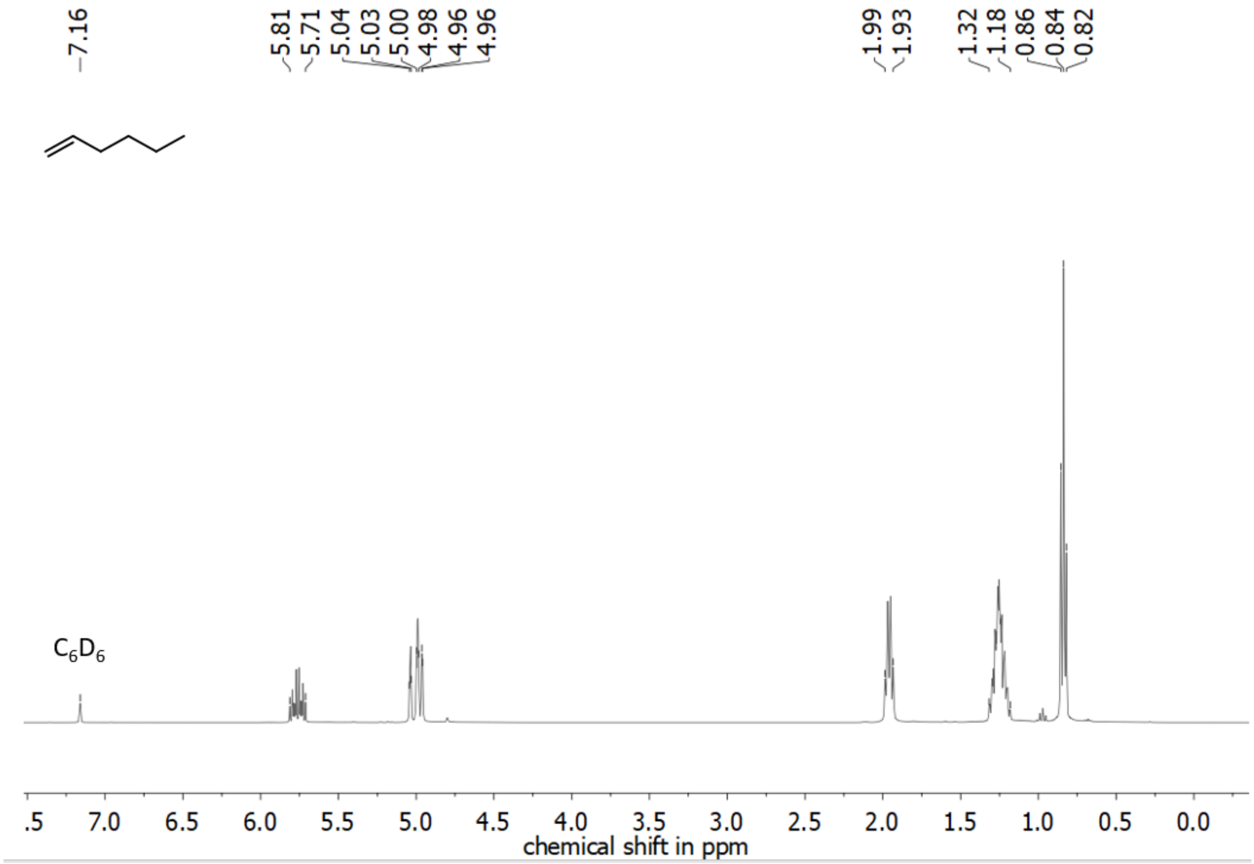


**Figure S5: $^{1}$H NMR spectrum (600 MHz, $C_6D_6$, 25°C) of 1-hexene.**

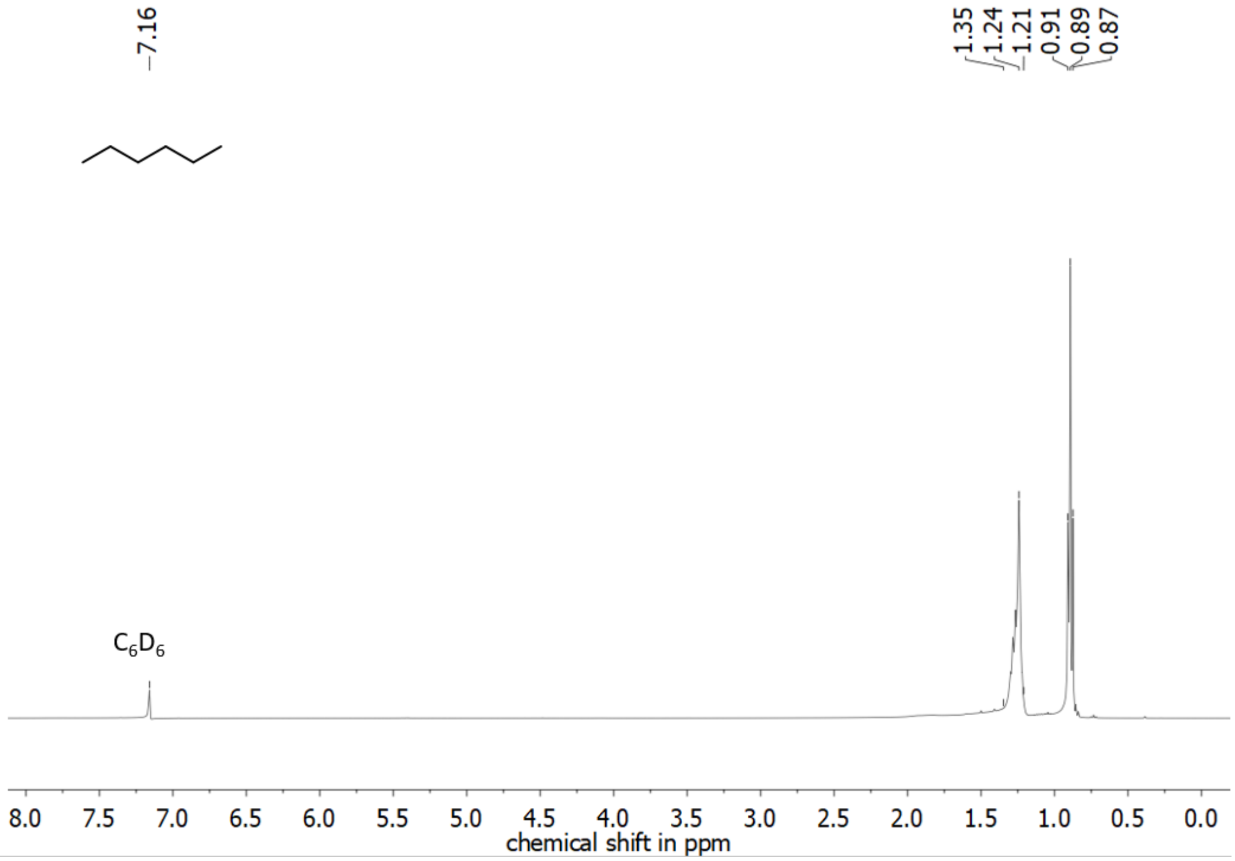


**Figure S6: $^{1}$H NMR spectrum (600 MHz, $C_6D_6$, 25°C) of 1-hexene after catalytic hydrogenation (neat) using 5 mol% Ba(0) and 12 bars of $H_2$ at 100°C (3 h).**

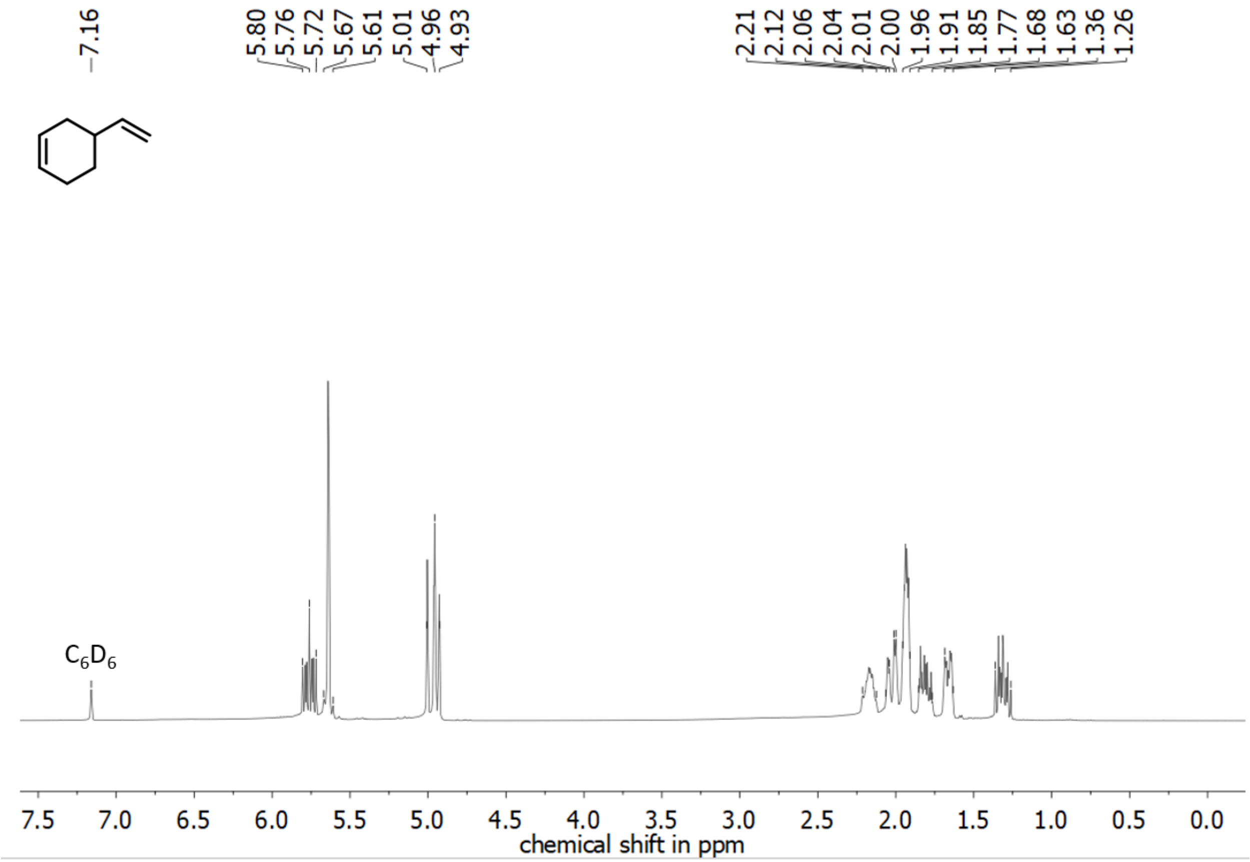


**Figure S7: $^1$H NMR spectrum (600 Mhz, $C_6D_6$, 25°C) of 4-vinyl-cyclohexene.**

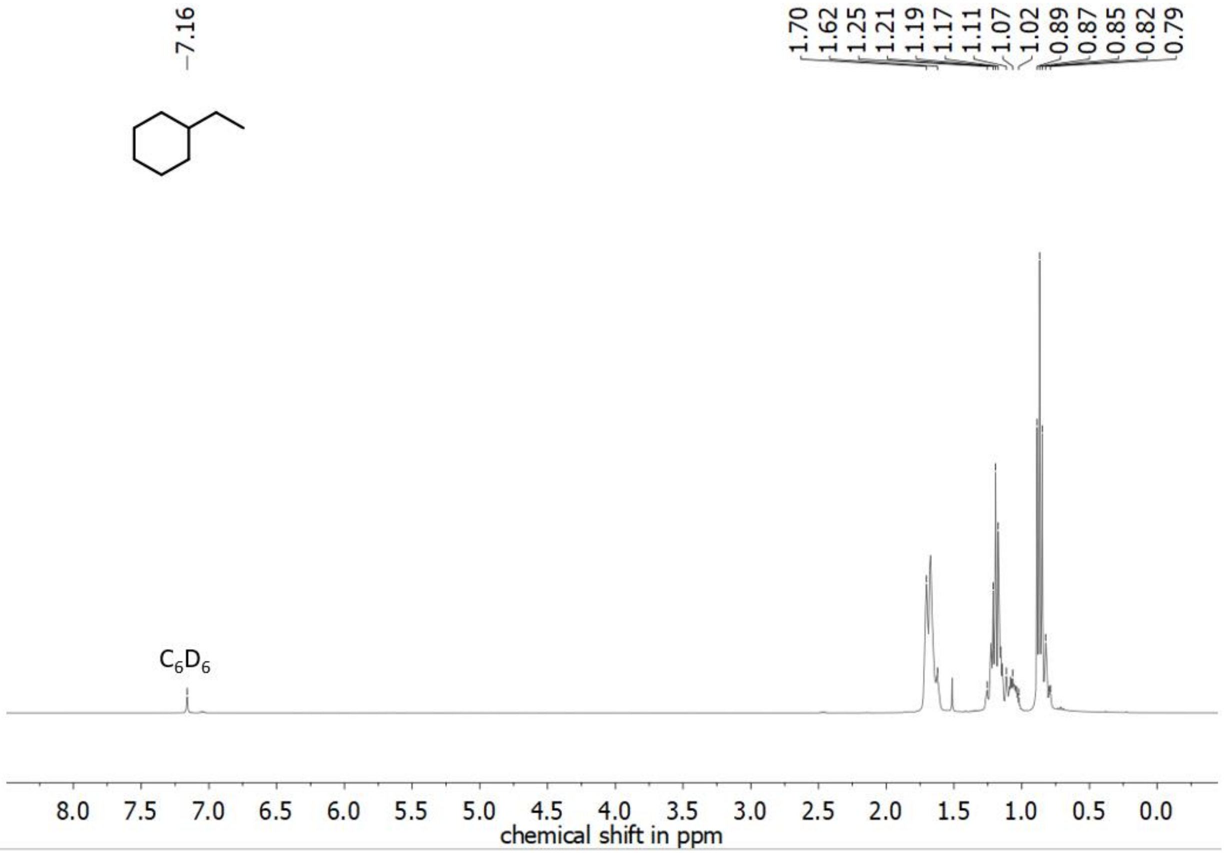


**Figure S8: $^1$H NMR spectrum (600 MHz, $C_6D_6$, 25°C) of 4-vinyl-cyclohexene after catalytic hydrogenation (neat) using 5 mol% Ba(0) and 12 bars of $H_2$ at 120°C (2.5 h).**

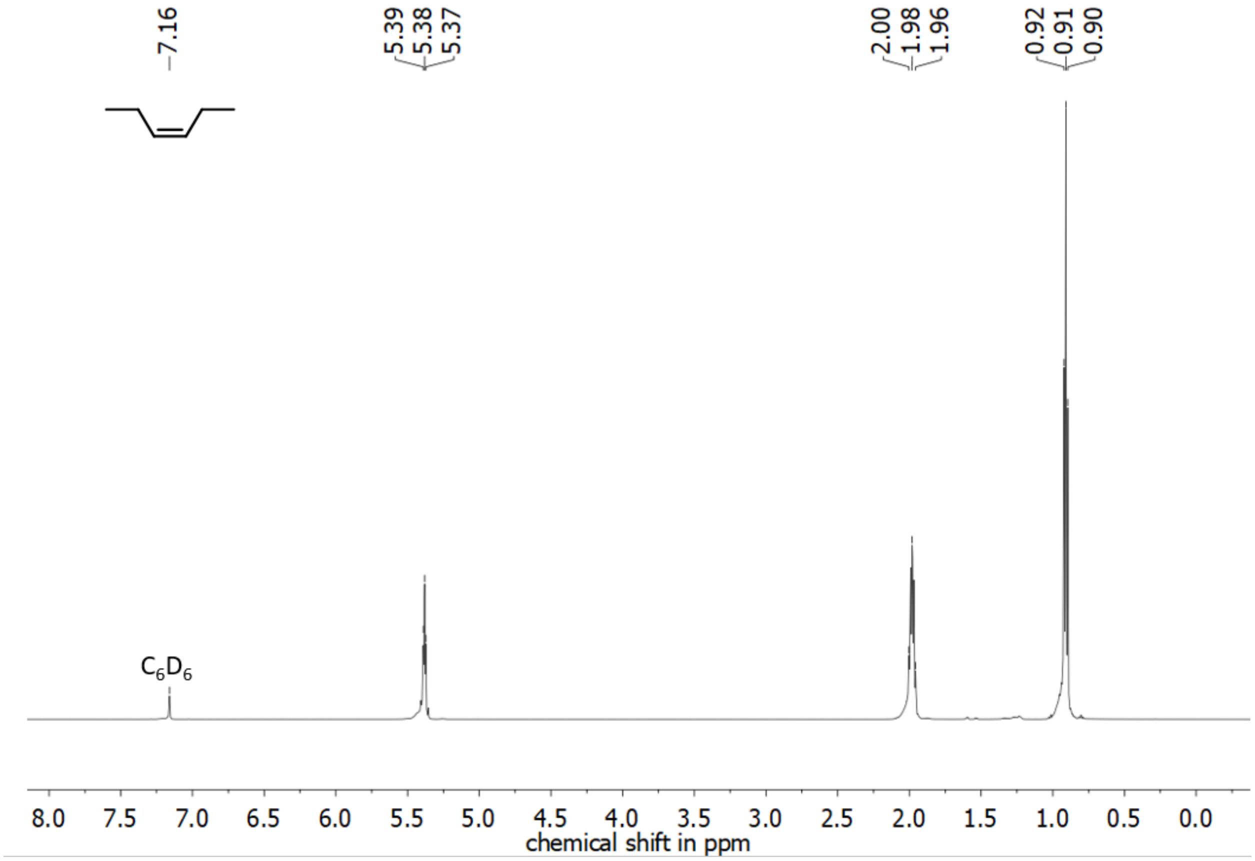


**Figure S9: $^{1}$H NMR spectrum (600 MHz, $C_6D_6$, 25°C) of *cis*-3-hexene.**

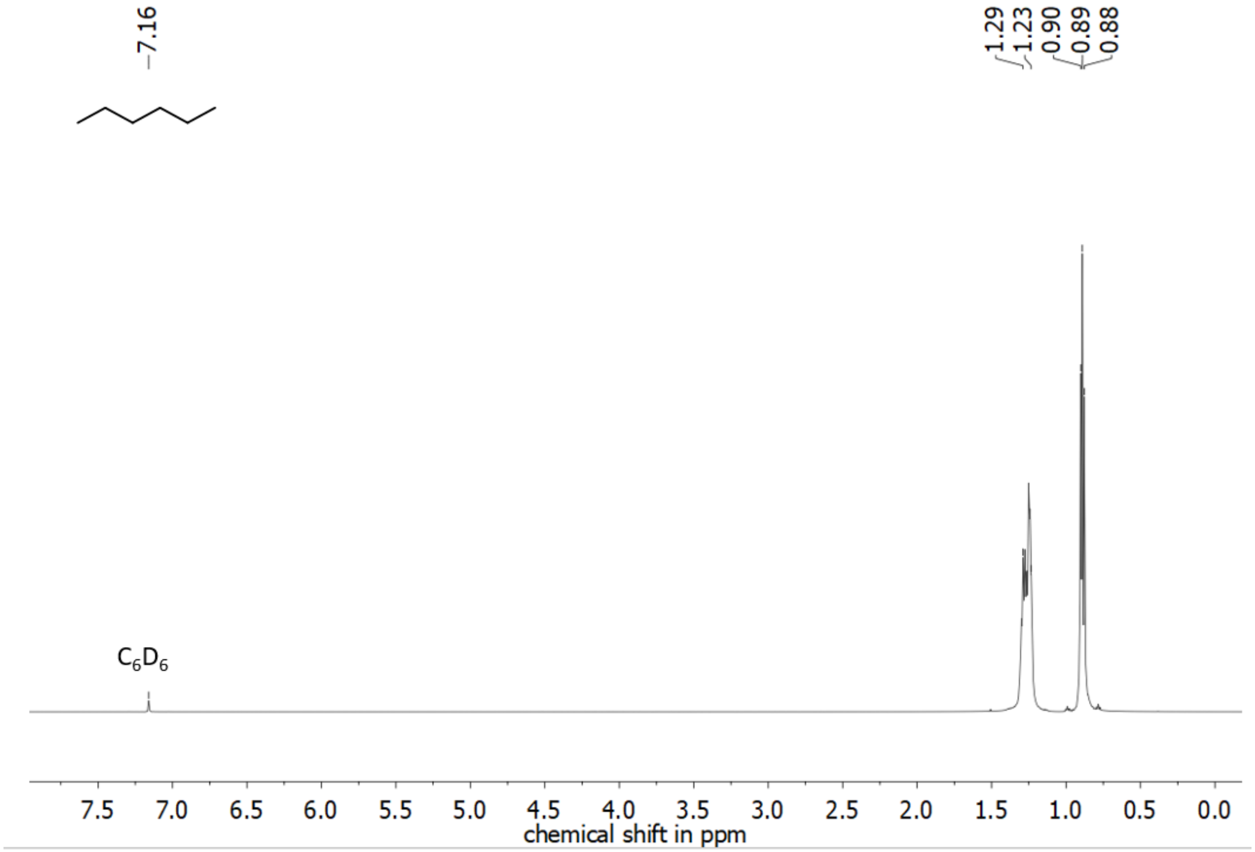


**Figure S10: $^{1}$H NMR spectrum (600 MHz, $C_6D_6$, 25°C) of *cis*-3-hexene after catalytic hydrogenation (neat) using 5 mol% Ba(0) and 12 bars of $H_2$ at 120°C (1 h).**

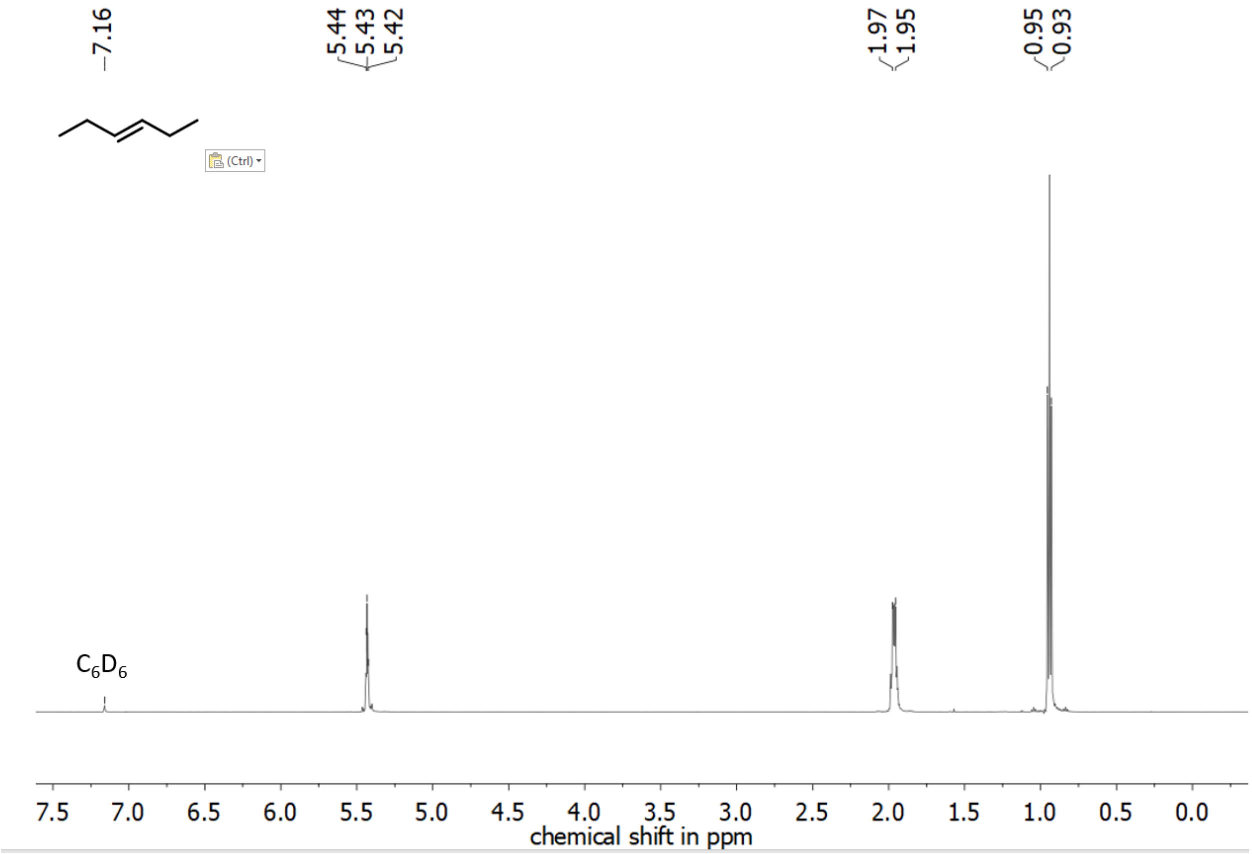


**Figure S11: $^{1}$H NMR spectrum (600 MHz, $C_6D_6$, 25°C) of *trans*-3-hexene.**

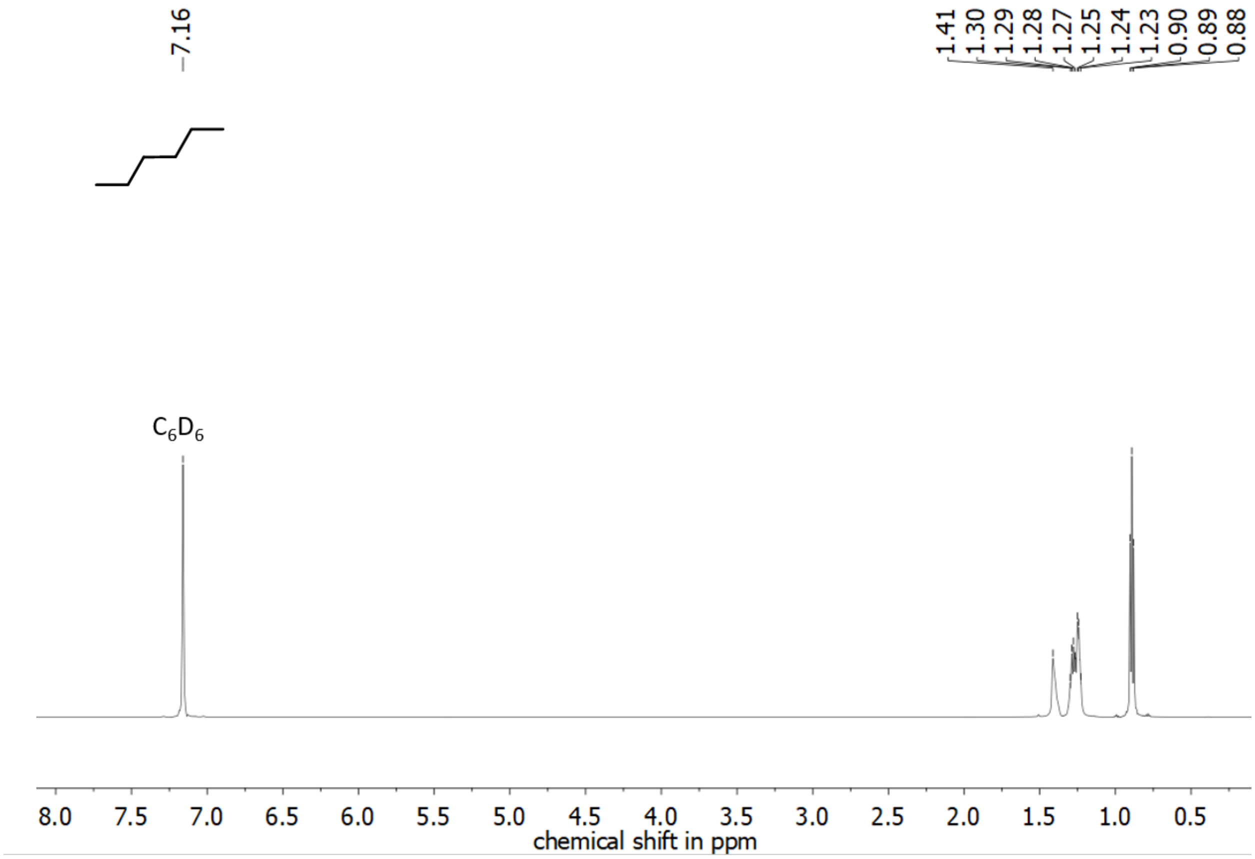


**Figure S12: $^{1}$H NMR spectrum (600 MHz, $C_6D_6$, 25°C) of *trans*-3-hexene after catalytic hydrogenation (neat) using 10 mol% Ba(0) and 20 bars of $H_2$ at 120°C (24 h).**

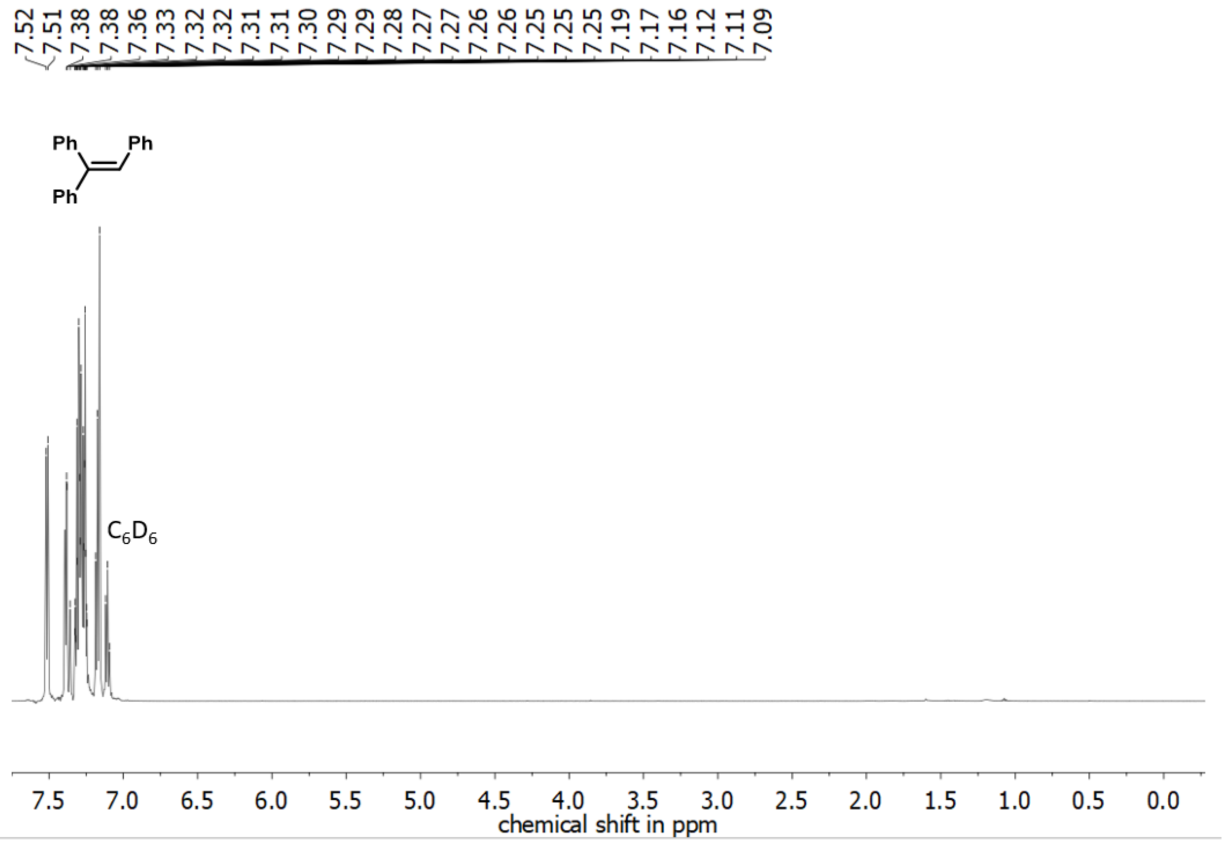


**Figure S13: $^1$H NMR spectrum (600 MHz, $C_6D_6$, 25°C) of triphenylethylene.**

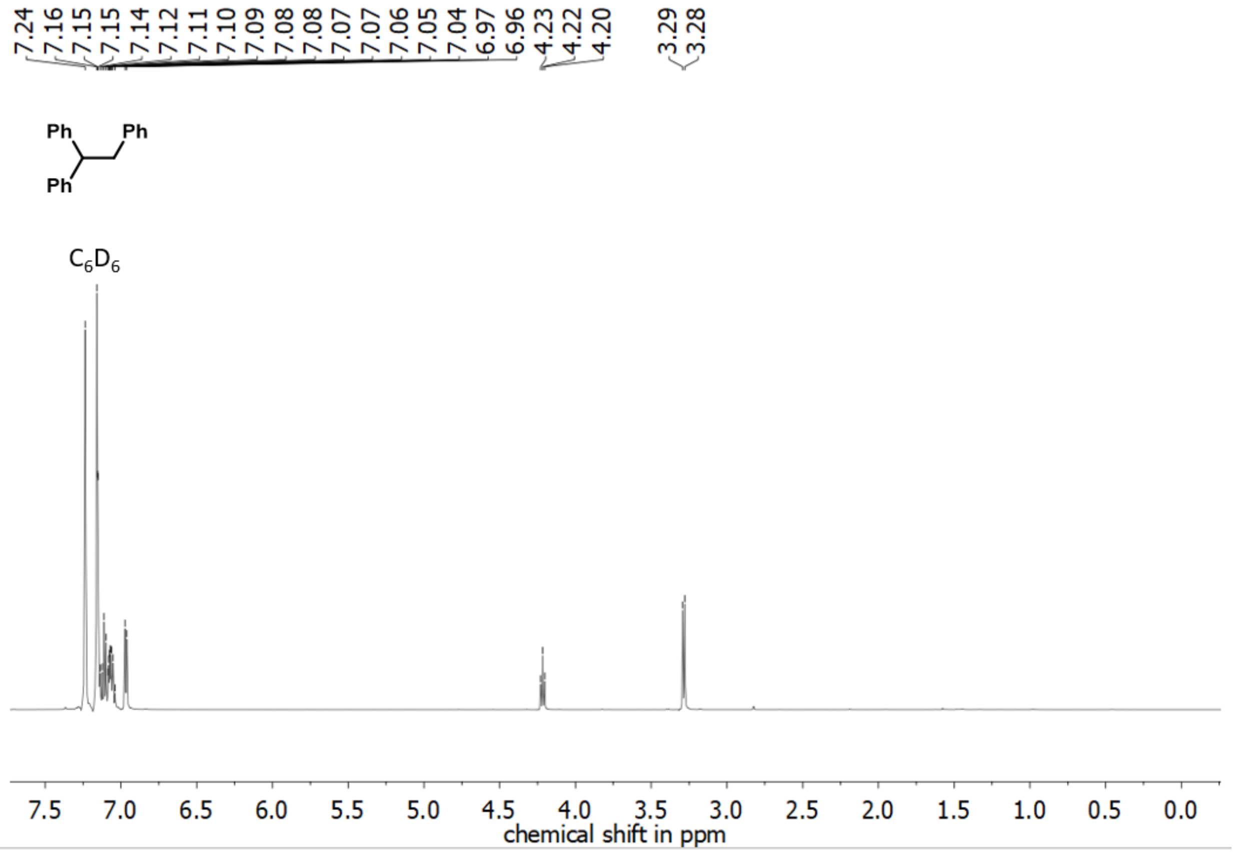


**Figure S14: $^{1}$H NMR spectrum (600 MHz, $C_6D_6$, 25°C) of triphenylethylene after catalytic hydrogenation (1M, $C_6D_6$) using 5 mol% Ba(0) and 20 bars of $H_2$ at 120°C (6 h).**

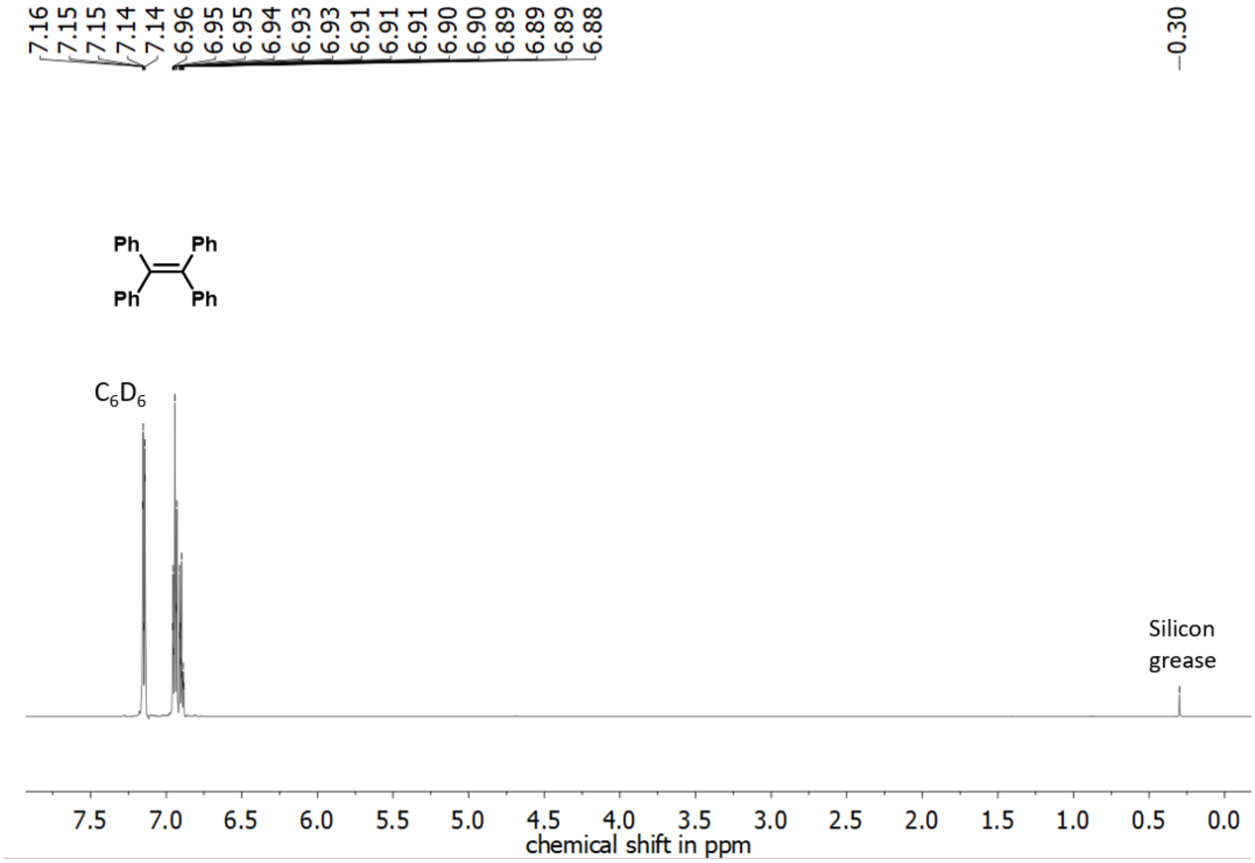


**Figure S15: $^{1}$H NMR spectrum (600 MHz, $C_6D_6$, 25°C) of tetraphenylethylene.**

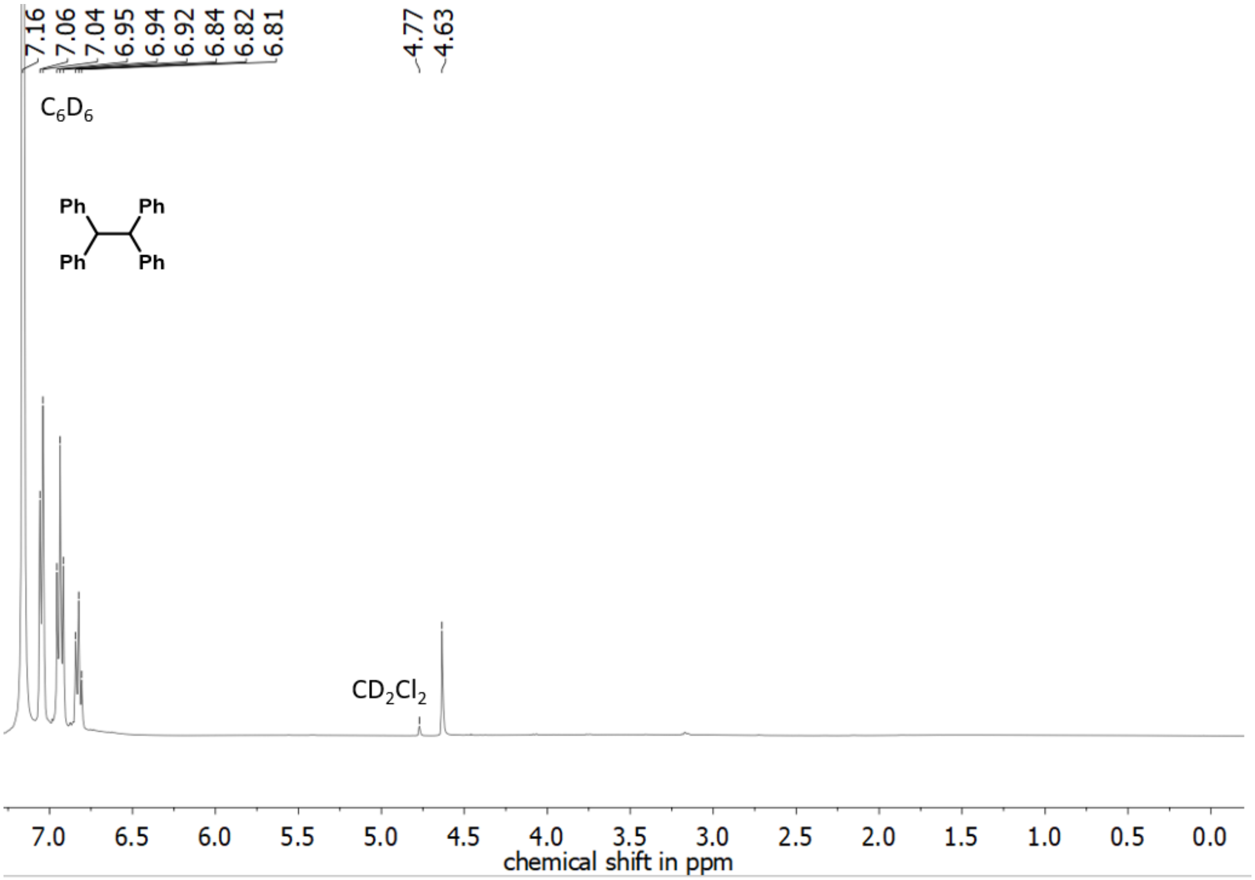


**Figure S16: $^{1}$H NMR spectrum (600 MHz, $C_6D_6$, 25°C) of tetraphenylethylene after catalytic hydrogenation (1M, $C_6D_6$) using 10 mol% Ba(0) and 20 bars of $H_2$ at 150°C (24 h). The sample was dilute with 0.5 mL of $CD_2Cl_2$.**

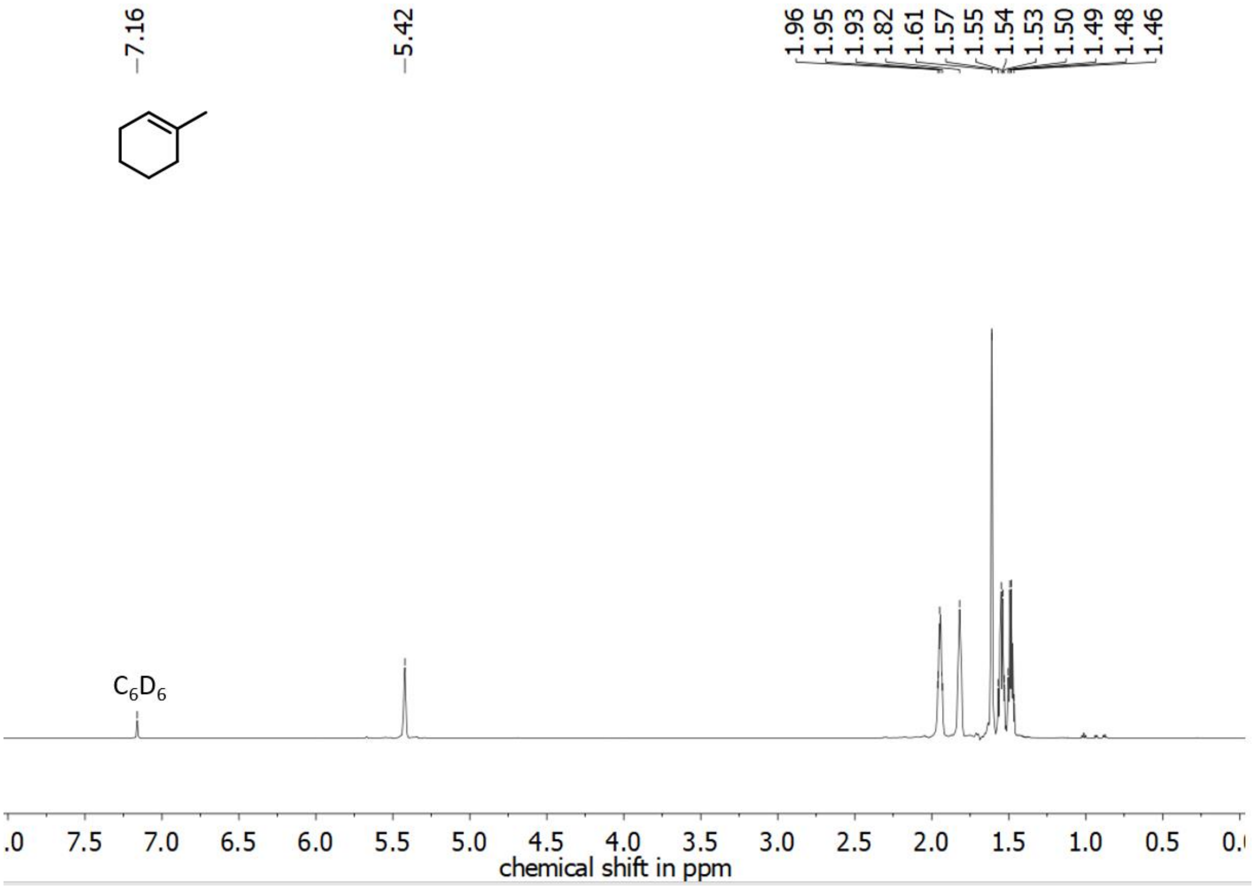


**Figure S17: $^{1}$H NMR spectrum (600 MHz, $C_6D_6$, 25°C) of 1-methyl-1-cyclohexene.**

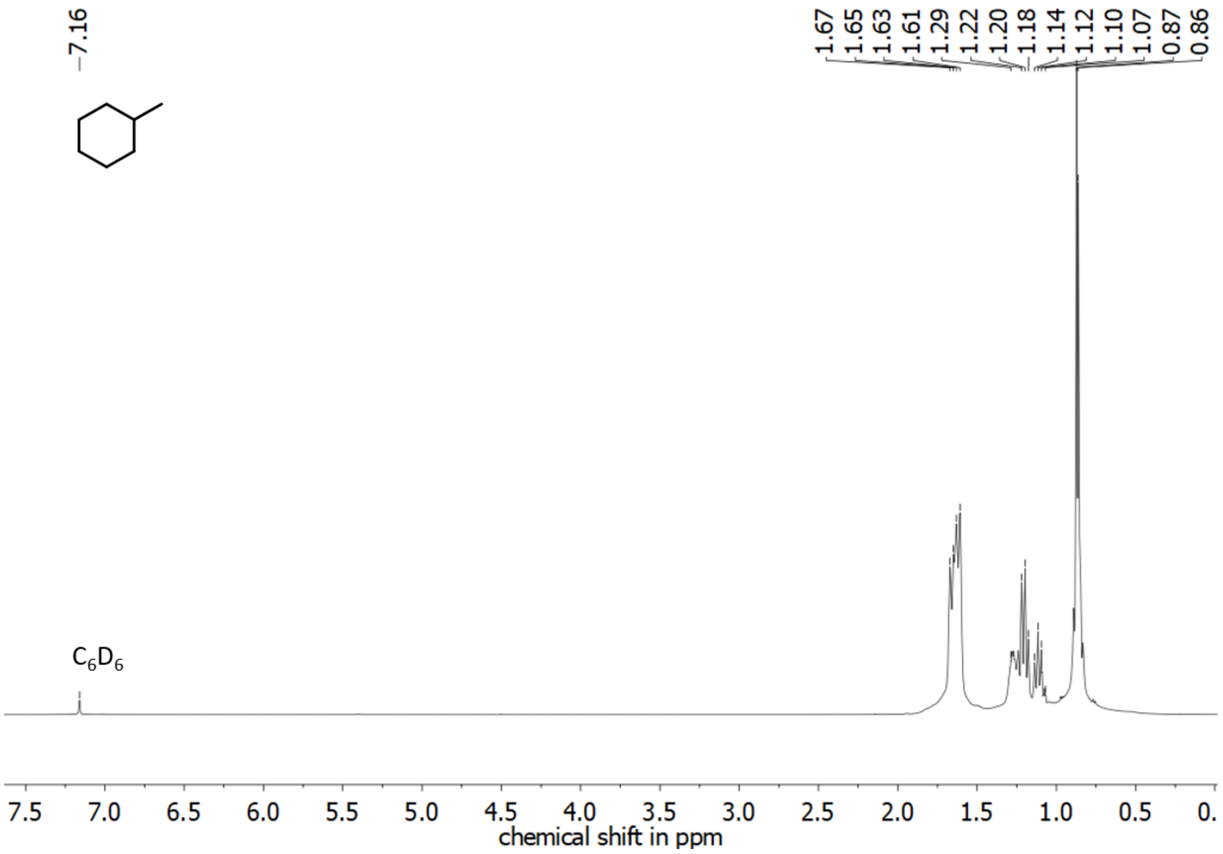


**Figure S18: $^1$H NMR spectrum (600 MHz, $C_6D_6$, 25°C) of 1-methyl-1-cyclohexene after catalytic hydrogenation (neat) using 10 mol% Ba(0) and 20 bars of $H_2$ at 150°C (24 h).**

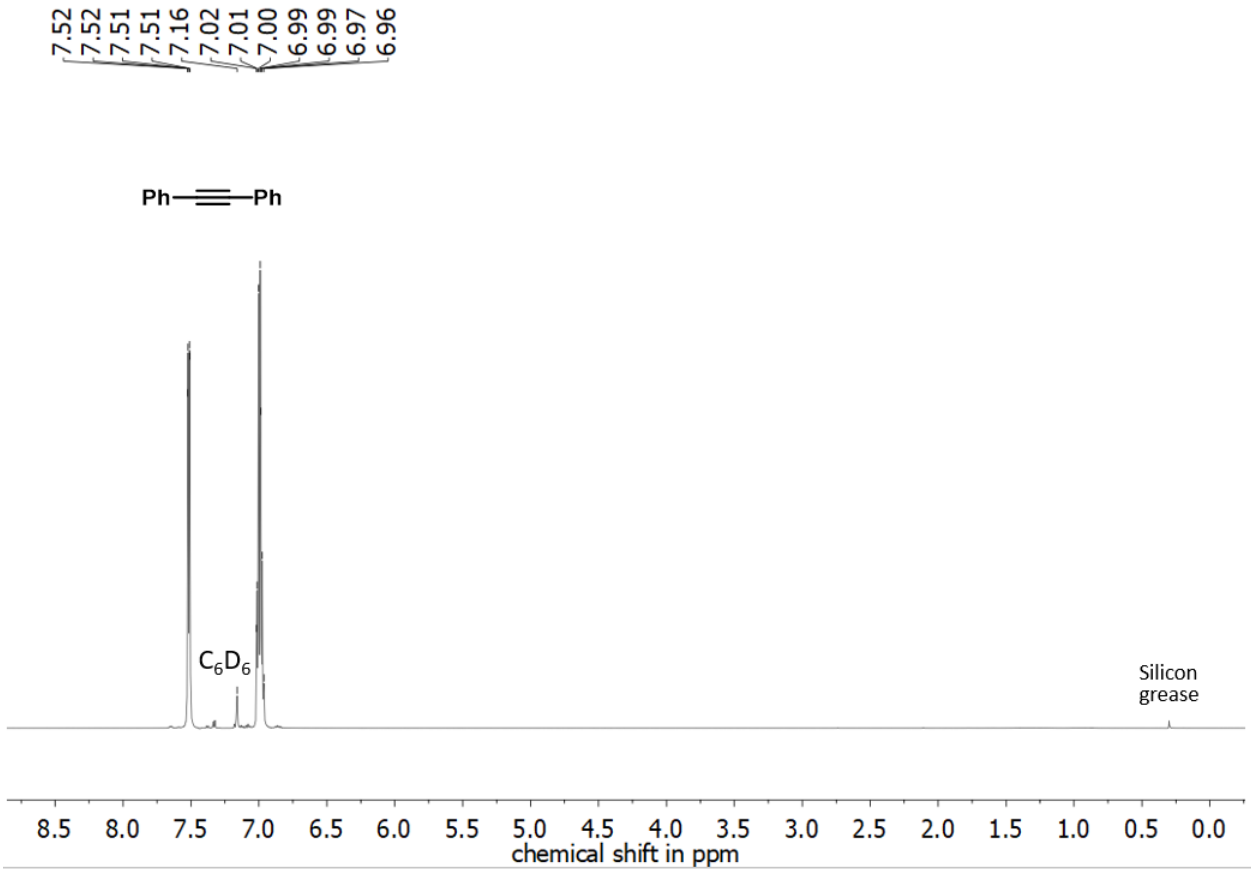


**Figure S19: $^1$H NMR spectrum (600 MHz, $C_6D_6$, 25°C) of diphenylacetylene.**

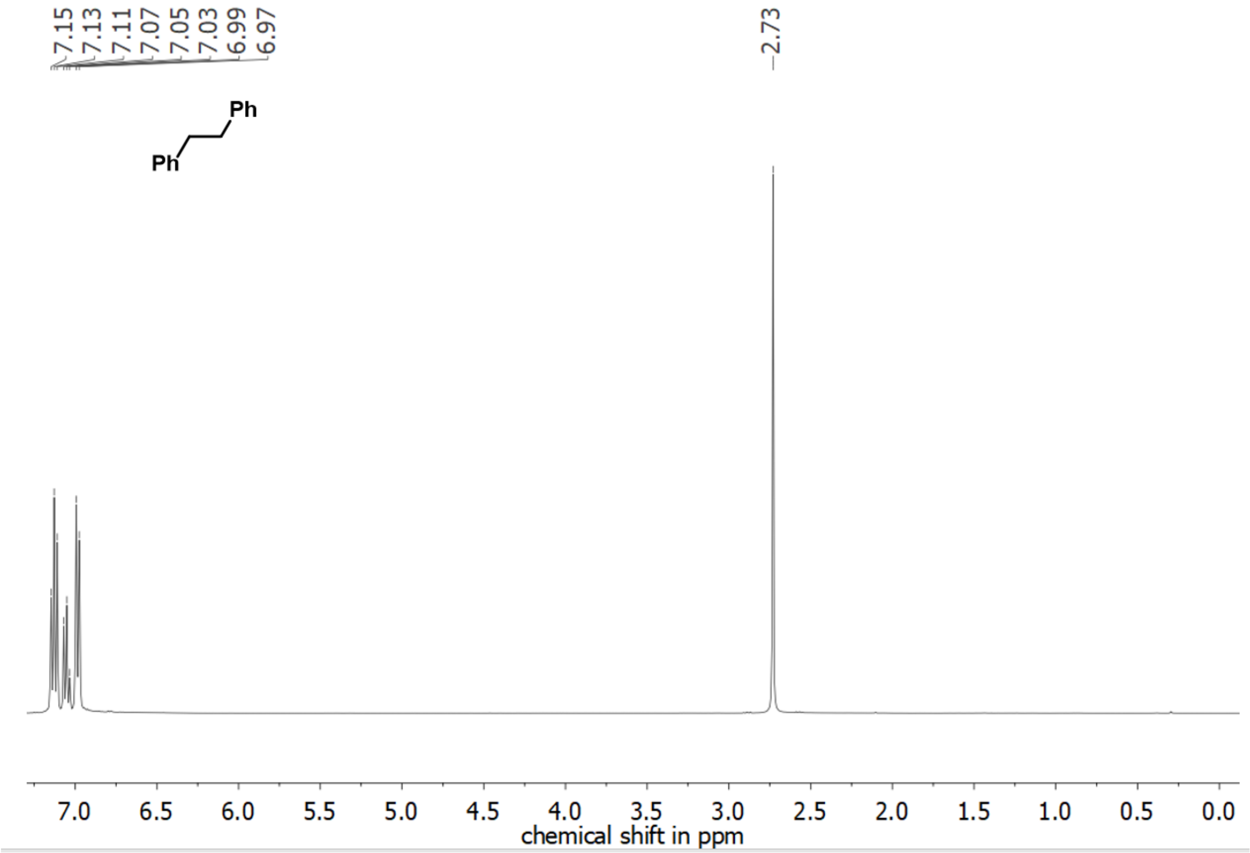


**Figure S20: $^{1}$H NMR spectrum (600 MHz, $C_6D_6$, 25°C) of diphenylacetylene after catalytic hydrogenation (1M, $C_6D_6$) using 5 mol% Ba(0) and 20 bars of $H_2$ at 120°C (6 h).**

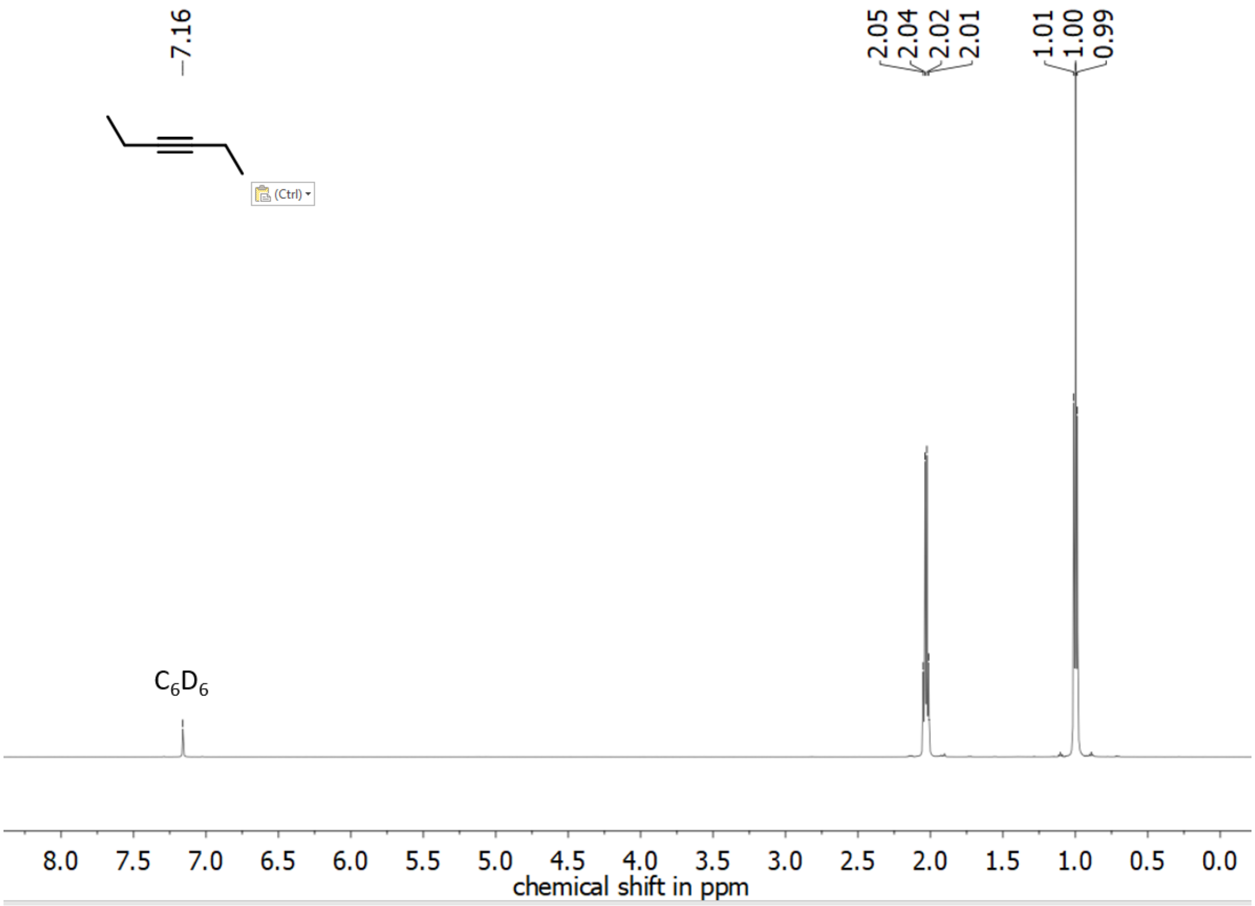


**Figure S21: $^1H$ NMR spectrum (600 MHz, $C_6D_6$, 25°C) of hex-3-yne.**

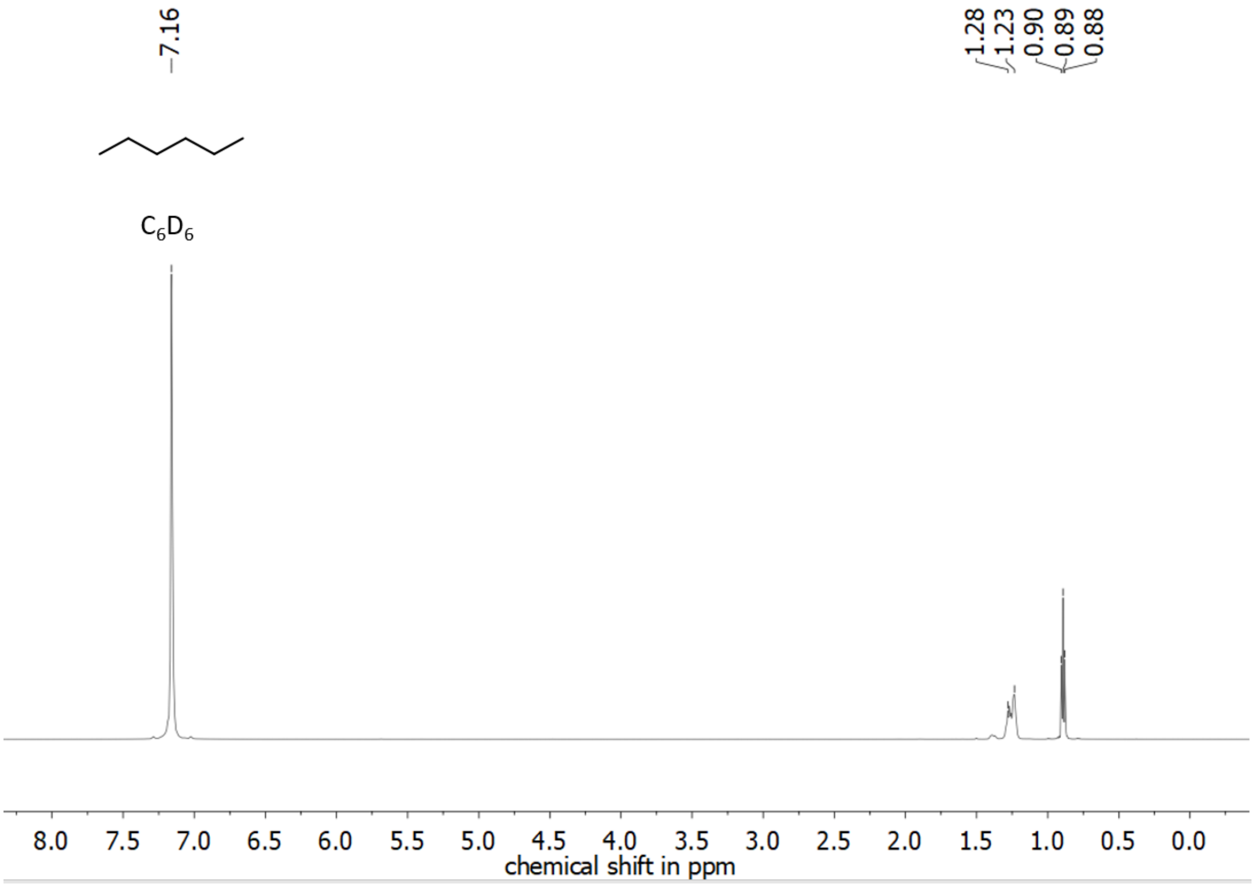


**Figure S22: $^{1}$H NMR spectrum (600 MHz, $C_6D_6$, 25°C) of hex-3-yne after catalytic hydrogenation (neat) using 10 mol% Ba(0) and 20 bars of $H_2$ at 120°C (24 h).**

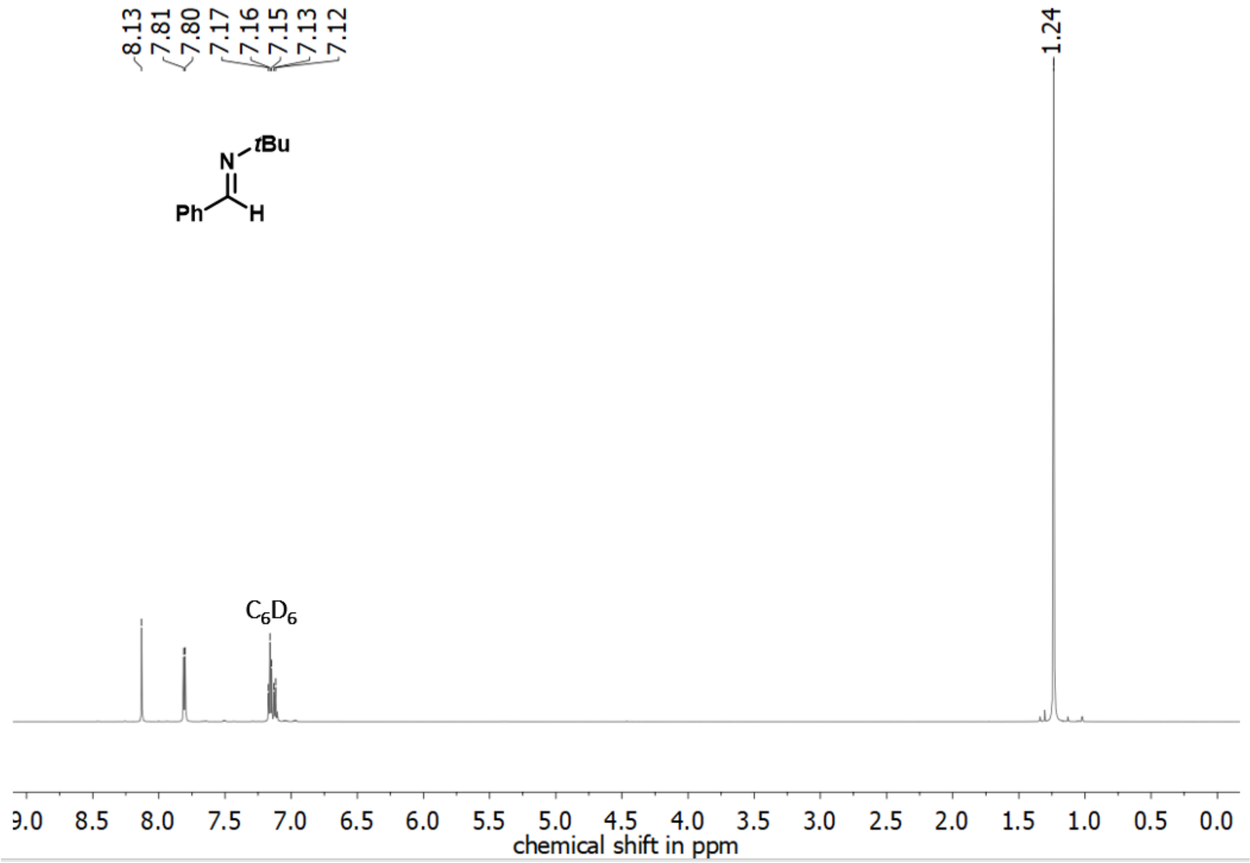


**Figure S23: $^{1}$H NMR spectrum (600 MHz, $C_6D_6$, 25°C) of Ph-*t*Bu-imine.**

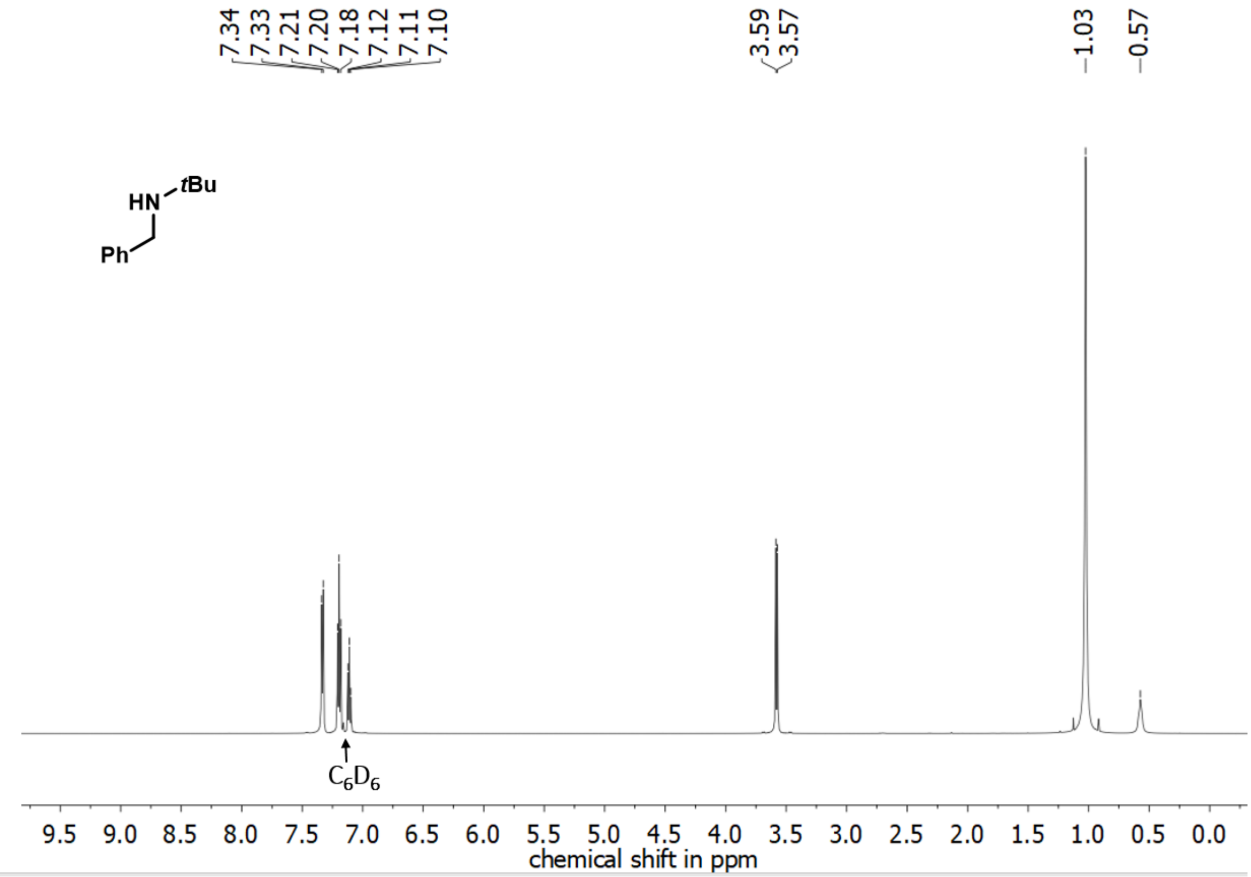


**Figure S24: $^{1}$H NMR spectrum (600 MHz, $C_6D_6$, 25°C) of Ph-*t*Bu-imine after catalytic hydrogenation (neat) using 5 mol% Ba(0) and 12 bars of $H_2$ at 120°C (3.5 h).**

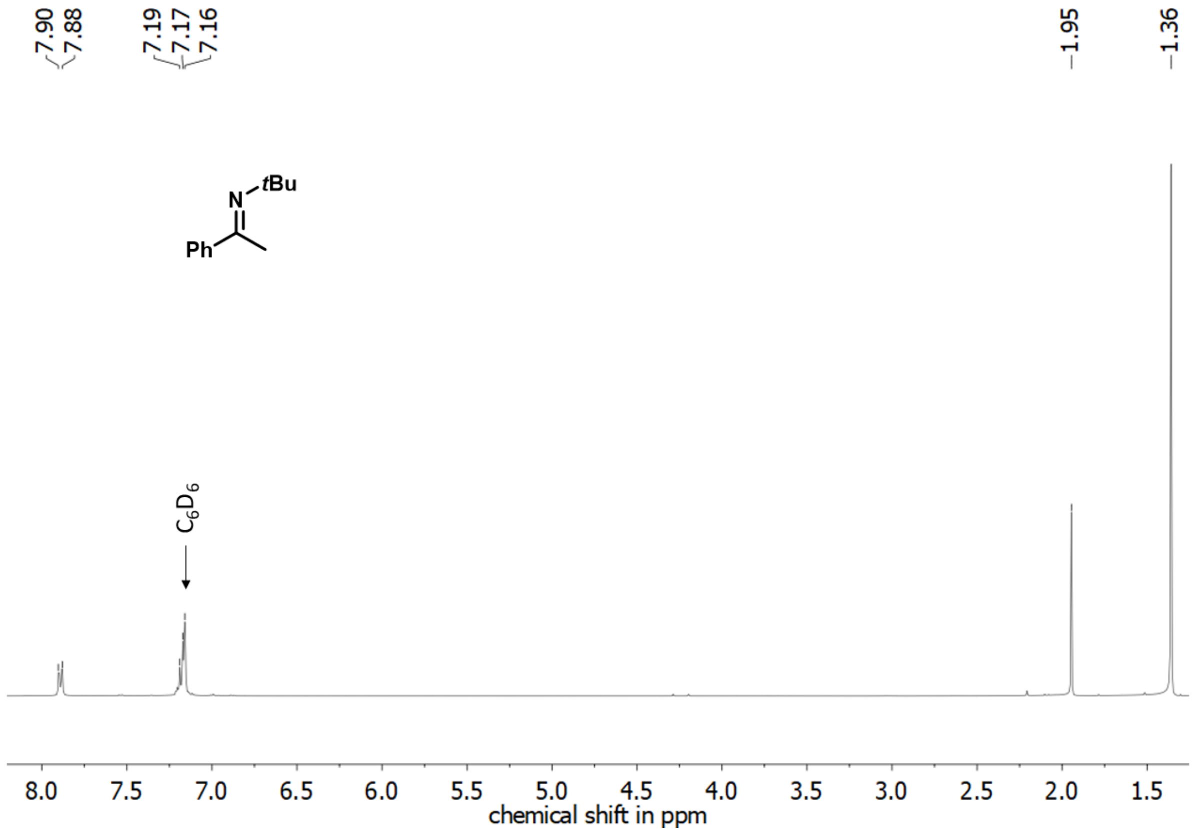


**Figure S25: $^{1}$H NMR spectrum (600 MHz, $C_6D_6$, 25°C) of Ph-Me-*t*Bu-imine.**

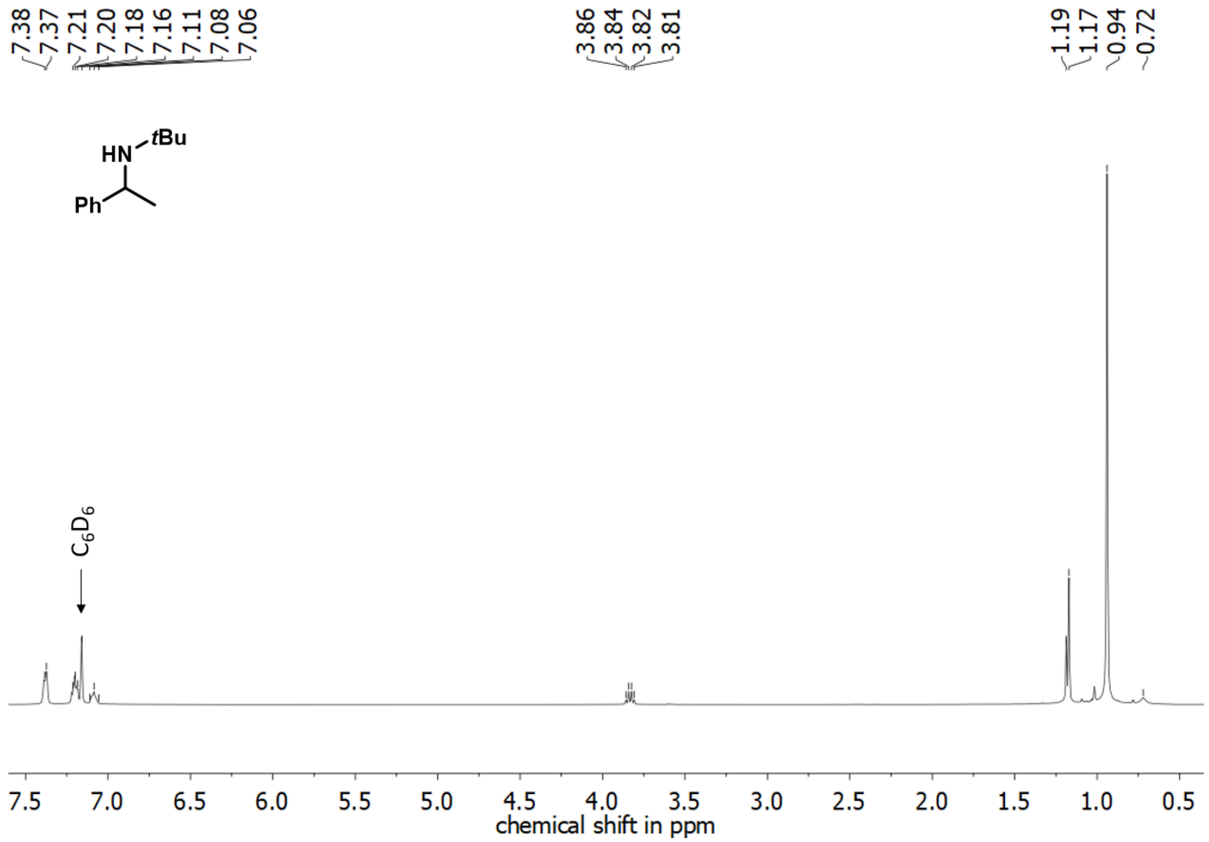


**Figure S26: $^1$H NMR spectrum (600 MHz, $C_6D_6$, 25°C) of Ph-Me-*t*Bu-imine after catalytic hydrogenation (neat) using 10 mol% Ba(0) and 20 bars of $H_2$ at 120°C (24 h).**

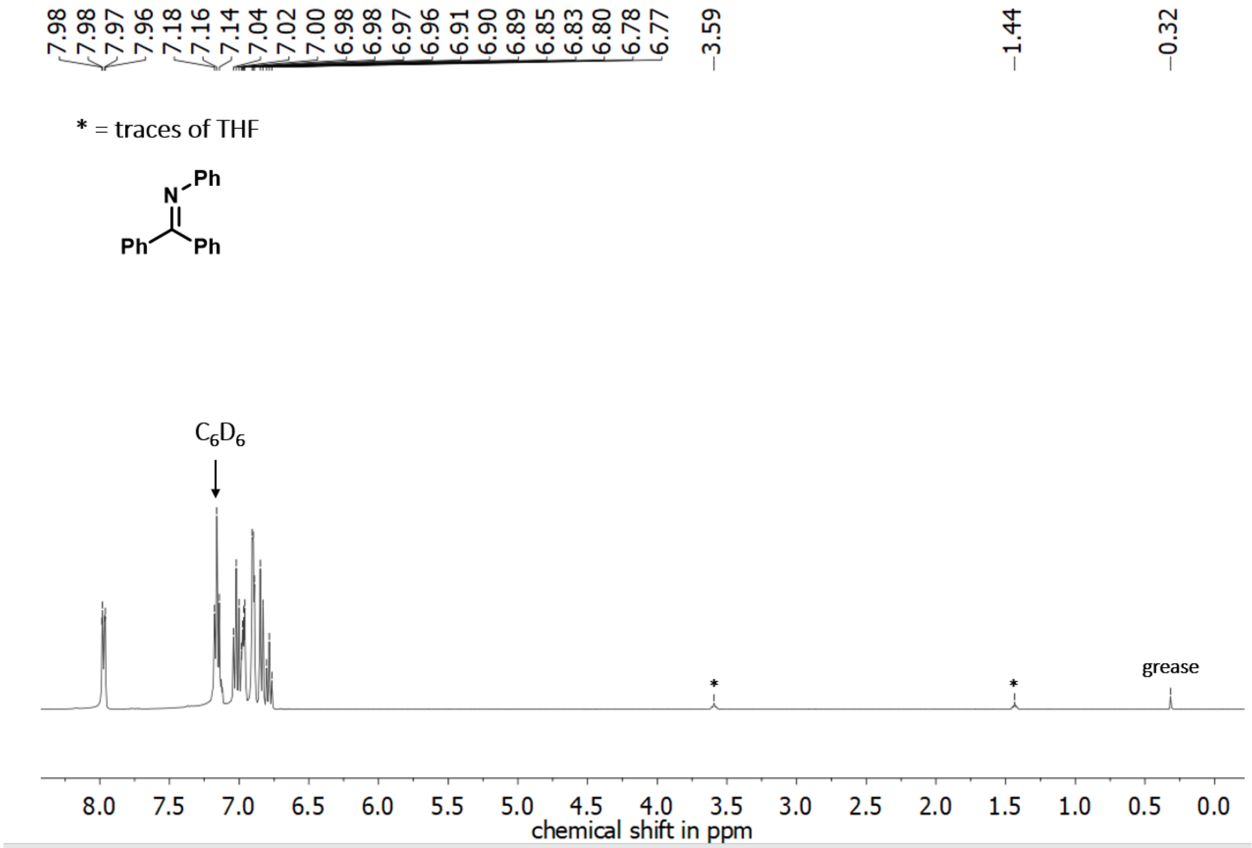


**Figure S27: $^{1}$H NMR spectrum (600 MHz, $C_6D_6$, 25°C) of Ph-Ph-Ph-imine.**

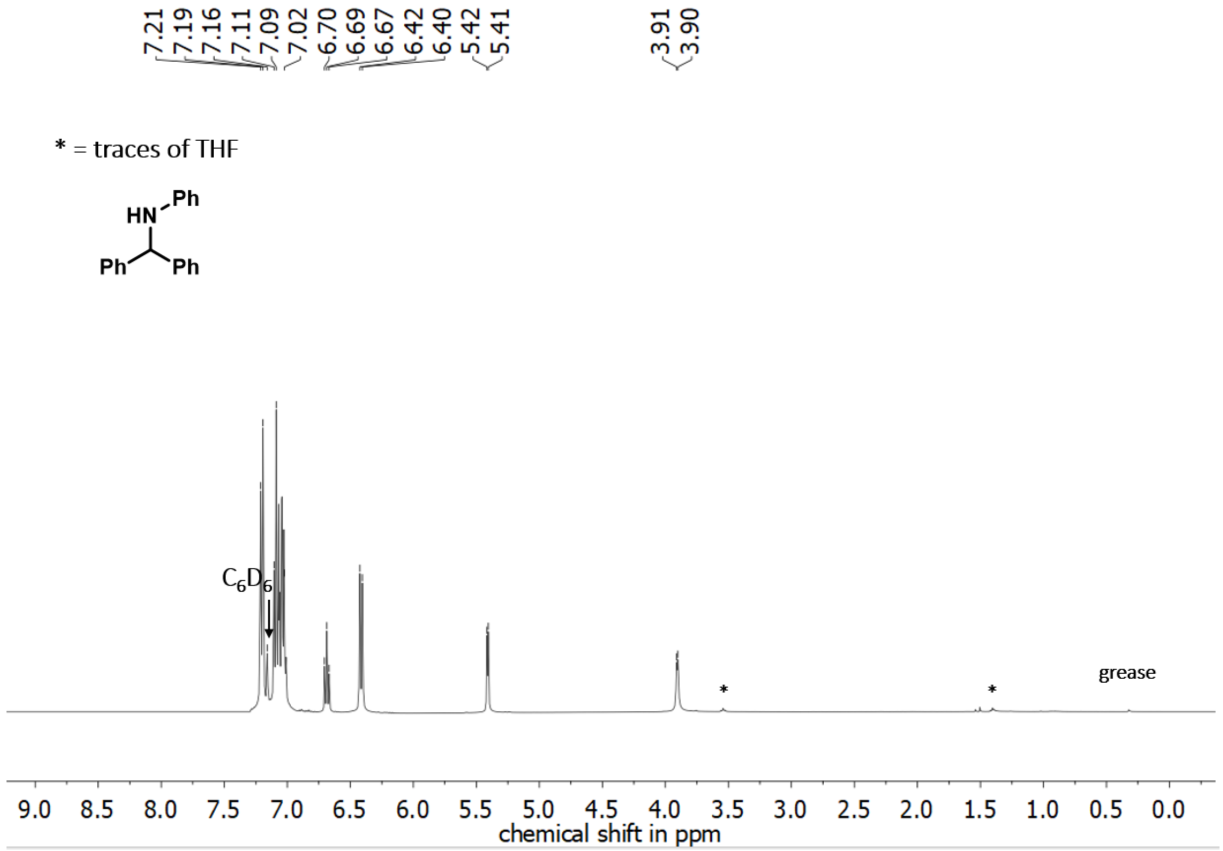


**Figure S28: $^{1}$H NMR spectrum (600 MHz, $C_6D_6$, 25°C) of Ph-Ph-Ph-imine after catalytic hydrogenation (1M, $C_6D_6$) using 5 mol% Ba(0) and 20 bars of $H_2$ at 120°C (8 h).**

## 1.4 Catalytic Hydrogenation of Arenes

### General procedure for the catalytic hydrogenation of arenes

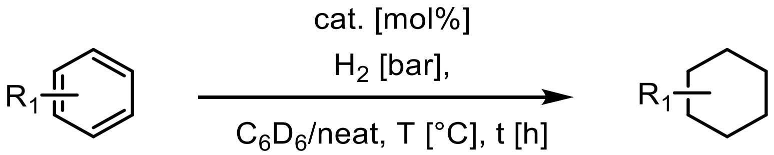


**Figure S29: Catalytic hydrogenation of arenes.**

An oven-dried miniature stainlesssteel autoclave (15mL) containing a magnetic stir bar was charged with the Ba(0) pre-catalyst (6.9 mg, 0.05 mmol, 5 mol% or 13.7 mg, 0.1 mmol, 10 mol%) and the arene of interest in an $N_2$-filled glovebox. The reactions were performed neat, or as $C_6D_6$ solution (1M, 1 mL). In case of pyridine, $C_6D_{12}$ was chosen as solvent to minimize corrosion of the reactor O-ring sealing. The tightly sealed pressure vessel was transferred out of the glovebox and connected to a $H_2$ gas manifold. The supply line was evacuated for 1 minute and subsequently purged with dry gas. This process was repeated for three times using $N_2$ and for three further times using $H_2$ to ensure the exclusion of air and moisture. The reactor was then pressurized with $H_2$ (6 bar to 50 bar) and heated to the desired temperature (120°C to 150°C) in a pre-heated aluminum metal block. After stirring the reaction mixture for the indicated amount of time, the autoclave was cooled to room temperature in a water bath and subsequently vented. The reaction mixture was filtered through a glass microfiber filter in a Pasteur pipette and the crude filtrate was analyzed by $^1$H NMR spectroscopy without additional purification. The products were identified by comparison of their $^1$H NMR spectra with the corresponding data reported in literature.[S4,S5] The conversion of substrates was estimated via integration of characteristic $^1$H NMR resonances of the product and their residual aromatic counter parts. When using $C_6D_6$ as solvent, a significant degree of deuterium incorporation in the hydrogenated products, resulting from H/D isotope exchange by solvent activation, was observed. Therefore in case of insufficient peak separation product composition was additionally analyzed by GC/MS, via integration of the peaks corresponding to the hydrocarbon products against their unsaturated

analogues. Reactions times for essentially full conversion were optimized in 30 min intervals.

**Table S2.** Catalytic arene hydrogenation; reactions performed in $C_6D_6$ (1 M), if not stated otherwise.

| Entry | Substrate | mol % | $H_2$ [bar] | T [°C] | t [h] | Product(s) | Conv. [%] |
|---|---|---|---|---|---|---|---|
| **1** | 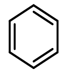 | 10 | 50 | 150 | 6d [3d]# | 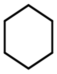 | 99[a] [18]# |
| **2** | 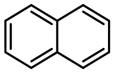 | 10 | 20 | 120 | 3.5 [2]* | 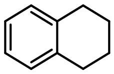 | 99 [99]* |
| **3** | 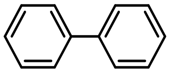 | 5 | 20 | 120 | 12 [10]* | 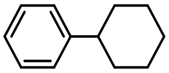 | 99 [99]* |
| **4** | 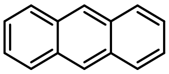 | 5 | 12 | 120 | 24 [2.5]* | 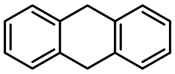 / 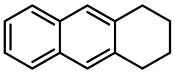 | <5% [94/2]* |
| **5** | 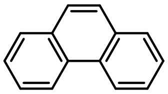 | 10 | 50 | 150 | 24 [48]* | 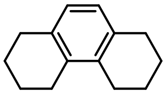 / 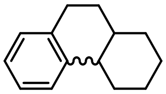 | 31 [49]* / 69[b] [51]* |
| **6** | 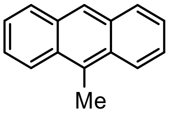 | 5 | 20 | 120 | 25 [24]* | 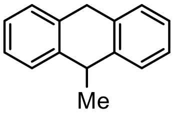 / 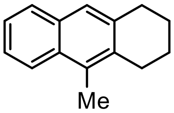 | 86 [84]* / 13 [15]* |
| **7** | 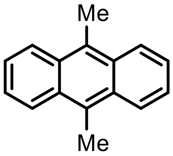 | 10 | 50 | 120 | 24 | 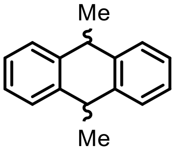 / 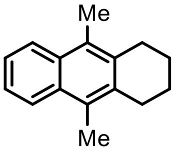 | 85[b] / 12[c] |
| **8** | 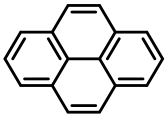 | 10 | 50 | 150 | 48 [24]* | 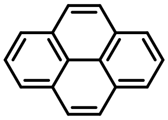 / 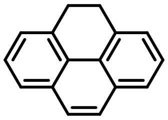 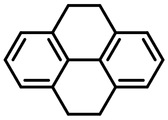 / 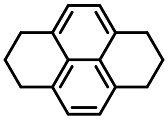 | 2 [94]* / 34 [6]* 43 [0]* / 21 [0]* |
| **9** | 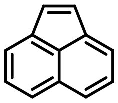 | 10 | 50 | 150 | 24 [24]* | 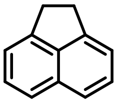 | 50 [0]* |
| **10** | 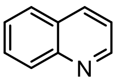 | 10 | 20 | 135 | 24 | 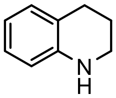 | 99 |
| **11** | 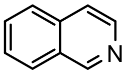 | 10 | 50 | 150 | 65 | 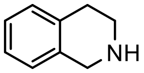 | 99 |
| **12** | 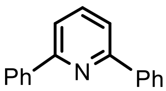 | 10 | 50 | 150 | 24 | 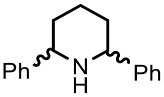 | 99[b] |

| | | | | | | | |
|---|---|---|---|---|---|---|---|
| **13** | 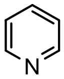 | 10 | 50 | 150 | 89 | 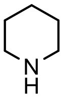 | 99[d] |

[a] = reactions performed neat; [b] = mixture of *cis* and *trans* isomers; [c] = + 3% octahydro-dimethyl-anthracene; [d] = 1M in $C_6D_{12}$; # = 2.5 mol% Ba[N(Si*i*Pr$_3$)$_2$]$_2$, 50 Bar $H_2$, 140°C; * = 10 mol% Ba[N(Si*i*Pr$_3$)$_2$]$_2$, 6 Bar $H_2$, 120°C, 1M in $C_6D_6$.

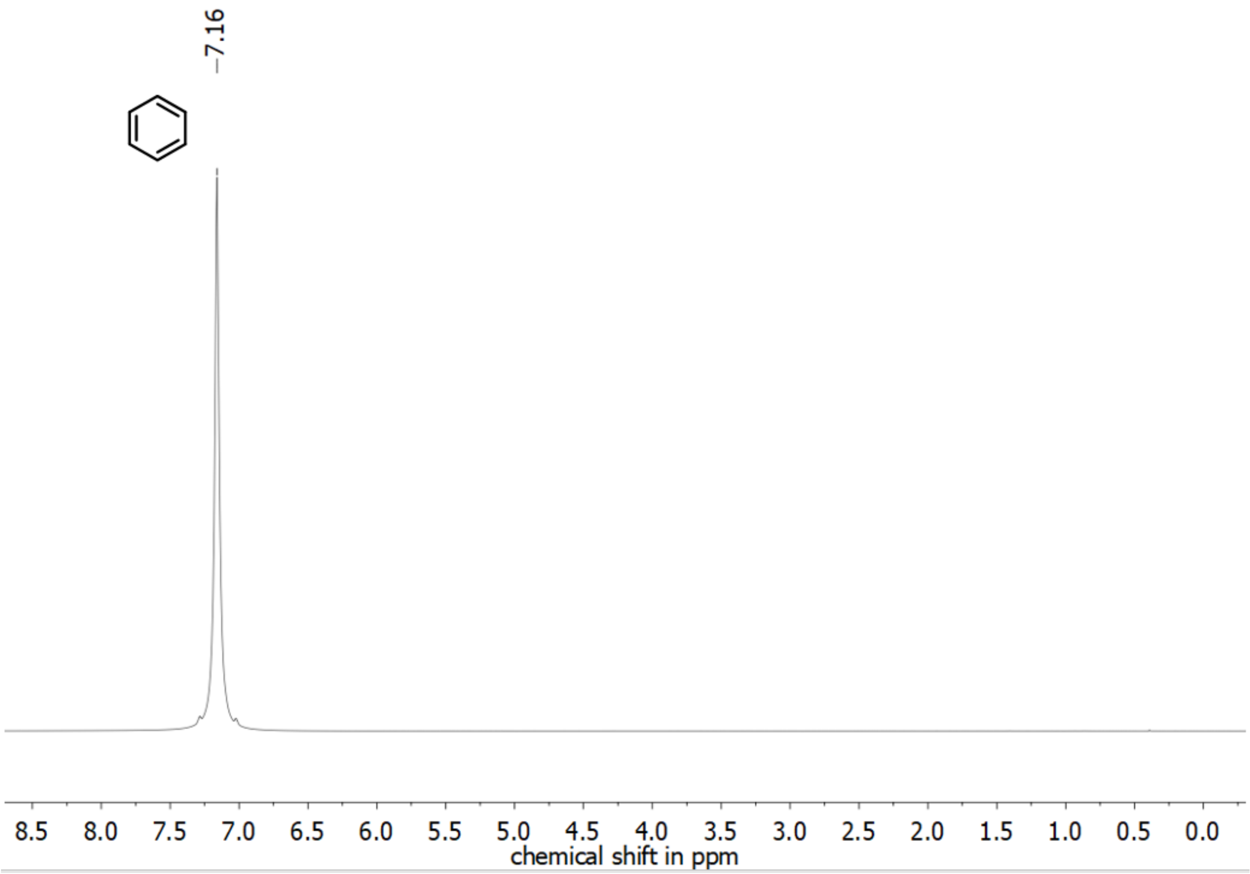


**Figure S30: $^1$H NMR spectrum (600 MHz, no deuterated solvent, 25°C) of benzene.**

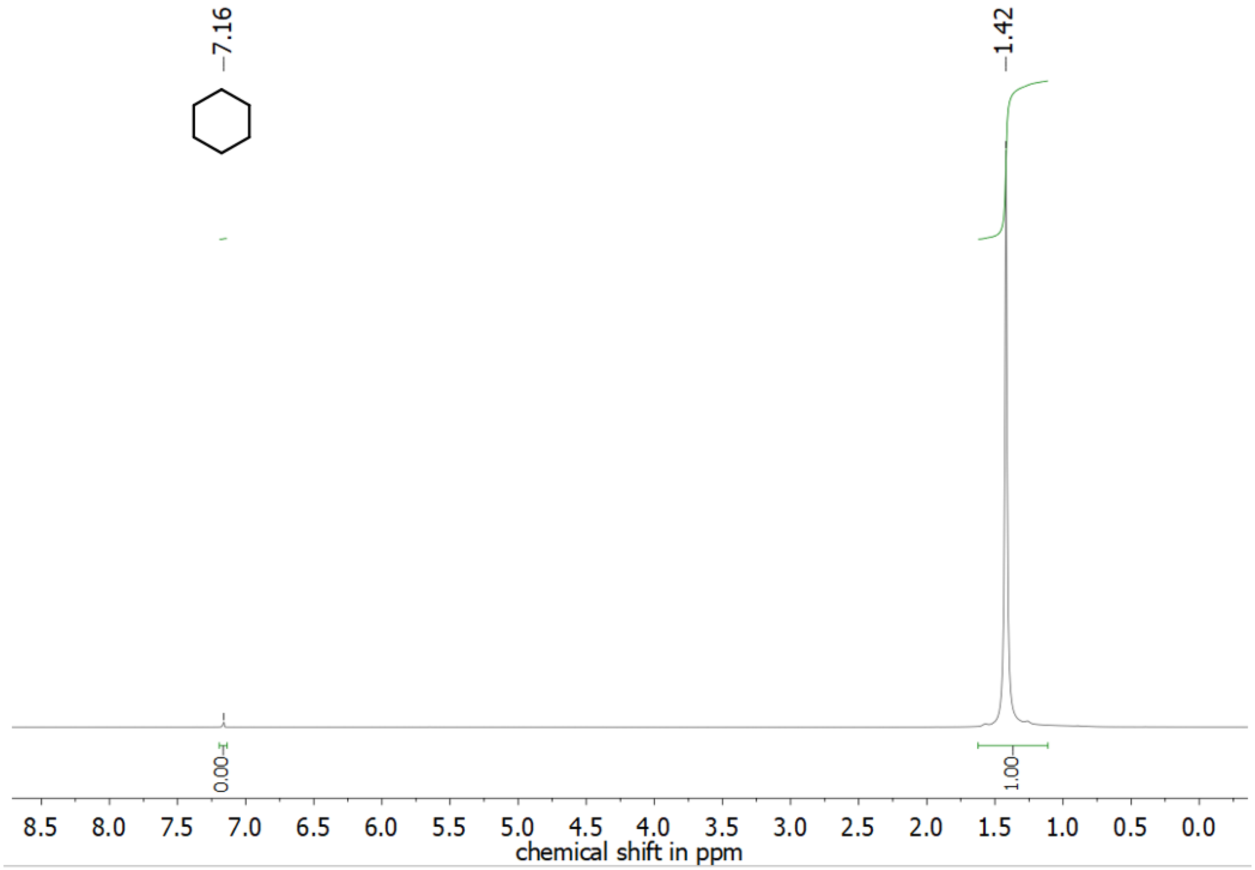


**Figure S31: $^{1}$H NMR spectrum (600 MHz, no deuterated solvent, 25°C) of benzene after catalytic hydrogenation (neat) using 10 mol% Ba(0) and 50 bars of $H_2$ at 150°C (6 days).**

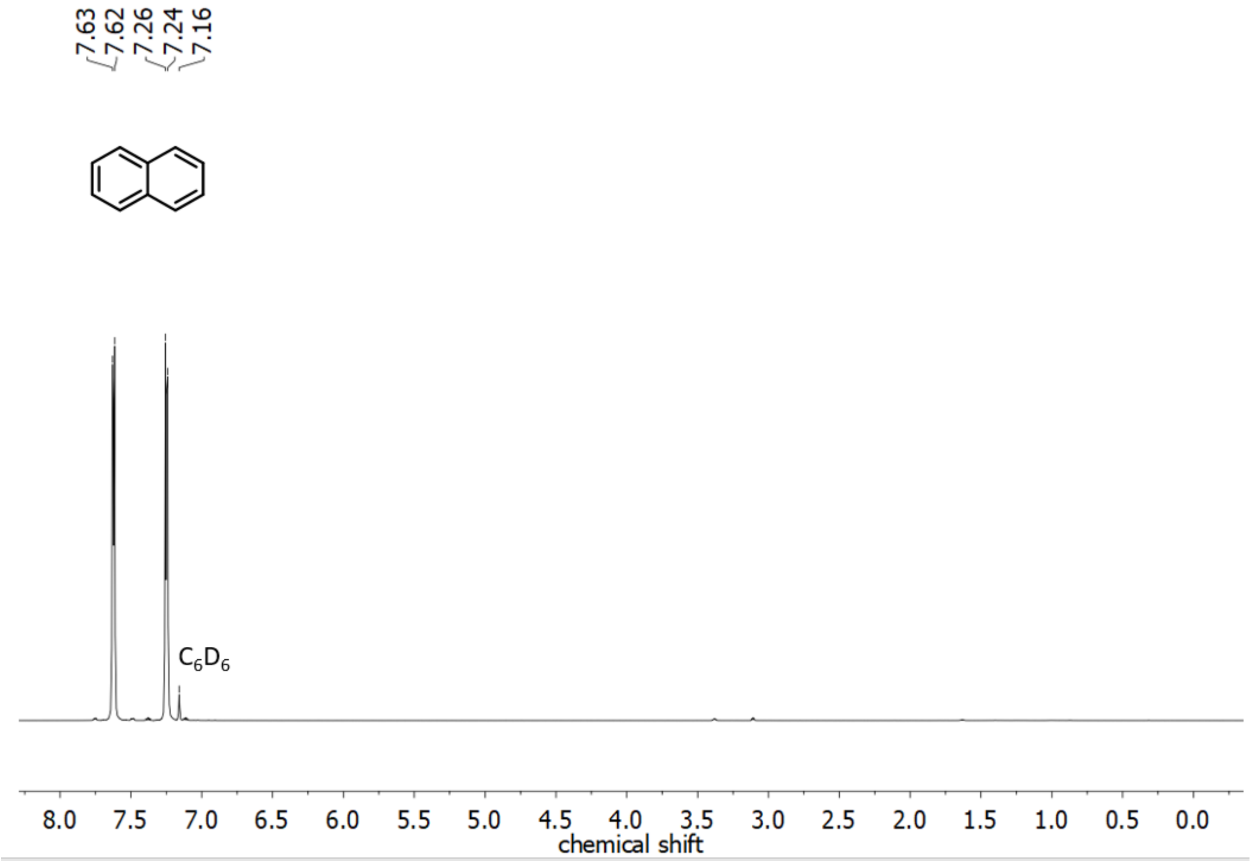


**Figure S32: $^{1}$H NMR spectrum (600 MHz, $C_6D_6$, 25°C) of naphthalene.**

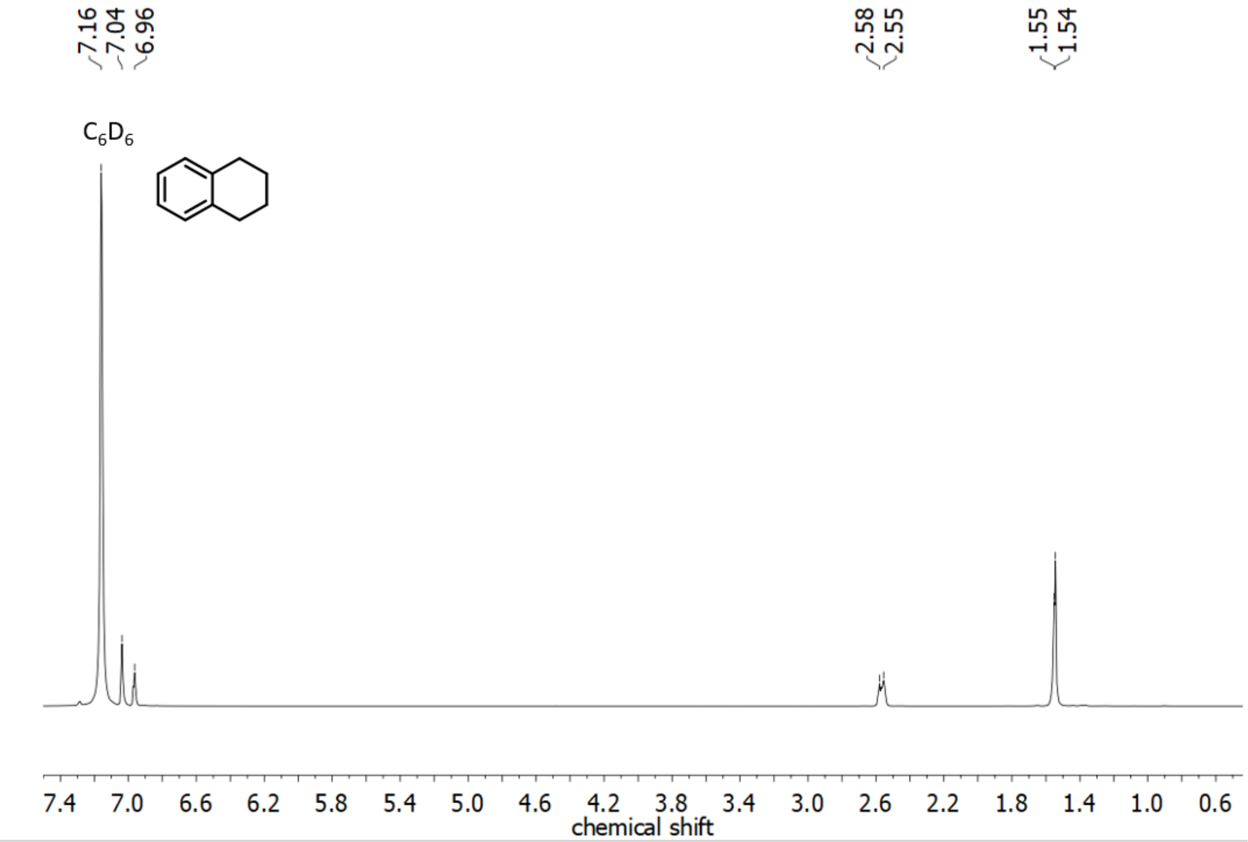


**Figure S33: $^1$H NMR spectrum (600 MHz, $C_6D_6$, 25°C) of naphthalene after catalytic hydrogenation (1M, $C_6D_6$) using 10 mol% Ba(0) and 20 bars of $H_2$ at 120°C (3.5 h). The spectrum indicates deuteration in benzylic position (2.55-2.58 ppm) due to solvent activation.**

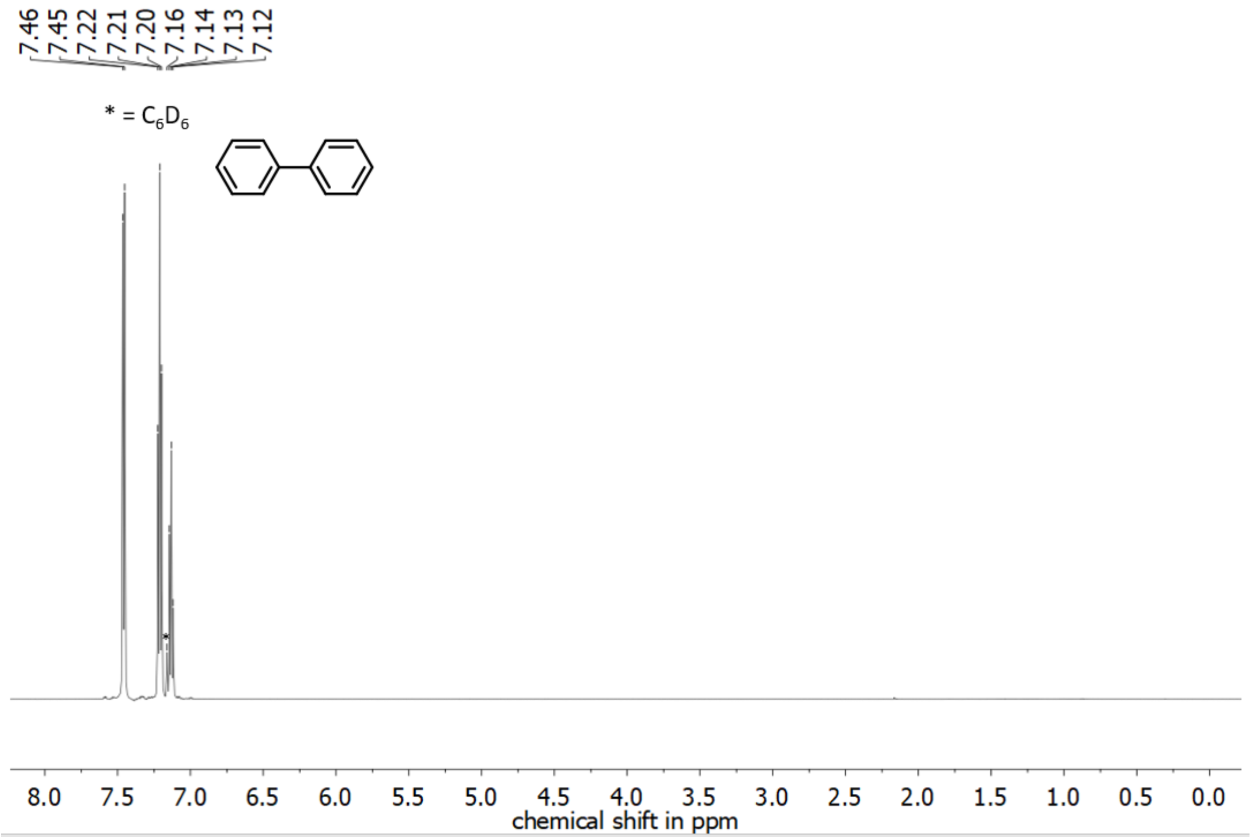


**Figure S34: $^{1}$H NMR spectrum (600 MHz, $C_6D_6$, 25°C) of biphenyl.**

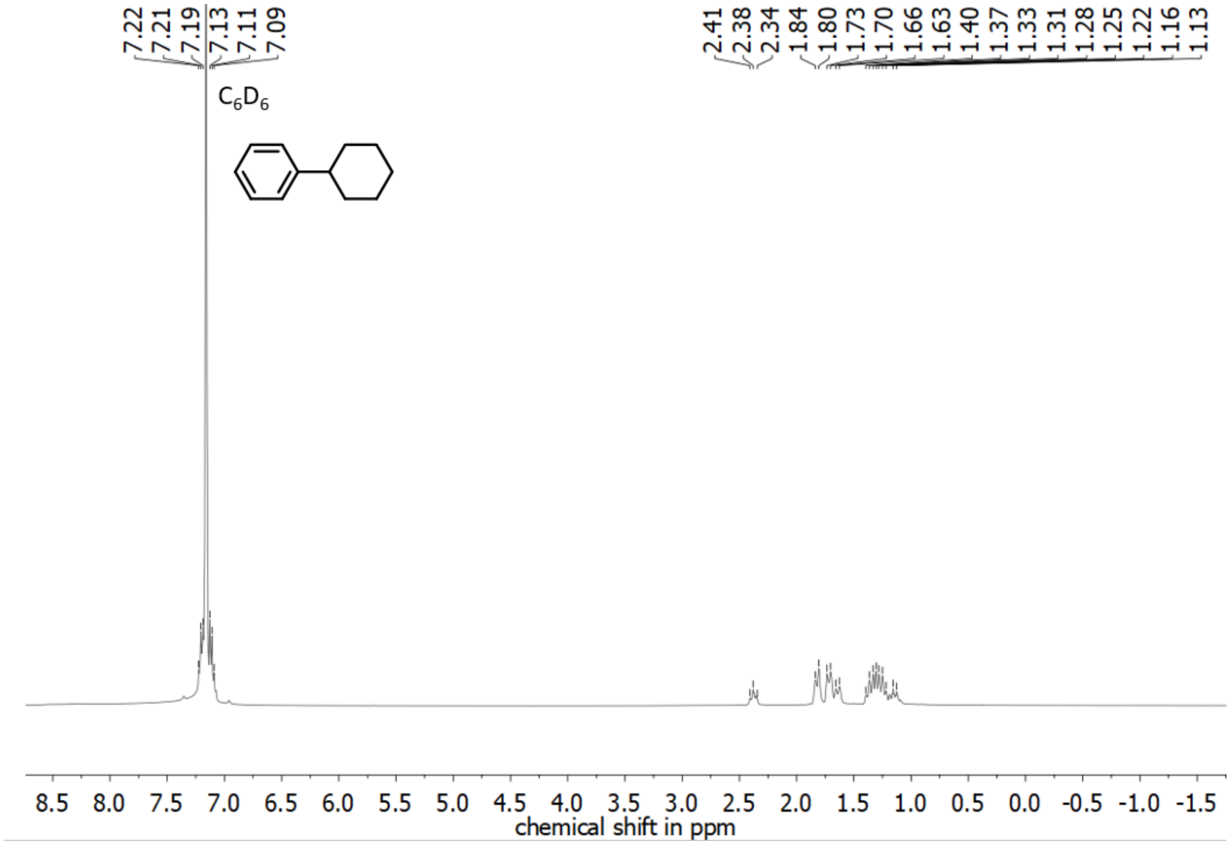


**Figure S35: $^1$H NMR spectrum (600 MHz, $C_6D_6$, 25°C) of biphenyl after catalytic hydrogenation (1M, $C_6D_6$) using 5 mol% Ba(0) and 20 bars of $H_2$ at 120°C (12 h).**

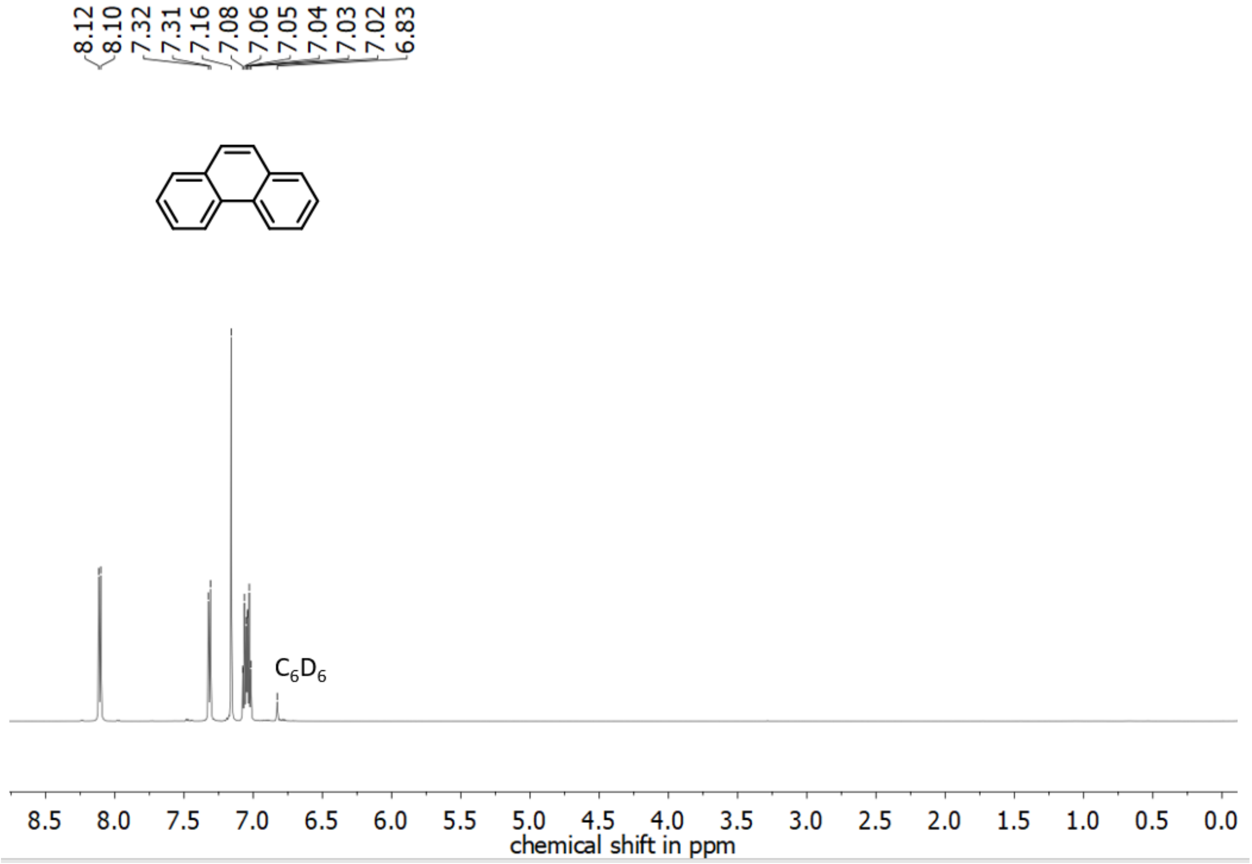


**Figure S36: $^{1}$H NMR spectrum (600 MHz, $C_6D_6$, 25°C) of phenanthrene.**

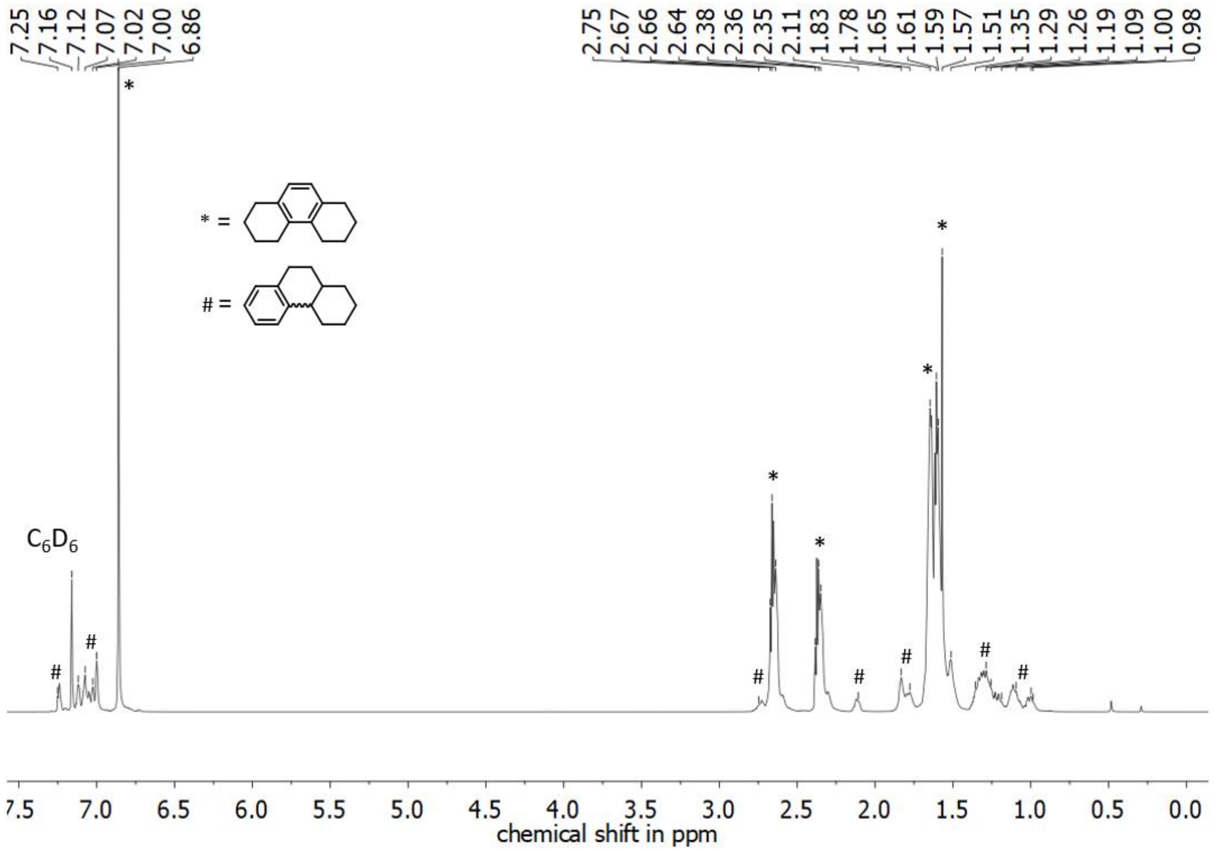


**Figure S37: $^{1}H$ NMR spectrum (600 MHz, $C_6D_6$, 25°C) of phenanthrene after catalytic hydrogenation (1M, $C_6D_6$) using 10 mol% Ba(0) and 50 bars of $H_2$ at 150°C (24 h). 1,2,3,4,4a,9,10,10a-octahydrophenanthrene is obtained as a mixture of *cis*- and *trans*-isomers. Due to HD-exchange between $C_6D_6$ and $H_2$ during arene hydrogenation, the solvent was removed under reduced pressure. The sample was redissolved in fresh $C_6D_6$ to provide a better peak separation in the aromatic region.**

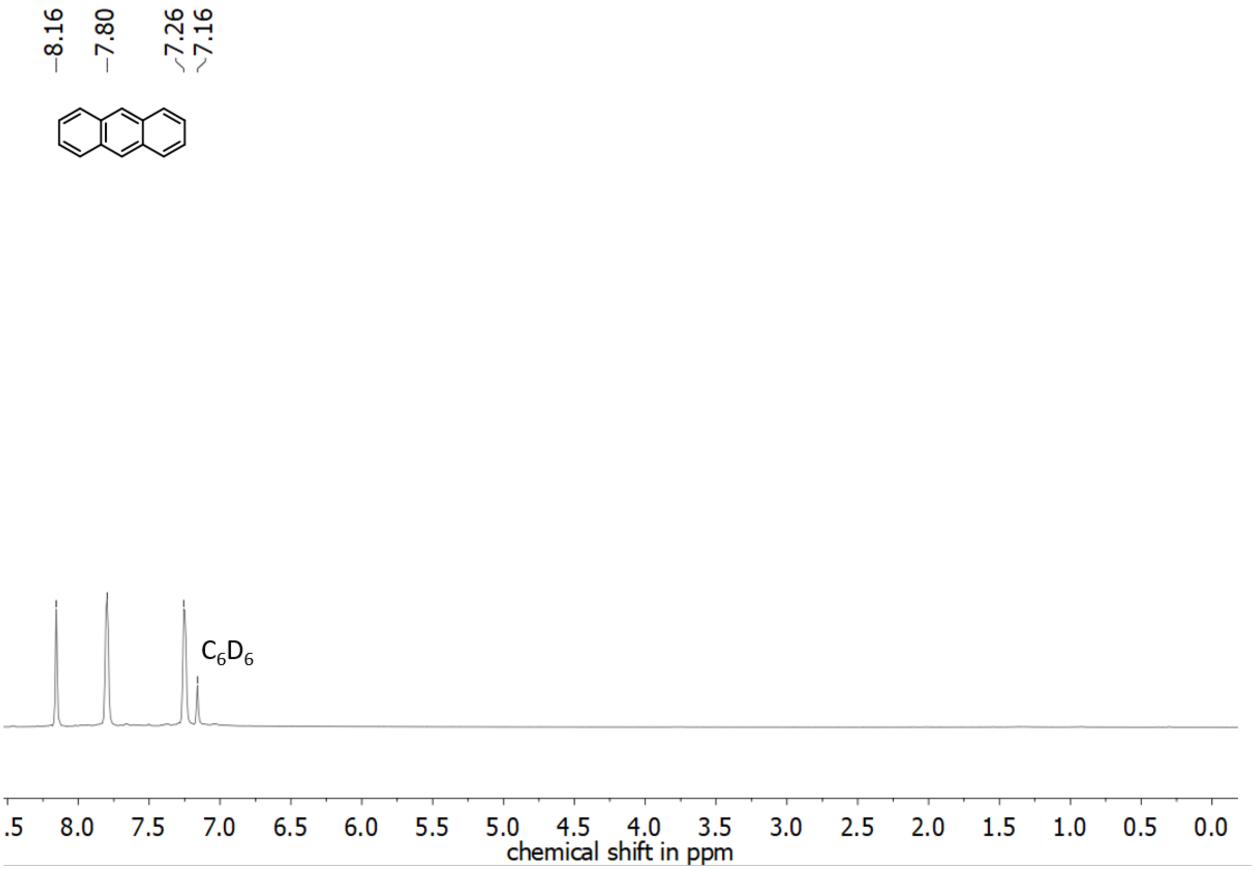


**Figure S38: $^1$H NMR spectrum (600 MHz, $C_6D_6$, 25°C) of anthracene.**

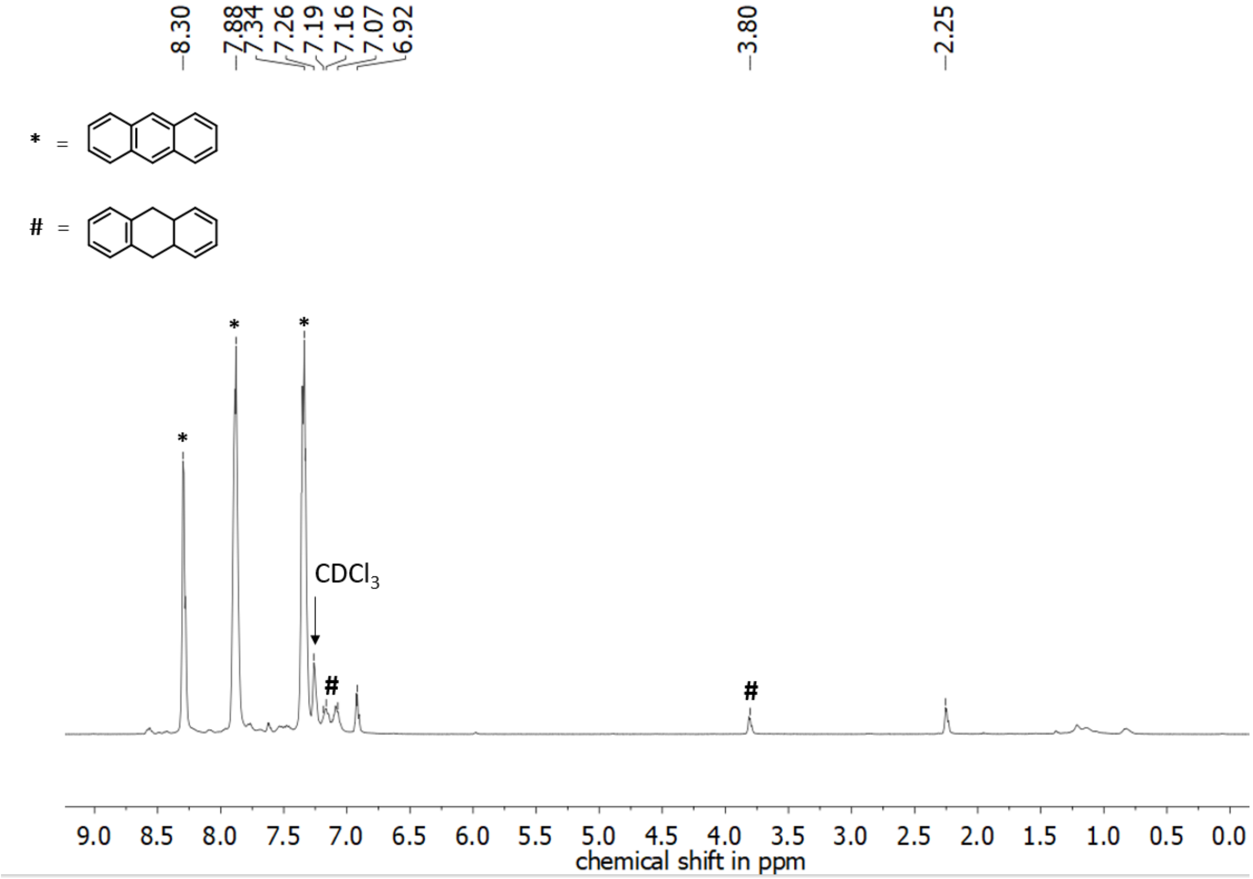


**Figure S39: $^{1}$H NMR spectrum (600 MHz, $C_6D_6$, $CDCl_3$, 25°C) of anthracene after attempted hydrogenation (1M, $C_6D_6$) using 5 mol% Ba(0) and 12 bars of $H_2$ at 120°C (24 h). The sample was dilute with $CDCl_3$ (0.3 mL) to enhance solubility.**

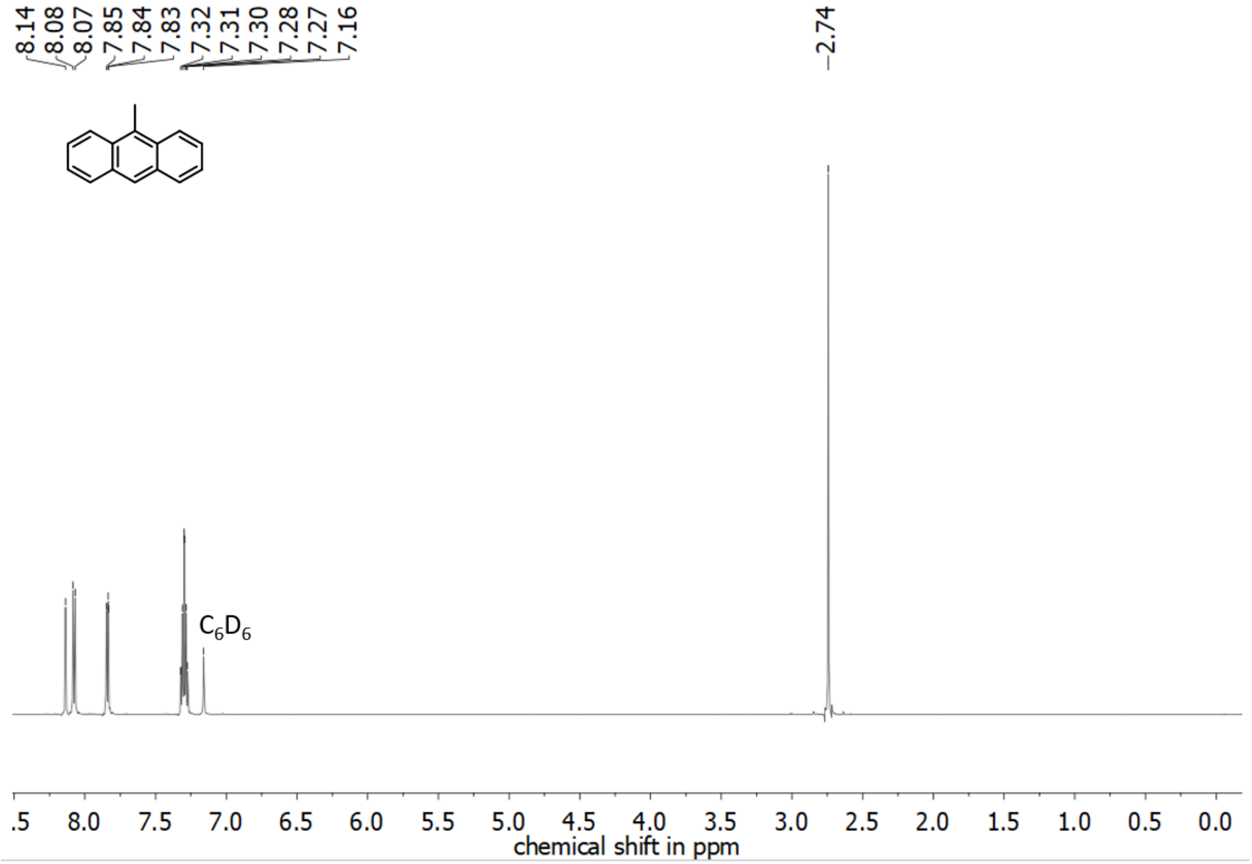


**Figure S40: $^{1}$H NMR spectrum (600 MHz, $C_6D_6$, 25°C) of 9-methylanthracene.**

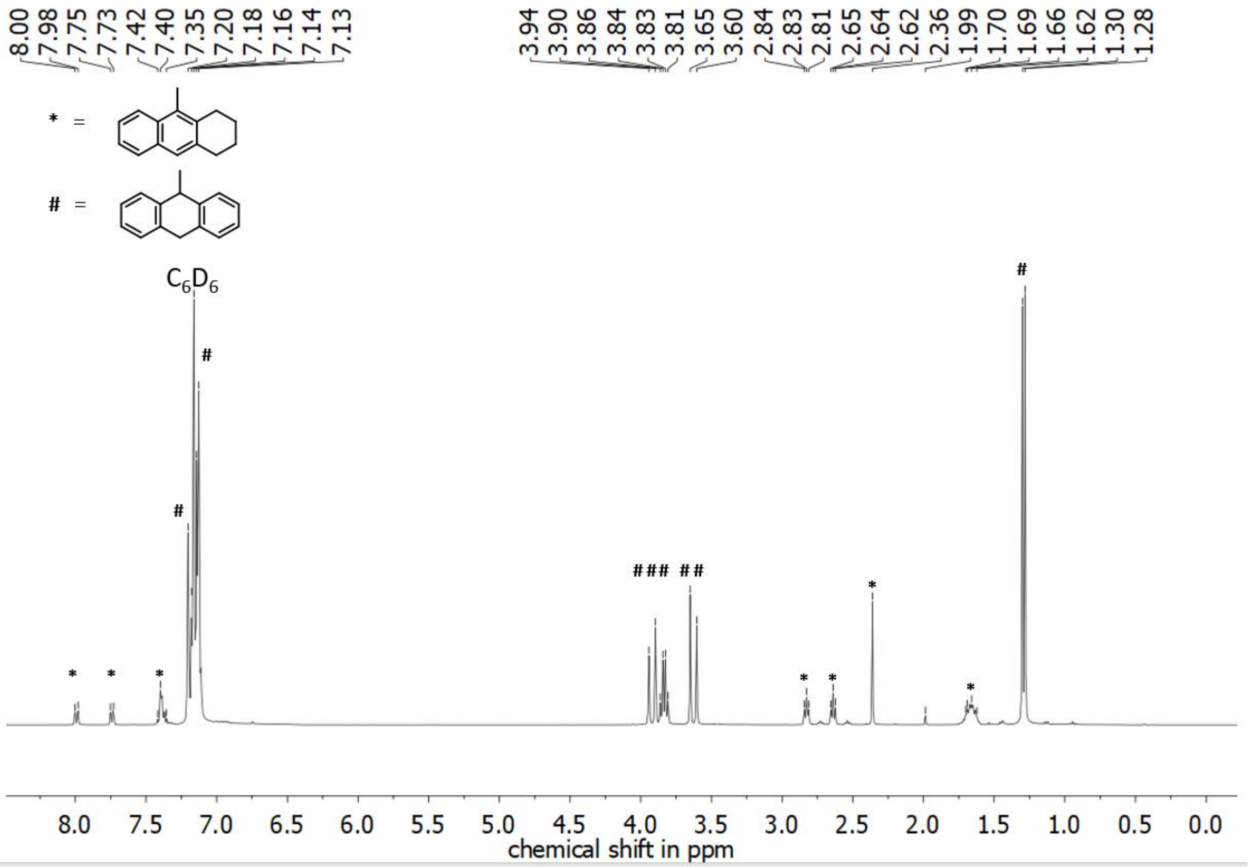


**Figure S41: $^{1}H$ NMR spectrum (600 MHz, $C_6D_6$, 25°C) of 9-methyl-anthracene after catalytic hydrogenation (1M, $C_6D_6$) using 5 mol% Ba(0) and 20 bars of $H_2$ at 120°C (25 h).**

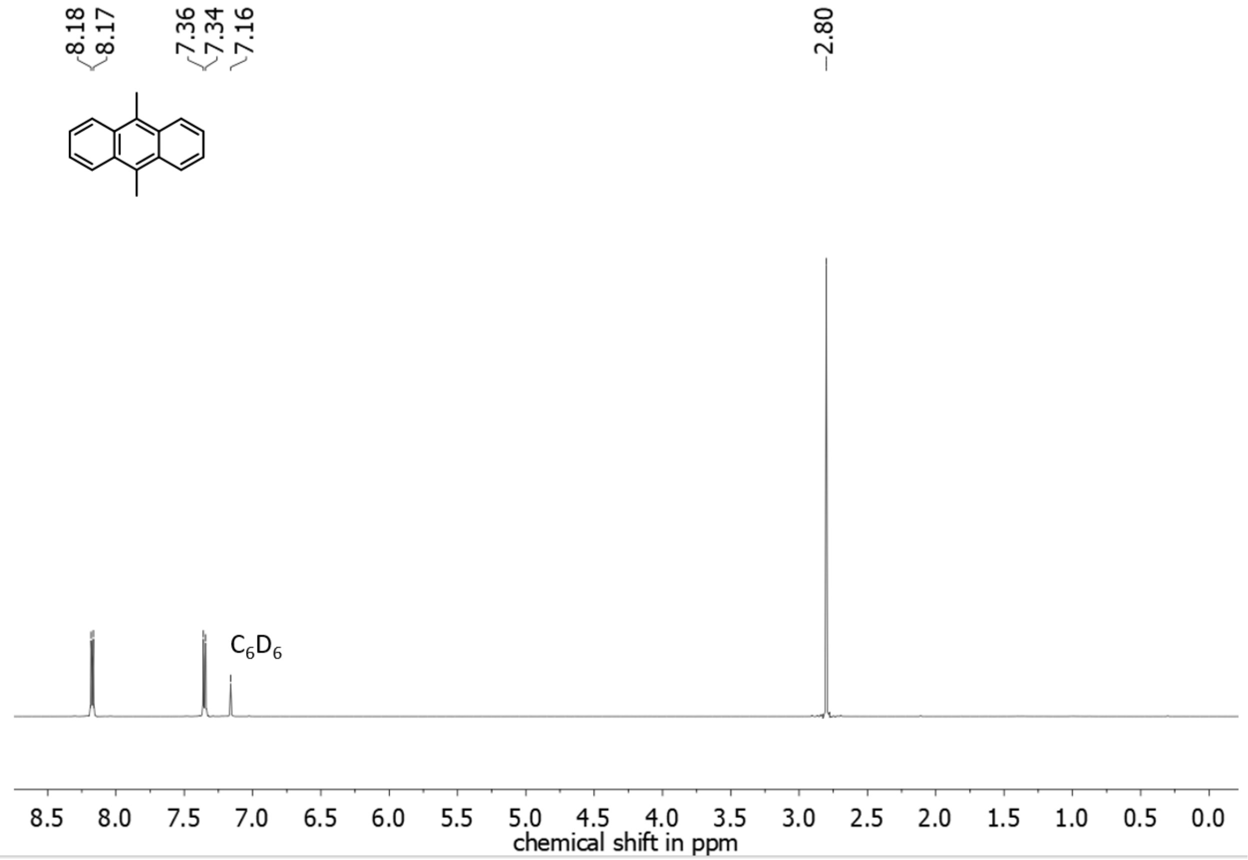


**Figure S42: $^{1}$H NMR spectrum (600 MHz, $C_6D_6$, 25°C) of 9,10-dimethylanthracene.**

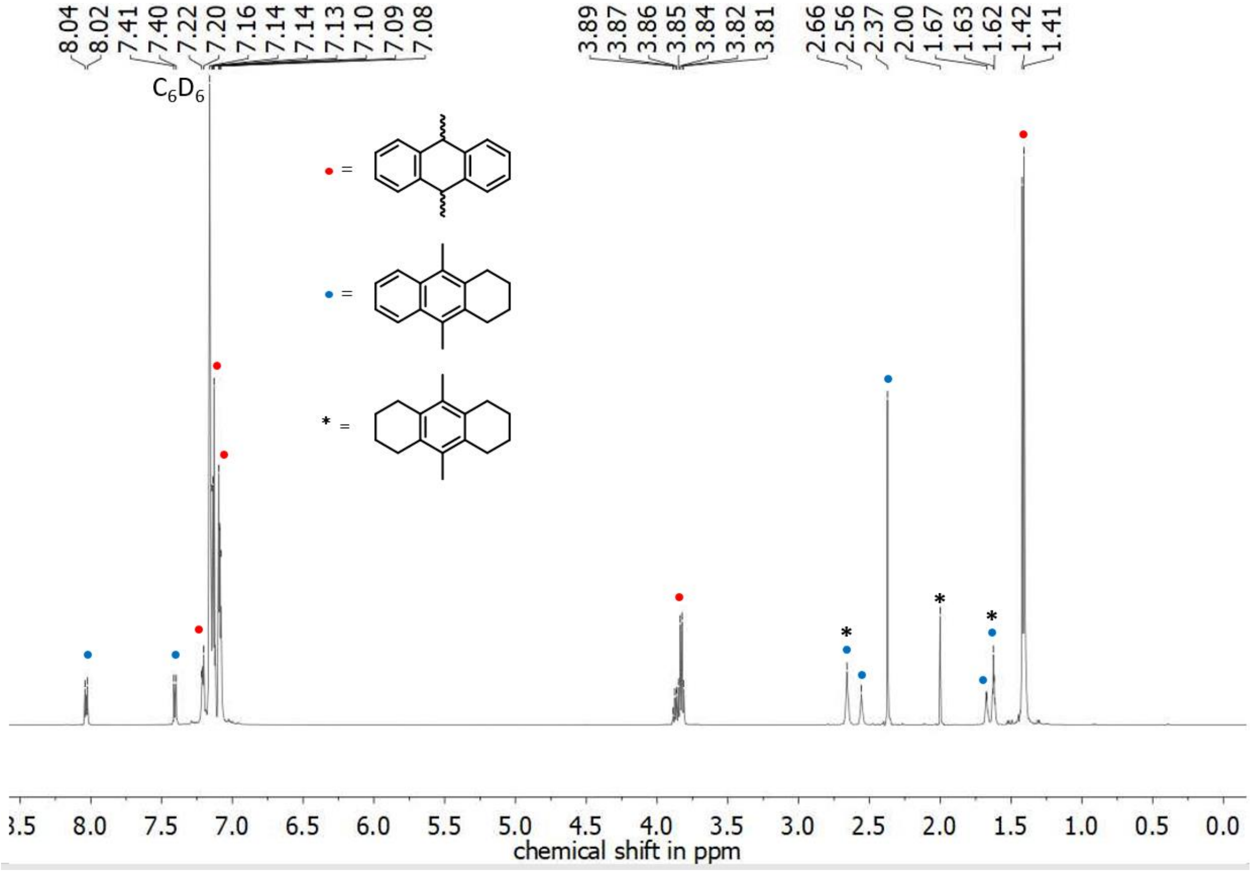


**Figure S43: $^{1}$H NMR spectrum (600 MHz, $C_6D_6$, 25°C) of 9,10-dimethylanthracene after catalytic hydrogenation (1M, $C_6D_6$) using 10 mol% Ba(0) and 50 bars of $H_2$ at 120°C (24 h). 9,10-dimethyl-9,10-dihydroanthracene is obtained as a mixture of *cis*- and *trans*-isomers. Conversion was determined by GC/MS.**

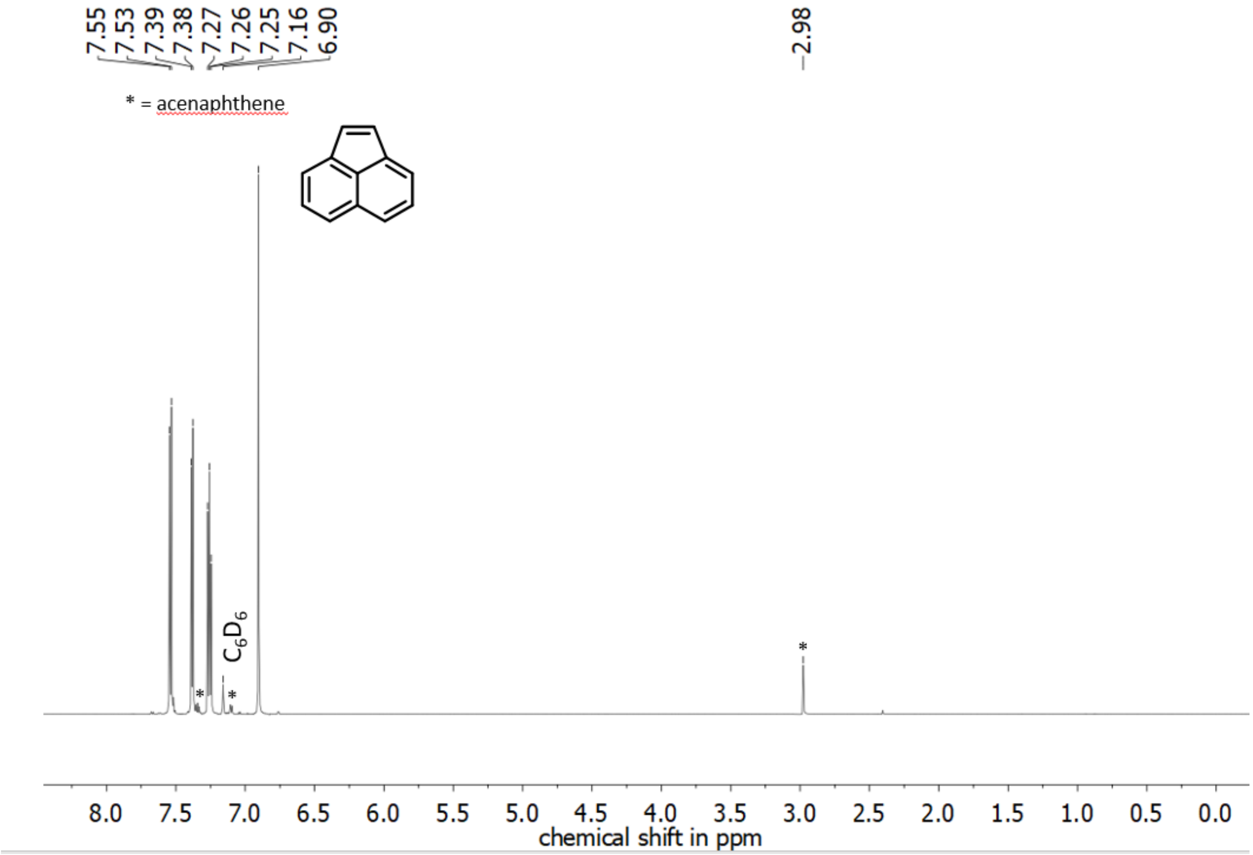


**Figure S44: $^{1}$H NMR spectrum (600 MHz, $C_6D_6$, 25°C) of acenaphthylene. The spectrum shows 4% acenaphthene as trace impurity, evidenced by GC/MS. The acenaphthene contamination could not be removed by sublimation.**

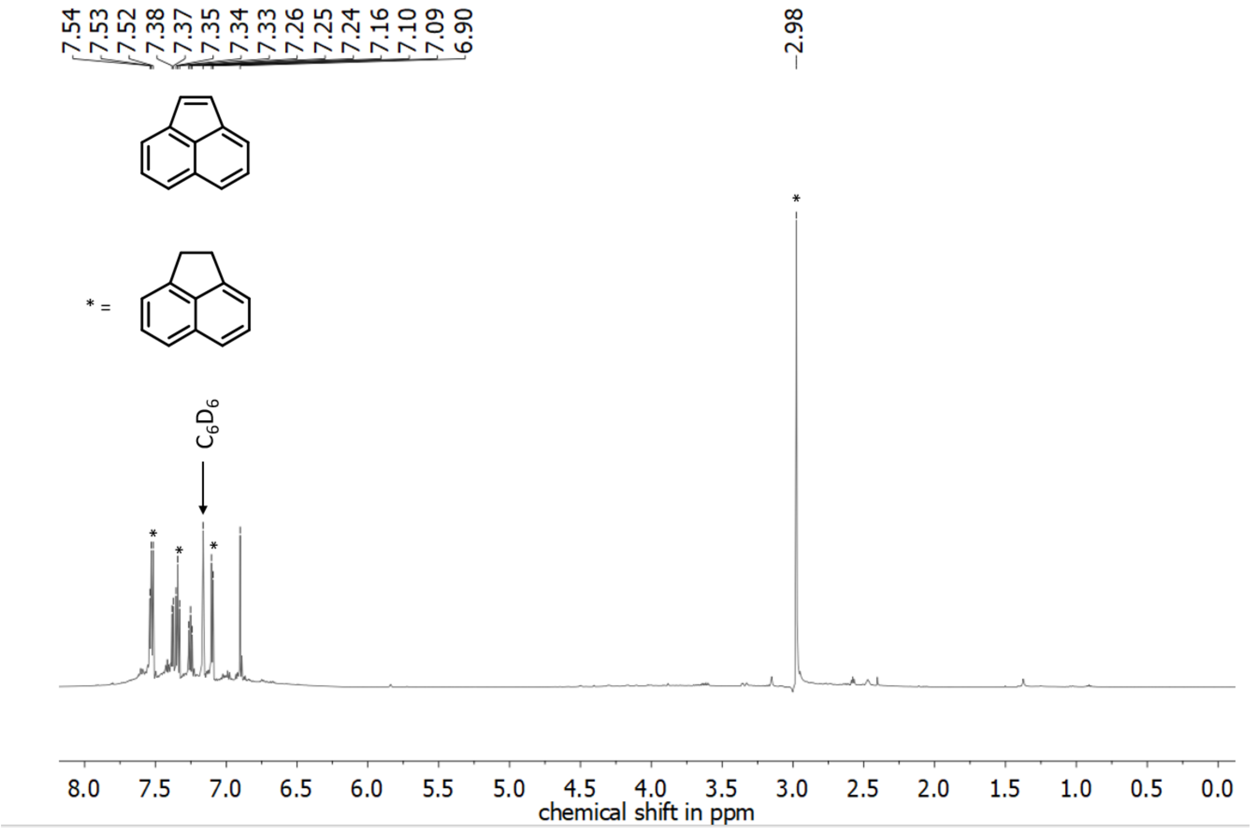


**Figure S45: $^{1}$H NMR spectrum (600 MHz, $C_6D_6$, 25°C) of acenaphthylene after catalytic hydrogenation (1M, $C_6D_6$) using 10 mol% Ba(0) and 50 bars of $H_2$ at 150°C (24 h).**

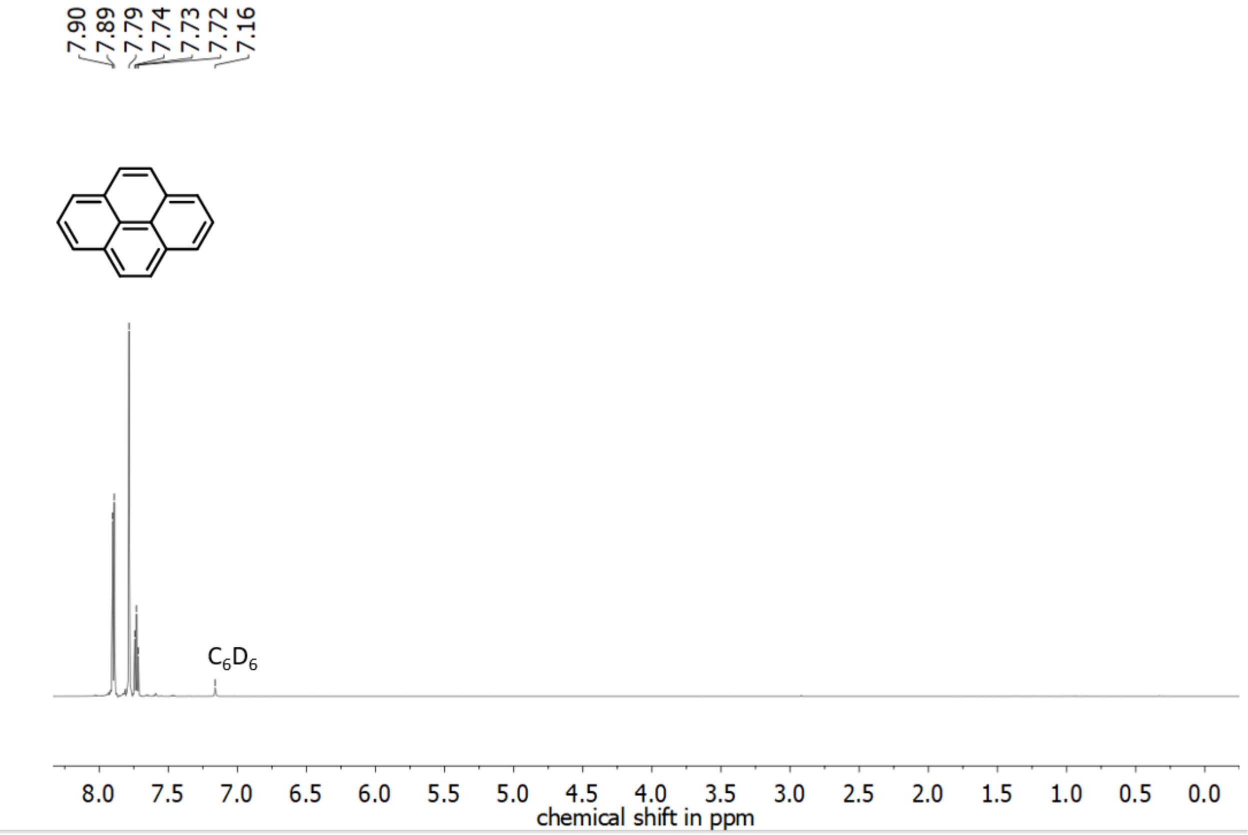


**Figure S46: $^{1}$H NMR spectrum (600 MHz, $C_6D_6$, 25°C) of pyrene.**

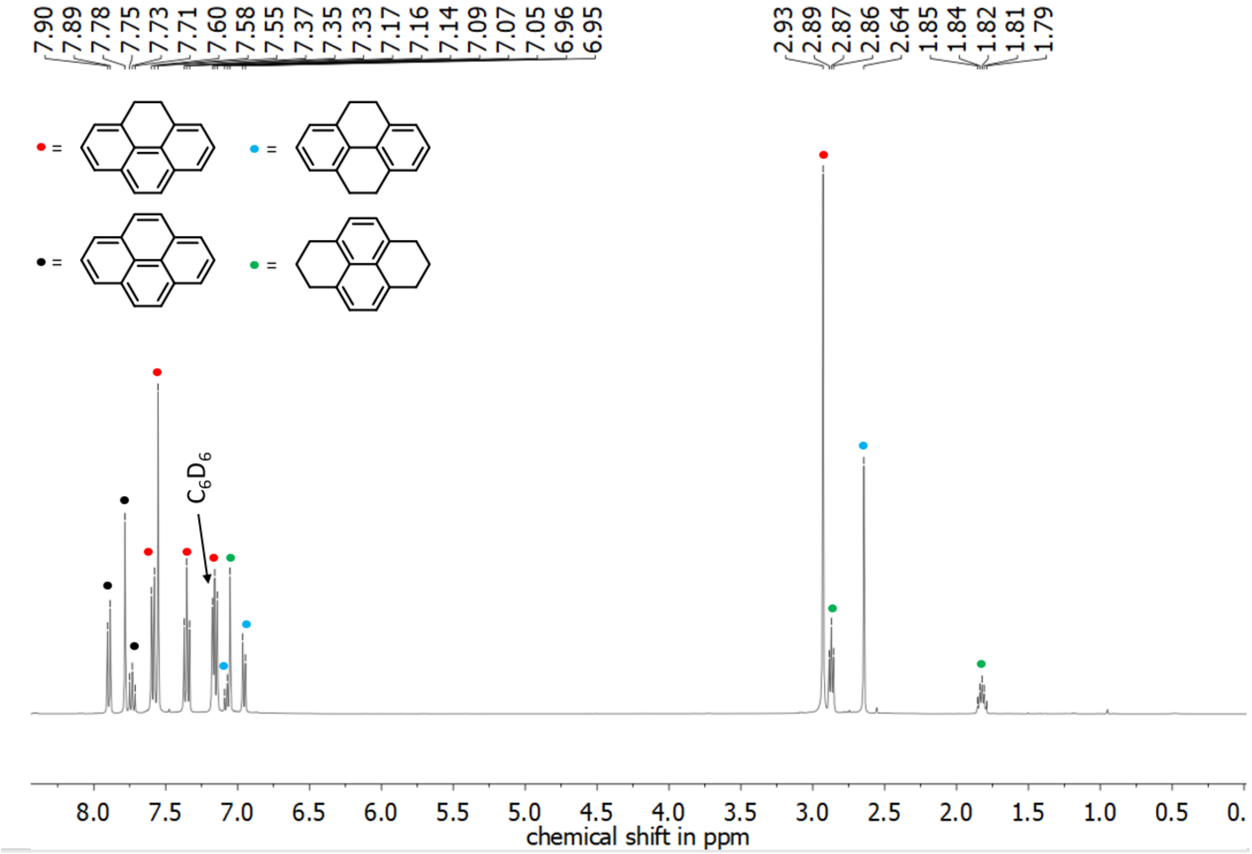


**Figure S47: $^{1}$H NMR spectrum (600 MHz, $C_6D_6$, 25°C) of pyrene after catalytic hydrogenation (1M, $C_6D_6$) using 10 mol% Ba(0) and 50 bars of $H_2$ at 150°C (24h).**

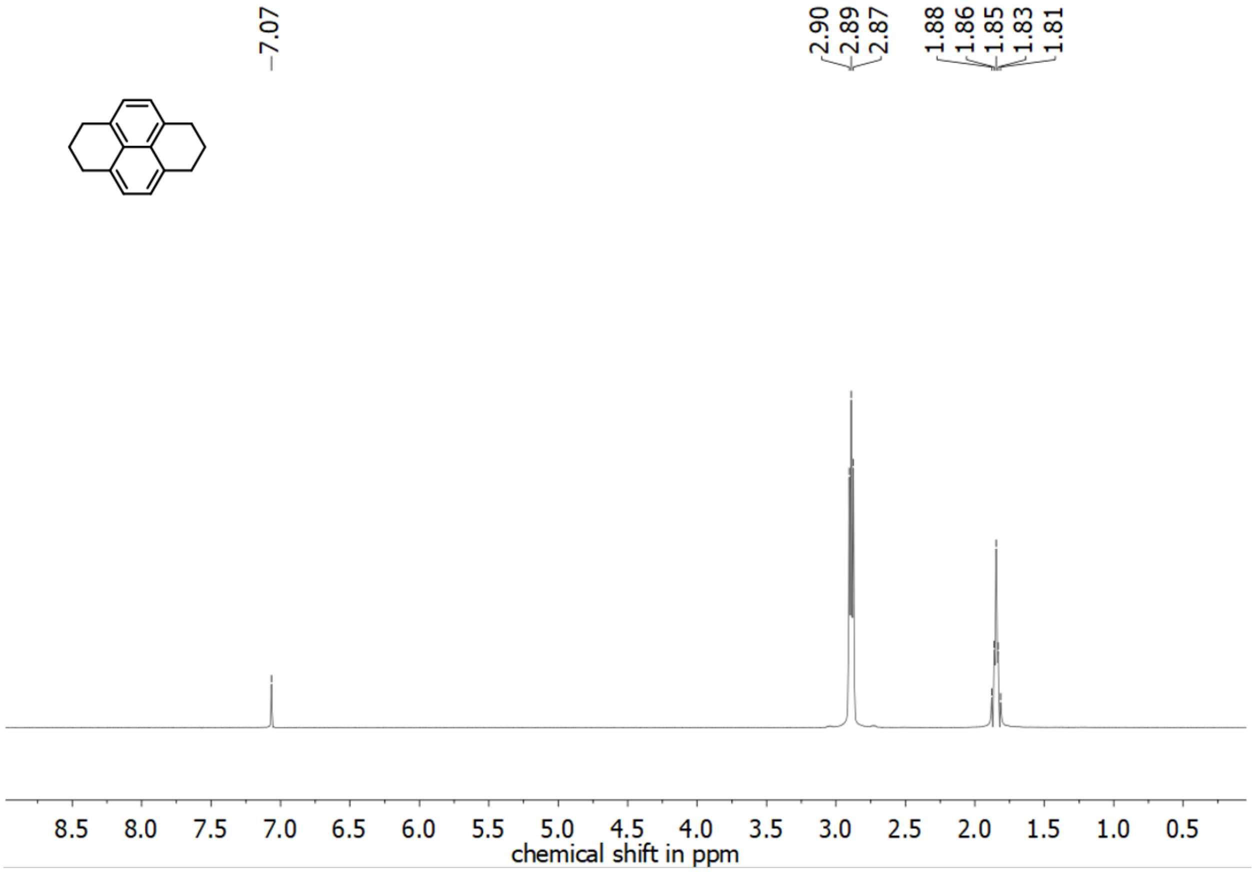


**Figure S48: Selective TOCSY NMR spectrum (600 MHz, $C_6D_6$, 25°C) of pyrene after catalytic hydrogenation (1M, $C_6D_6$) using 10 mol% Ba(0) and 50 bars of $H_2$ at 150°C (24h).**

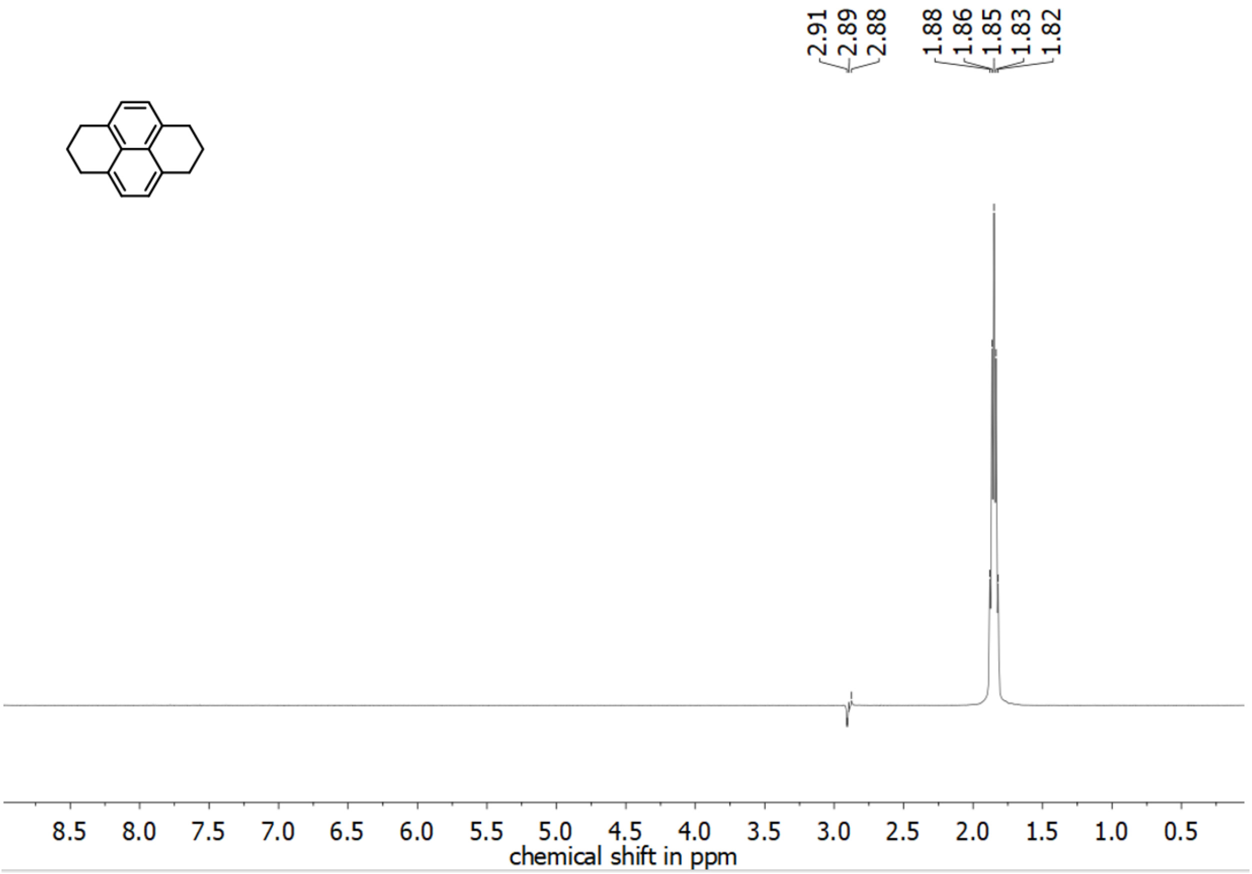


**Figure S49: Selective NOESY NMR spectrum (600 MHz, $C_6D_6$, 25°C) of pyrene after catalytic hydrogenation (1M, $C_6D_6$) using 10 mol% Ba(0) and 50 bars of $H_2$ at 150°C (24h).**

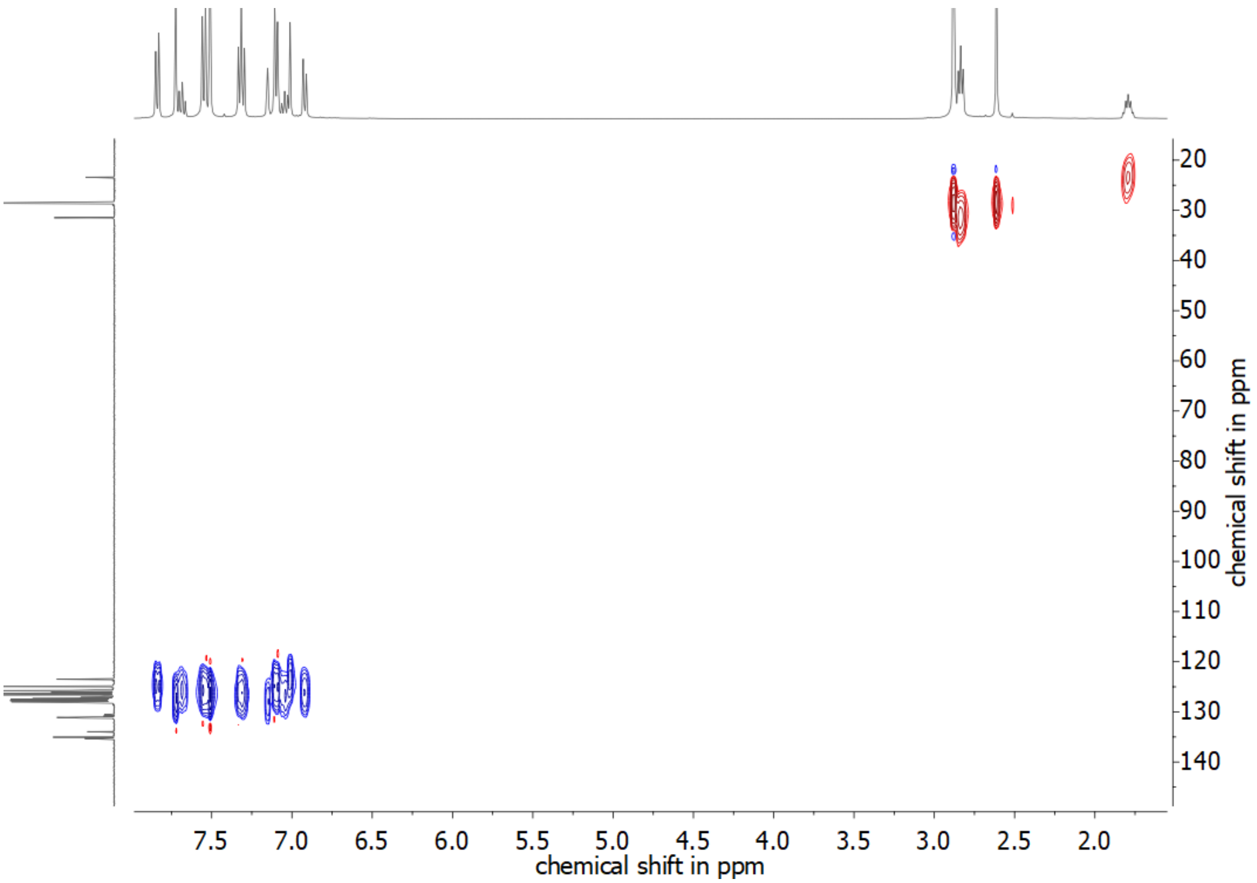


**Figure S50: $^{1}H$ $^{13}C$ HSQC NMR spectrum (600 MHz, $C_6D_6$, 25°C) of pyrene after catalytic hydrogenation (1M, $C_6D_6$) using 10 mol% Ba(0) and 50 bars of $H_2$ at 150°C (24h).**

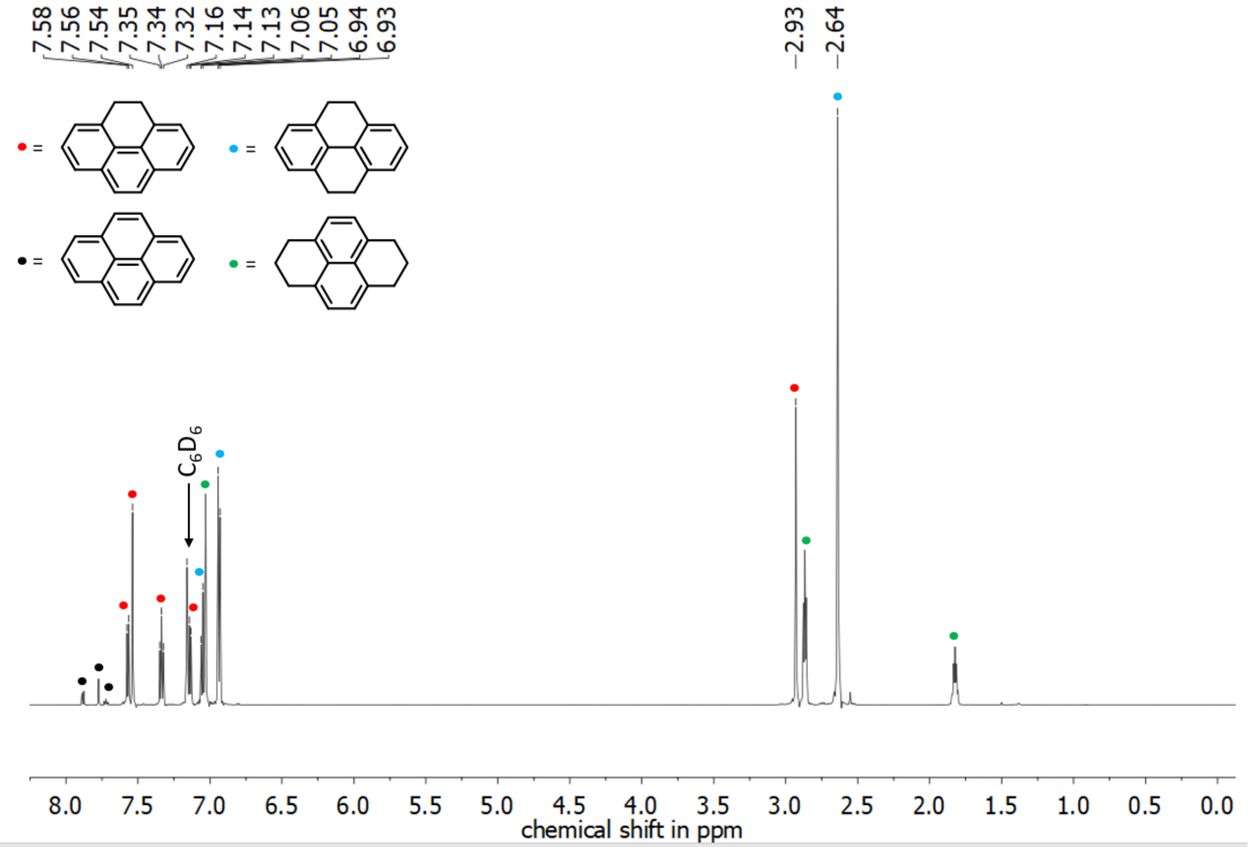


**Figure S51:** [1]**H NMR spectrum (600 MHz, $C_6D_6$, 25°C) of pyrene after catalytic hydrogenation (1M, $C_6D_6$) using 10 mol% Ba(0) and 50 bars of $H_2$ at 150°C (48h).**

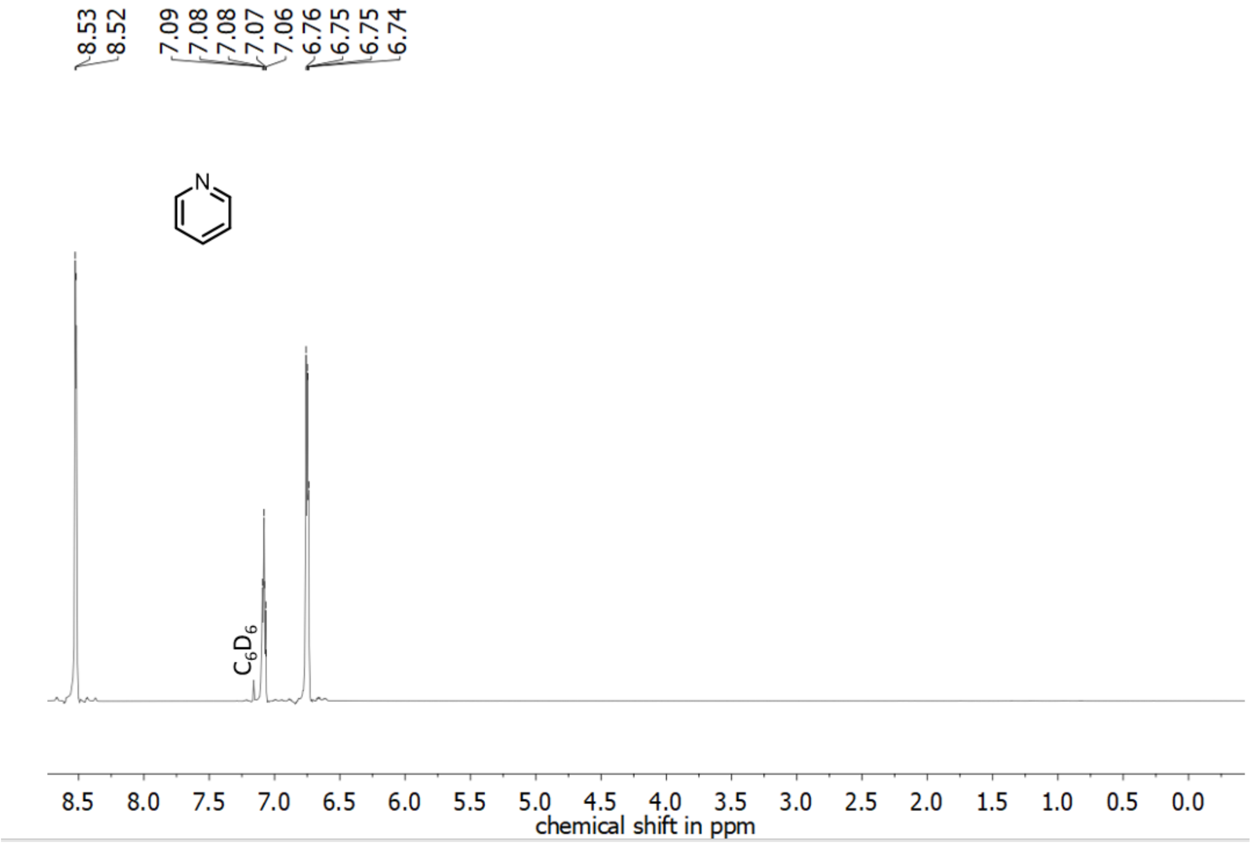


**Figure S52: $^{1}$H NMR spectrum (600 MHz, $C_6D_6$, 25°C) of pyridine.**

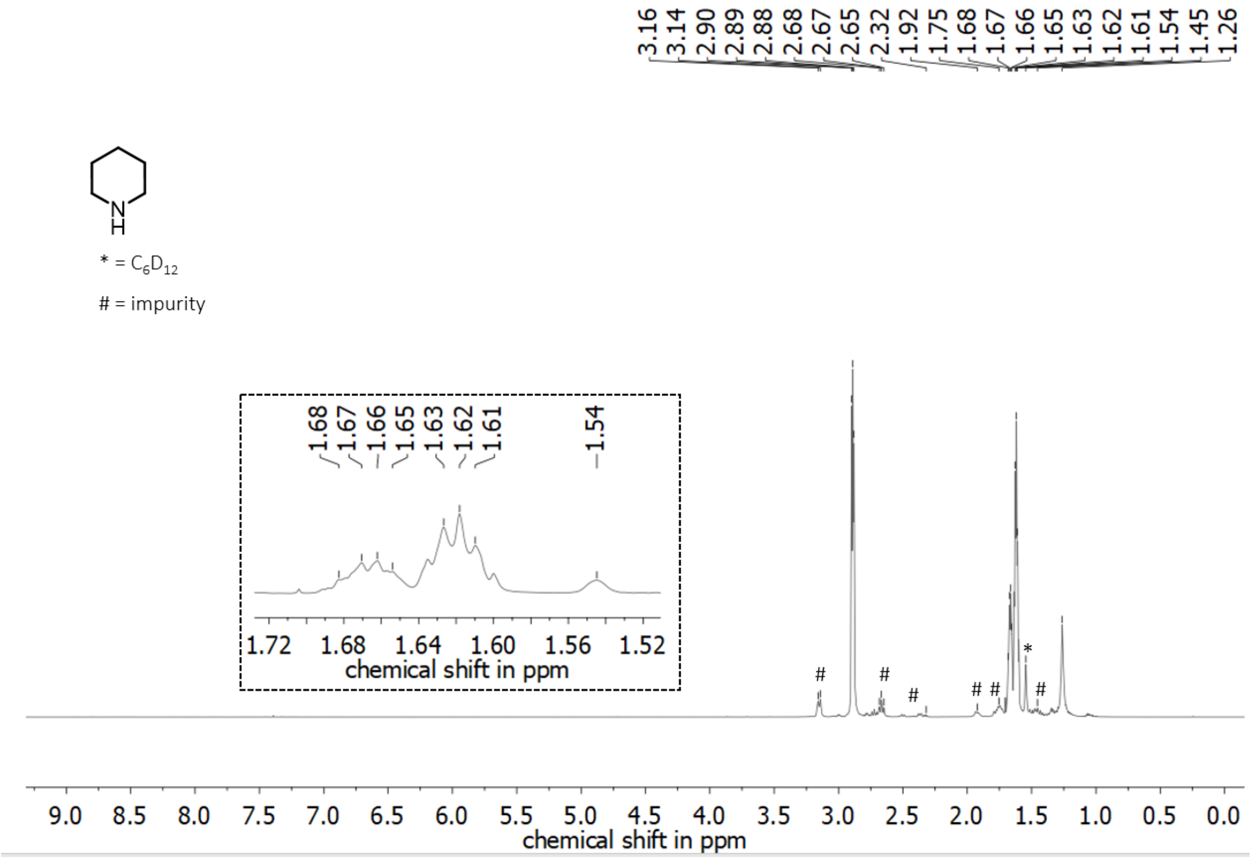


**Figure S53: $^{1}$H NMR spectrum (600 MHz, $C_6D_6$, 25°C) of pyridine after catalytic hydrogenation (1M, $C_6D_{12}$) using 10 mol% Ba(0) and 50 bars of $H_2$ at 150°C (89 h). # is an unknown impurity which is likely polymeric in nature (no GC/MS detection).**

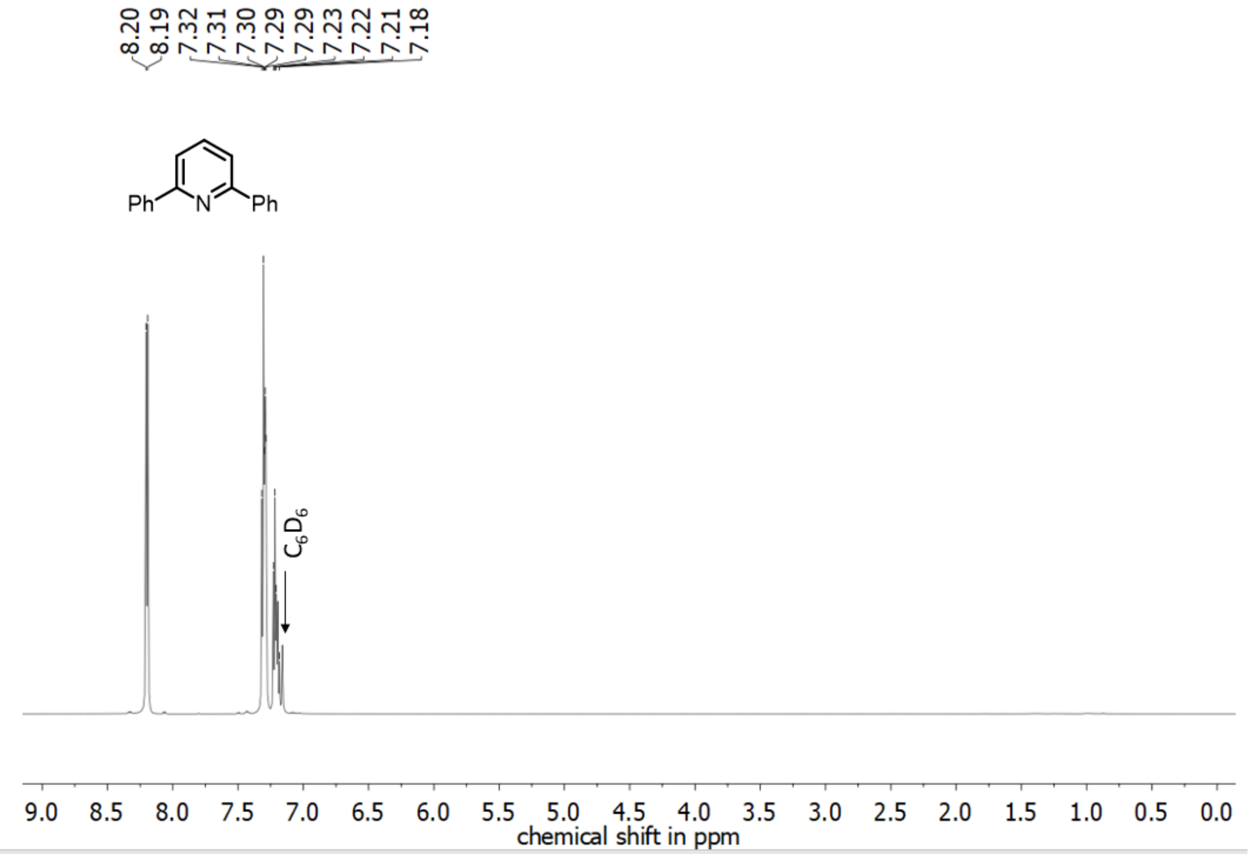


**Figure S54: $^{1}$H NMR spectrum (600 MHz, $C_6D_6$, 25°C) of 2,6-diphenyl-pyridine.**

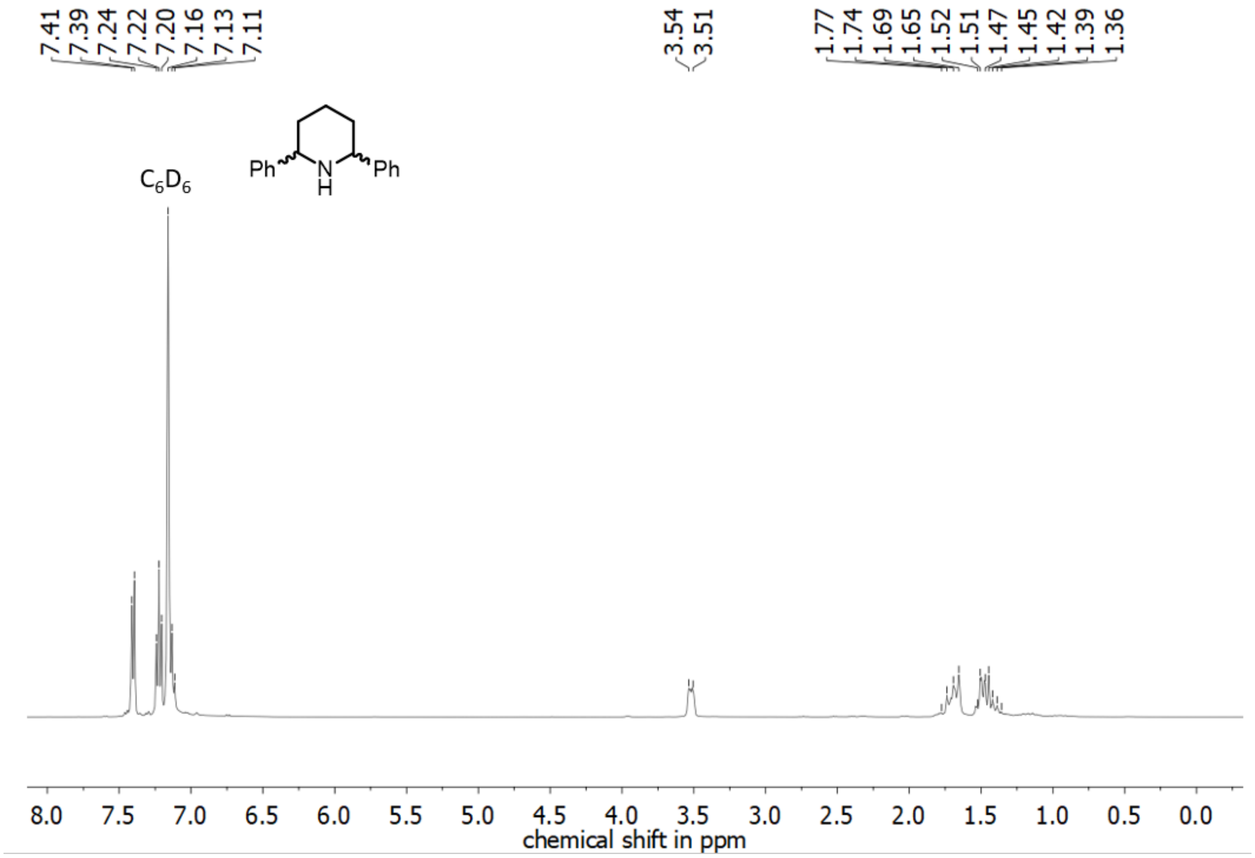


**Figure S55: $^{1}$H NMR spectrum (600 MHz, $C_6D_6$, 25°C) of 2,6-diphenyl-pyridine after catalytic hydrogenation (1M, $C_6D_6$) using 10 mol% Ba(0) and 50 bars of $H_2$ at 150°C (24 h). 2,6-diphenylpipridine is obtained as a mixture of *cis*- and *trans*-isomers.**

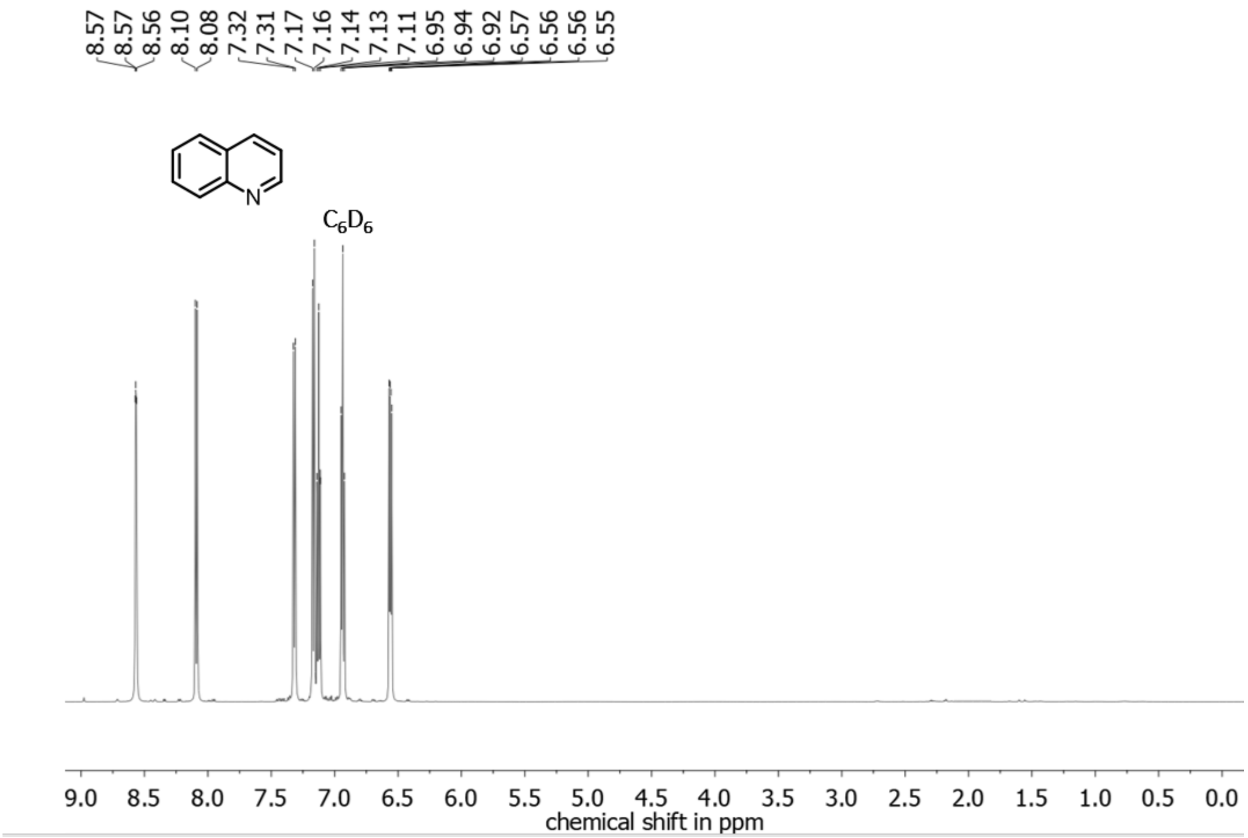


**Figure S56: $^1$H NMR spectrum (600 MHz, $C_6D_6$, 25°C) of quinoline.**

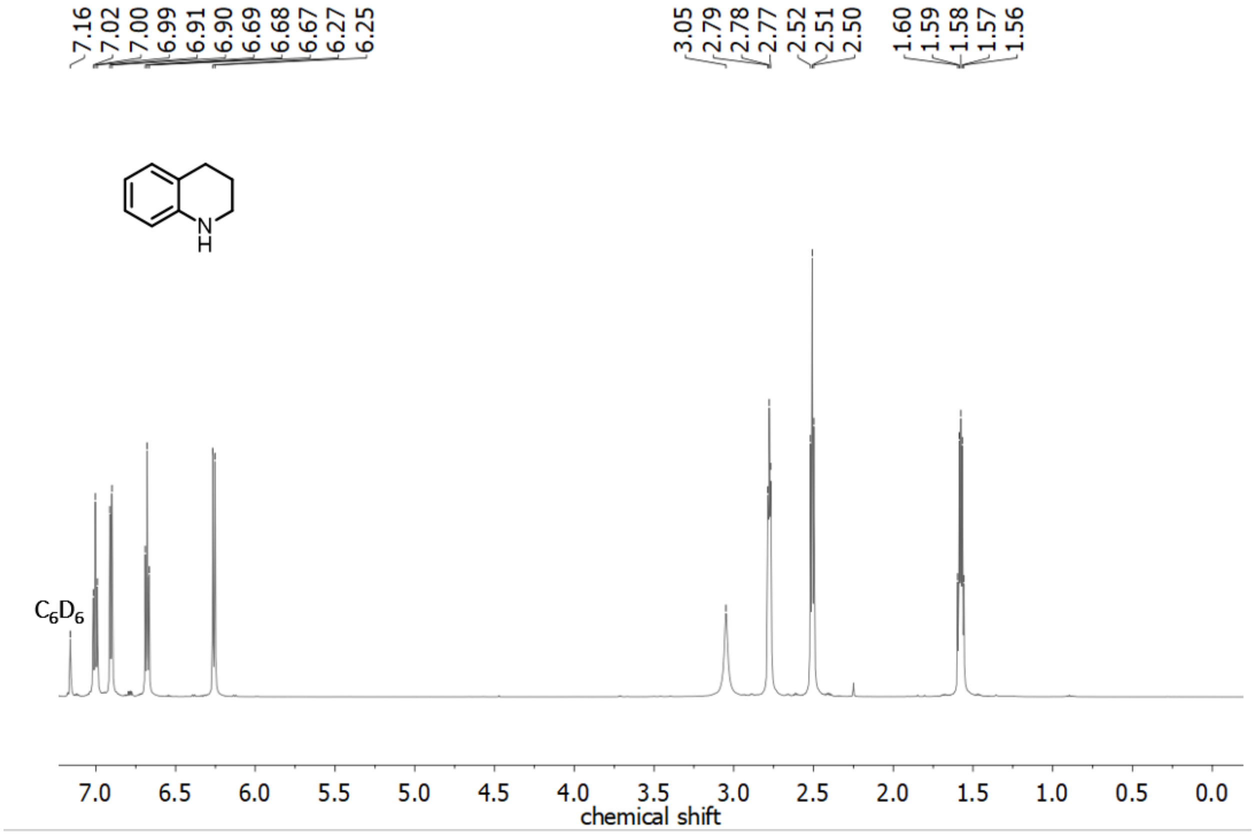


**Figure S57: $^1$H NMR spectrum (600 MHz, $C_6D_6$, 25°C) of quinoline after catalytic hydrogenation (1M, $C_6D_6$) using 10 mol% Ba(0) and 20 bars of $H_2$ at 135°C (24 h).**

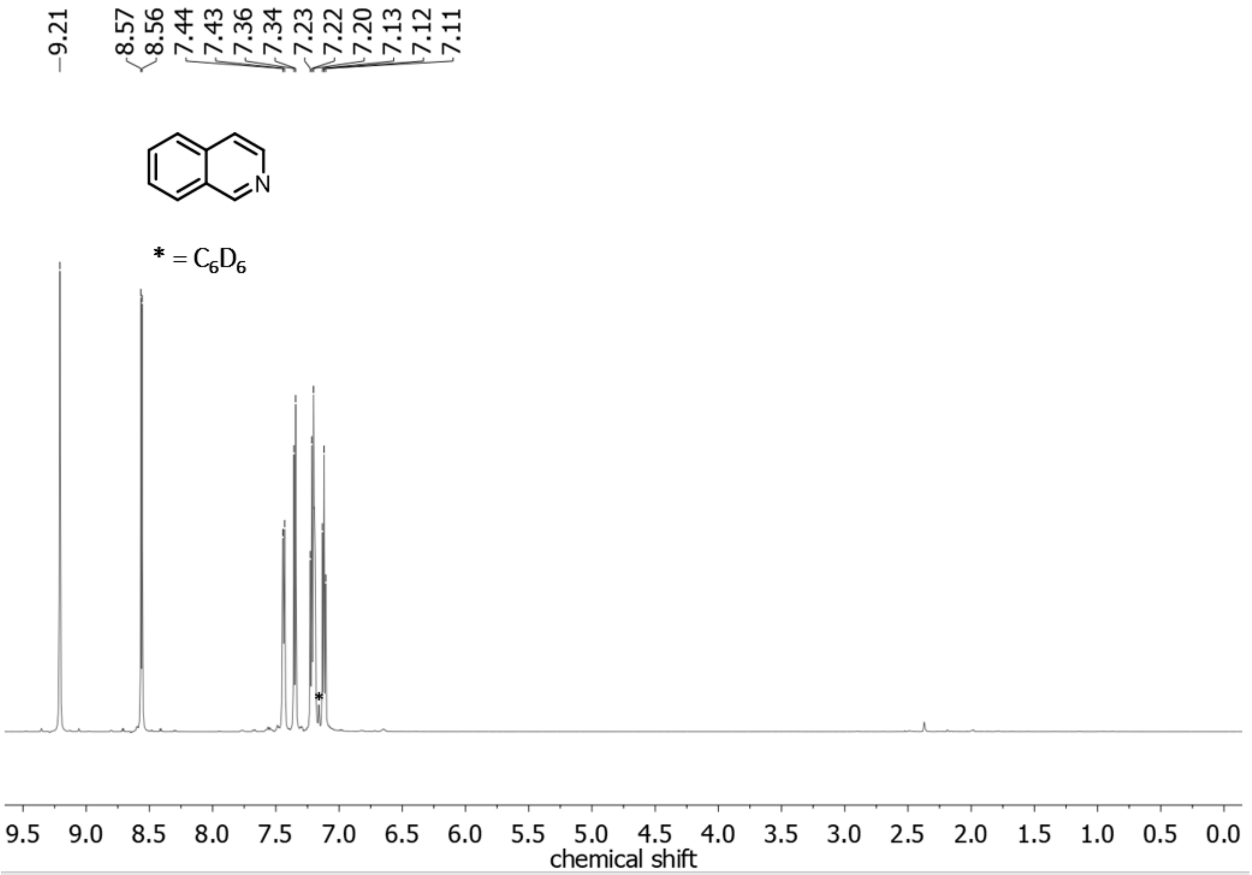


**Figure S58: $^{1}$H NMR spectrum (600 MHz, $C_6D_6$, 25°C) of *iso*-quinoline.**

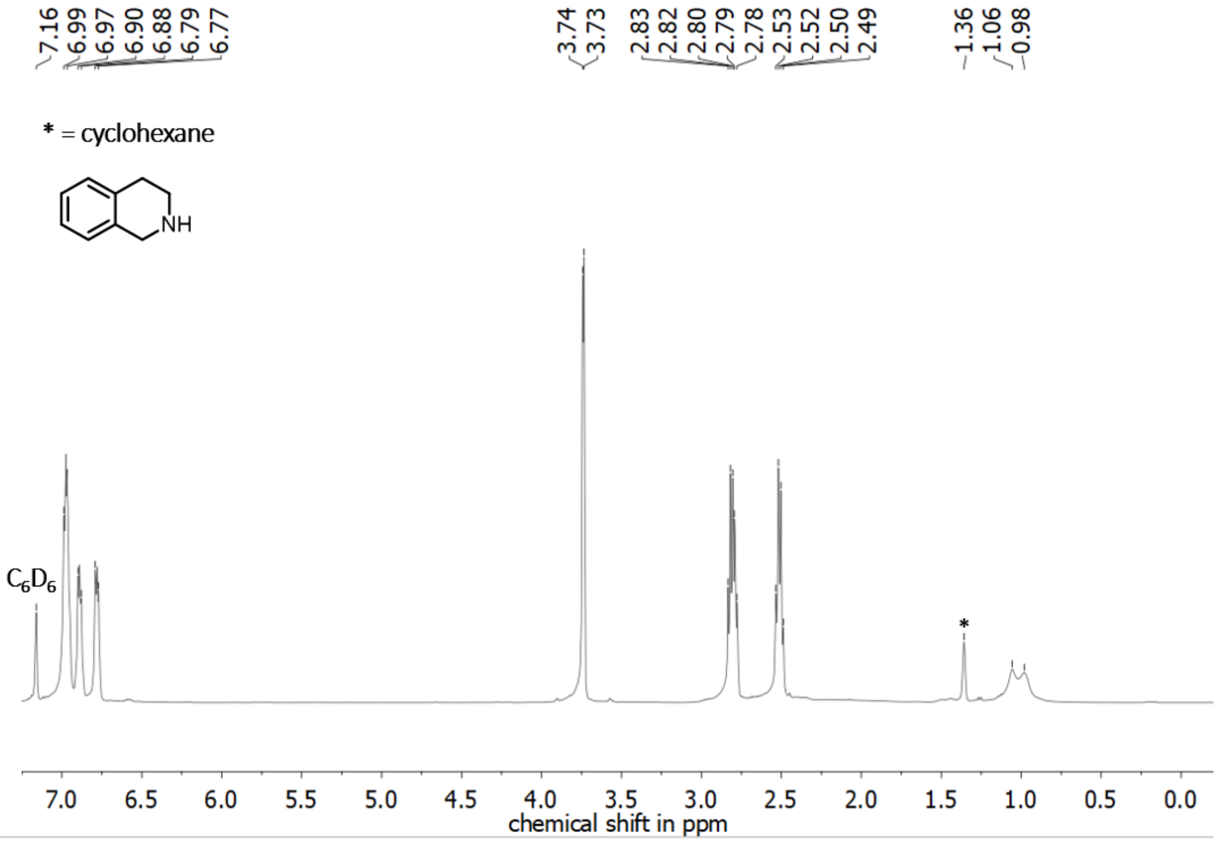


**Figure S59: $^1$H NMR spectrum (600 MHz, $C_6D_6$, 25°C) of *iso*-quinoline after catalytic hydrogenation (1M, $C_6D_6$) using 10 mol% Ba(0) and 50 bars of $H_2$ at 150°C (65 h). The spectrum shows traces of cyclohexane (partially deuterated), due to competing solvent hydrogenation.**

## 1.5 Deuteration Experiments

An oven-dried miniature stainless steel autoclave (15 mL) containing a magnetic stir bar was charged with the Ba(0) pre-catalyst (0.11 mmol, 1.0 mol%, 15 mg), $C_6D_6$ (1 mL) and adamantane (20 mg, 0.15 mmol) in an $N_2$-filled Glovebox. The tightly sealed pressure vessel was transferred out of the glovebox and connected to a $H_2$ gas manifold. The supply line was evacuated for 1 minute and subsequently purged with dry gas. This process was repeated for three times using $N_2$ and for three further times using $H_2$ to ensure the exclusion of air and moisture. The reactor was then pressurized with $H_2$ (50 bar) and heated to 120°C in a pre-heated aluminum metal block and stirred for 2h. Subsequently the autoclave was cooled to room temperature in a water bath and subsequently vented. The reaction mixture was filtered through a glass microfiber filter in a Pasteur pipette and the crude filtrate was analyzed by $^{1}H$ NMR spectroscopy without additional purification. Conversion was determined via $^{1}H$ NMR considering the increased $C_6D_5H$-peak of the internal reference (adamantane). A TON of 221 could be extracted which corresponds to a TOF of 111/h. After the reaction time notable amounts of cyclohexane could also be detected, due to competing solvent hydrogenation.

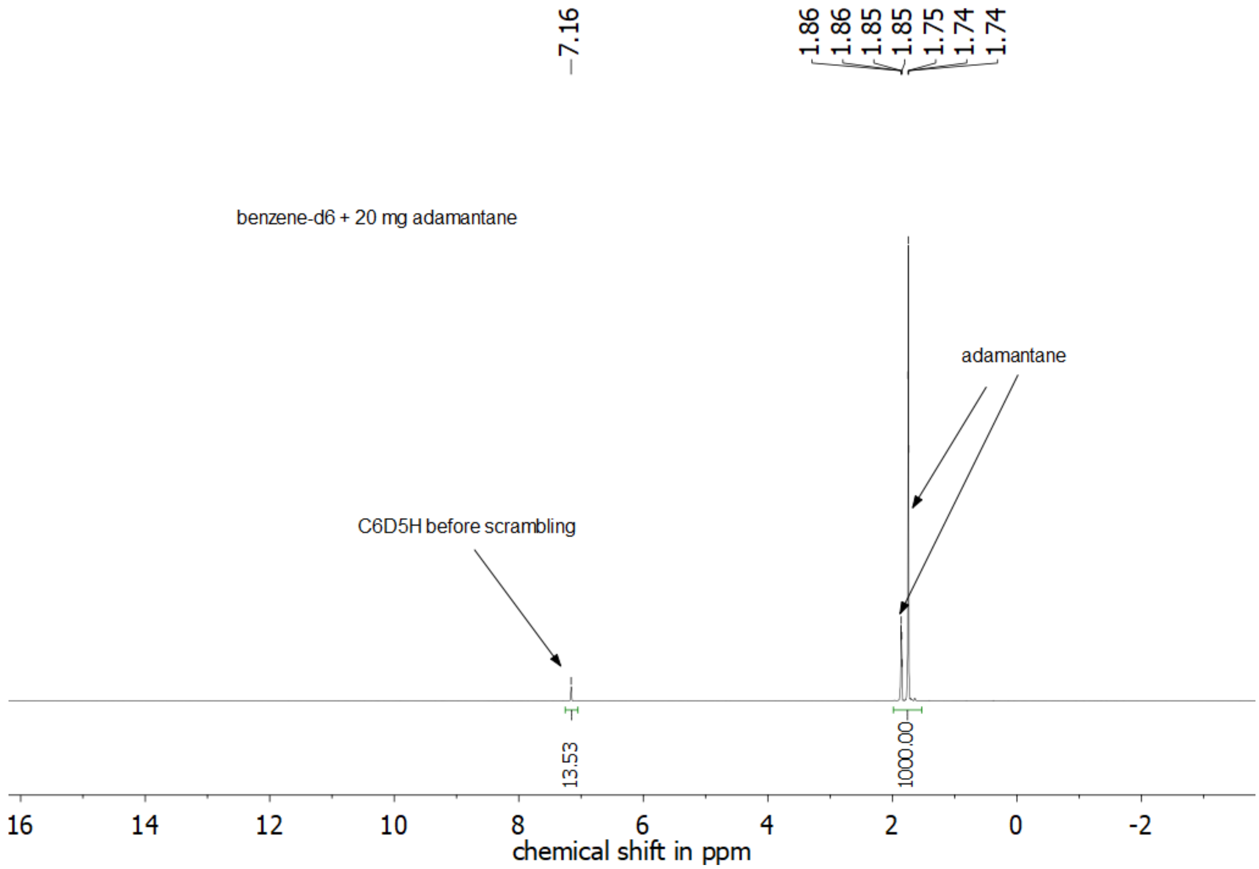


**Figure S60: $^{1}$H NMR spectrum (600 MHz, $C_6D_6$, 25°C) before H/D exchange of $C_6D_6$ and $H_2$ using 1 mol% Ba(0) and adamantane as an internal standard.**

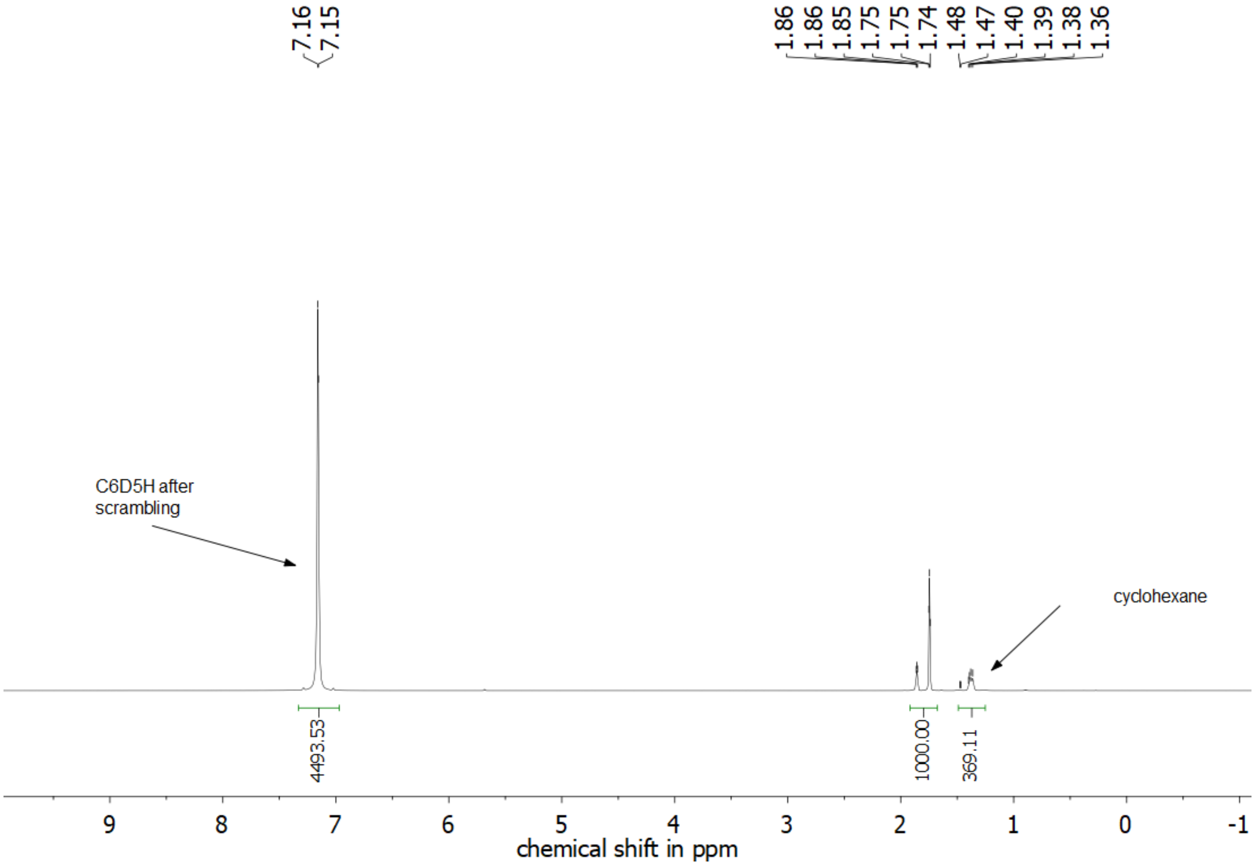


**Figure S61: $^{1}$H NMR spectrum (600 MHz, $C_6D_6$, 25°C) of H/D exchange of $C_6D_6$ (neat) and $H_2$ (50 bars) using 1 mol% Ba(0) at 120°C. The spectrum shows cyclohexane, due to competing solvent hydrogenation and the internal standard adamantane.**

## 1.6 Stoichiometric Experiments

### Reaction of Ba(0) with $H_2$:

An oven-dried miniature stainless steel autoclave (15 mL) containing a magnetic stir bar was charged with activated, black, Ba(0) powder (0.73 mmol, 100 mg) and $C_6H_{12}$ (1 mL) in a $N_2$-filled glovebox. The tightly sealed pressure vessel was transferred out of the glovebox and connected to a $H_2$ gas manifold. The supply line was evacuated for 1 minute and subsequently purged with dry $N_2$. To ensure the exclusion of air and moisture, this process was repeated for three times using $N_2$ and for three further times using $H_2$. The reactor was then pressurized with $H_2$ (50 bar) and either (a) stirred at room temperature for 24 h or (b) heated to 120°C in a pre-heated aluminum metal block and stirred for 24 h. Subsequently the autoclave was cooled to room temperature, vented and opened inside a glovebox. The grey powdery product was rinsed of the reactor walls with $C_6H_{12}$ (1 mL), transferred to a Schlenk flask and separated via centrifugation in a Schlenk centrifuge. Subsequently the grey powder was dried under vacuum. Yield: 100 mg, yield: 99%. Given the fact that it is very hard to completely convert Ba metal to $BaH_2$ at low temperature,[S6] the grey powdery product is most likely a mixture of Ba(0) and $BaH_2$.

### Reaction of Ba(0)/$BaH_2$ with MeOD:

The grey powder formed by reaction of Ba(0) and hydrogen (10 mg, 0.07 mmol), either at room temperature or at 120 °C, was suspended in $C_6D_6$ (500 µL) and THF-$d_8$ (50 µL) in a J. Young tube. Subsequently a solution of MeOD (3 µL, 74.2 µmol) in $C_6D_6$ (27 µL) was added and the NMR tube was closed while the reaction was monitored via $^1$H NMR. $^1$H NMR spectra of the quenched products are in both cases ($BaH_2$ prepared at room temperature or $BaH_2$ prepared at 120 °C) similar (Figures S62-S63). The spectra show formation of H-D and $H_2$ (formed by H/D exchange). This is evidence for the formation of $BaH_2$ from Ba(0) and $H_2$ (at room temperature and at 120 °C).

**Reaction of Ba(0)/$BaH_2$ with $Me_3SiCl$:**

The grey powder formed by reaction of Ba(0) and hydrogen at 120 °C (10 mg, 0.07 mmol) was suspended in THF-$d_8$ (550 µL) in a J. Young tube. Subsequently a solution of $Me_3SiCl$ (9 µL, 70.9 µmol) in $C_6D_6$ (91 µL) was added and the NMR tube was closed while the reaction was monitored via $^1$H and $^{29}$Si NMR. $Me_3SiH$, $(Me_3Si)_2O$ and unreacted $Me_3SiCl$ were detected (Figure S64-S65). $(Me_3Si)_2O$ is formed over several days via hydrolysis. Formation of $Me_3SiH$ indicates the presence of $BaH_2$.

**Reaction of Ba(0)/$BaH_2$ with pivaldehyde:**

The grey powder formed by reaction of Ba(0) and hydrogen at 120 °C (50 mg, 0.35 mmol) was suspended in $C_6D_6$ (550 µL) in a J. Young tube. Subsequently a solution of pivaldehyde (9 µL, 0.13 mmol) in $C_6D_6$ (91 µL) was added and the NMR tube was closed while the reaction was monitored via $^1$H NMR. The spectrum shows the exclusive formation of 2,2,5,5-tetramethyl-3-hexanone, which was formed via elimination of BaO from 2,2,5,5-tetramethylhexane-3,4-diole. This diol was formed by reductive coupling of pivaldehyde and excess Ba(0) (Figure S66-S70).

**Reaction of Ba(0) with tetraphenylethylene and subsequent MeOD quench:**

Activated, black, Ba(0) powder (10 mg, 0.07 mmol) and tetraphenylethylene (16.6 mg, 0.05 mmol) were suspended in THF-$d_8$ (550 µL) in a J. Young tube. The NMR tube was heated to 75°C for 24h, while the solution turned dark red. Subsequently the tube was cooled to room temperature, opened inside a glovebox and quenched with a solution of MeOD (6 µL, 0.15 mmol) in $C_6D_6$ (54 µL) giving rise to immediate discoloration. The residue was filtered off and the colorless filtrate was dried under reduced pressure, redissolved in $C_6D_6$ and characterized by $^2$D NMR spectroscopy (Figure S71). The spectrum confirms the formation of $Ph_2C(D)$-$(D)CPh_2$. This indicates that Ba(0) and tetraphenylethylene reacted to the dark-red colored electron-transfer product: $[Ph_2C$-$CPh_2]^{2-}$ $Ba^{2+}$.

**Reaction of Ba(0) with *N*,1,1-triphenylmethanimine (TPMI) forming $[Ba(TPMI)(THF)_3]_2$:**

Activated, black, Ba(0) powder (30.0 mg, 220 µmol) was suspended in THF (1.4 mL) in a J. Young tube. *N*,1,1-triphenylmethanimine (30.0 mg, 116.6 µmol) was added and the black suspension immediately changed to a deep, blood-red color. After 5 minutes of shaking, the NMR tube was opened inside a glovebox and filtered through a glass microfiber filter. Benzene (200 µL) was added and the product crystallized at -30°C within 3 days. The crystals were of sufficient quality for X-ray analysis. Yield: 50 mg, 81.8 µmol, 70 %. $^{1}H$ NMR (600 MHz, THF-$d_8$, 25°C): δ = 1.73 (quin, 12H, THF), 3.57 (quin, 12H, THF), 5.86-5.89 (m, 3H, Ar), 6.01 (t, $^{3}J_{HH}$ = 7.0 Hz, 1H, Ar), 6.17-6.19 (m, 1H, Ar), 6.63-6.68 (m, 9H, Ar), 6.76-6.79 (t, $^{3}J_{HH}$ = 7.1 Hz, 1H, Ar), ppm. $^{13}C\{^{1}H\}$ APT NMR (151 MHz, THF-$d_8$, 56°C): δ = 25.1 (THF-$d_8$), 26.2 (THF), 67.2 (THF-$d_8$), 68.0 (THF), 92.7 ($C_0$), 111.1 (CH), 111.4 (CH), 116.9 (CH), 130.1 (CH), 130.6 (CH), 130.8 (CH), 143.9 ($C_0$), 158.8 ($C_0$) ppm. See Figures S72-S74.

**Hydrogenolysis of $[Ba(TPMI)(THF)_3]_2$ forming *N*-benzhydrylaniline.**

$[Ba(TPMI)(THF)_3]_2$ (10.0 mg, 16.4 µmol) was suspended in THF (0.5 mL) in a J. Young tube. The J. Young tube was connected to a $H_2$ gas manifold. The supply line was evacuated for 1 minute and subsequently purged with dry gas. This process was repeated three times using $N_2$ and three further times using $H_2$ to ensure the exclusion of air and moisture. The J. Young tube was pressurized with 1 bar of $H_2$ and efficient mixing of liquid and gas phases was achieved by rotation of the NMR tube for a period of 2 days. The deep red solution was decolorized and the formation of a white precipitate and quantitative conversion to $Ph_2C(H)$-N(H)Ph was observed. $^{1}H$ NMR (400 MHz, THF-$d_8$, 25°C): δ = 4.25 (br s, 1H, NH), 5.32 (s, 1H, CH(NH)), 5.90 (t, $^{3}J_{HH}$ = 7.0 Hz, 1H, *p*-CH NPh), 6.14 (d, $^{3}J_{HH}$ = 7.9 Hz, 2H, *o*-CH NPh), 6.73 (t, $^{3}J_{HH}$ = 7.7 Hz, 2H, *m*-CH NPh), 7.02 (t, $^{3}J_{HH}$ = 7.3 Hz, 2H, *p*-CH $NCHPh_2$), 7.15 (t, $^{3}J_{HH}$ = 7.6 Hz, 4H, *m*-CH $NCHPh_2$), 7.30 (d, $^{3}J_{HH}$ = 7.3 Hz, 4H, *o*-CH $NCHPh_2$) ppm. $^{13}C\{^{1}H\}$ APT NMR (101 MHz, THF-$d_8$, 56°C): δ = 25.1 (THF-$d_8$), 26.2 (THF), 67.2 (THF-$d_8$), 67.7 (CH(NH)) 68.0 (THF), 108.9 (CH),

112.8 (CH), 126.4 (CH), 128.6 (CH), 129.0 (CH), 130.0 (CH), 149.2 ($C_0$), 158.6 ($C_0$) ppm. (Figure S75-S76).

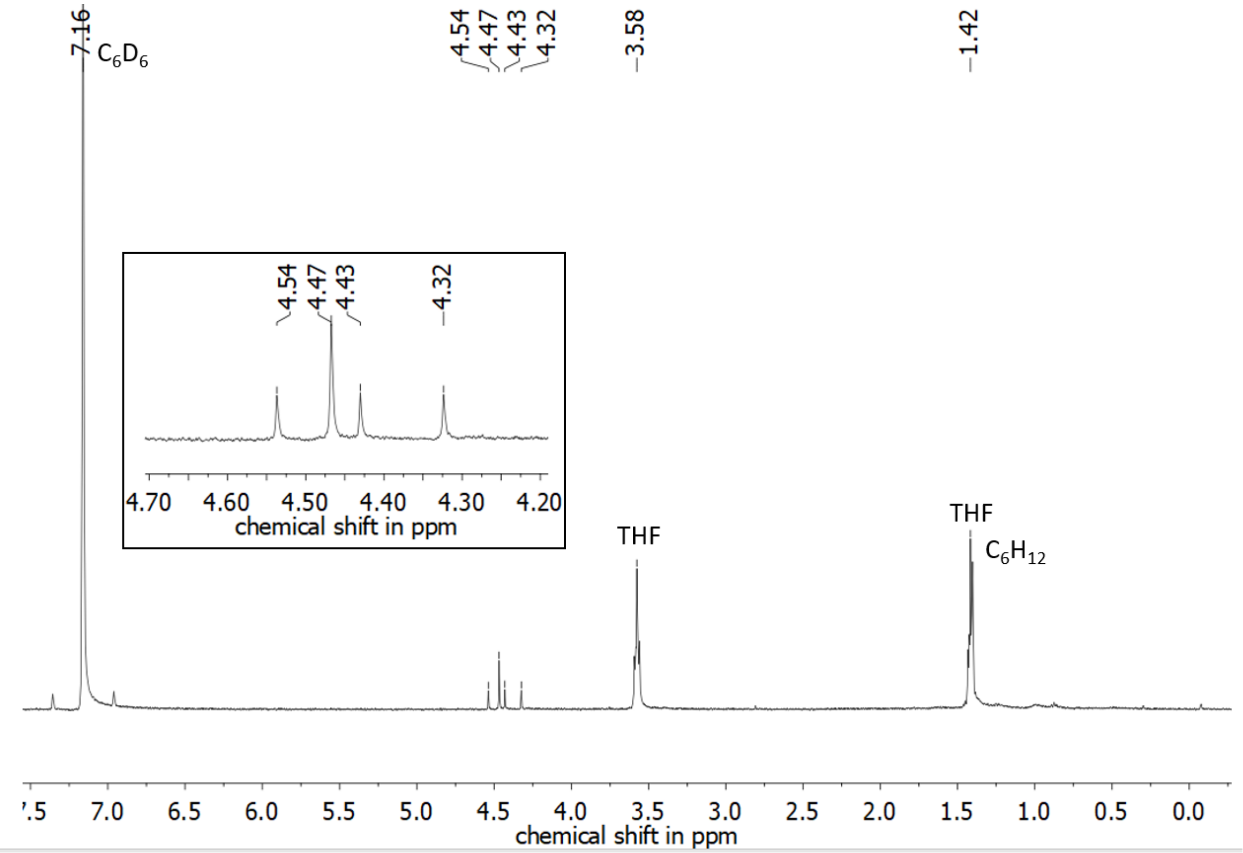


**Figure S62: $^{1}$H NMR spectrum (600 MHz, $C_6D_6$, 25°C) of the reaction of $BaH_2$ (formed at 120°C) and MeOD showing the characteristic signal pattern of H-D. The signal at 4.47 ppm is $H_2$ which is formed by H-D exchange between H-D and $BaH_2$.**

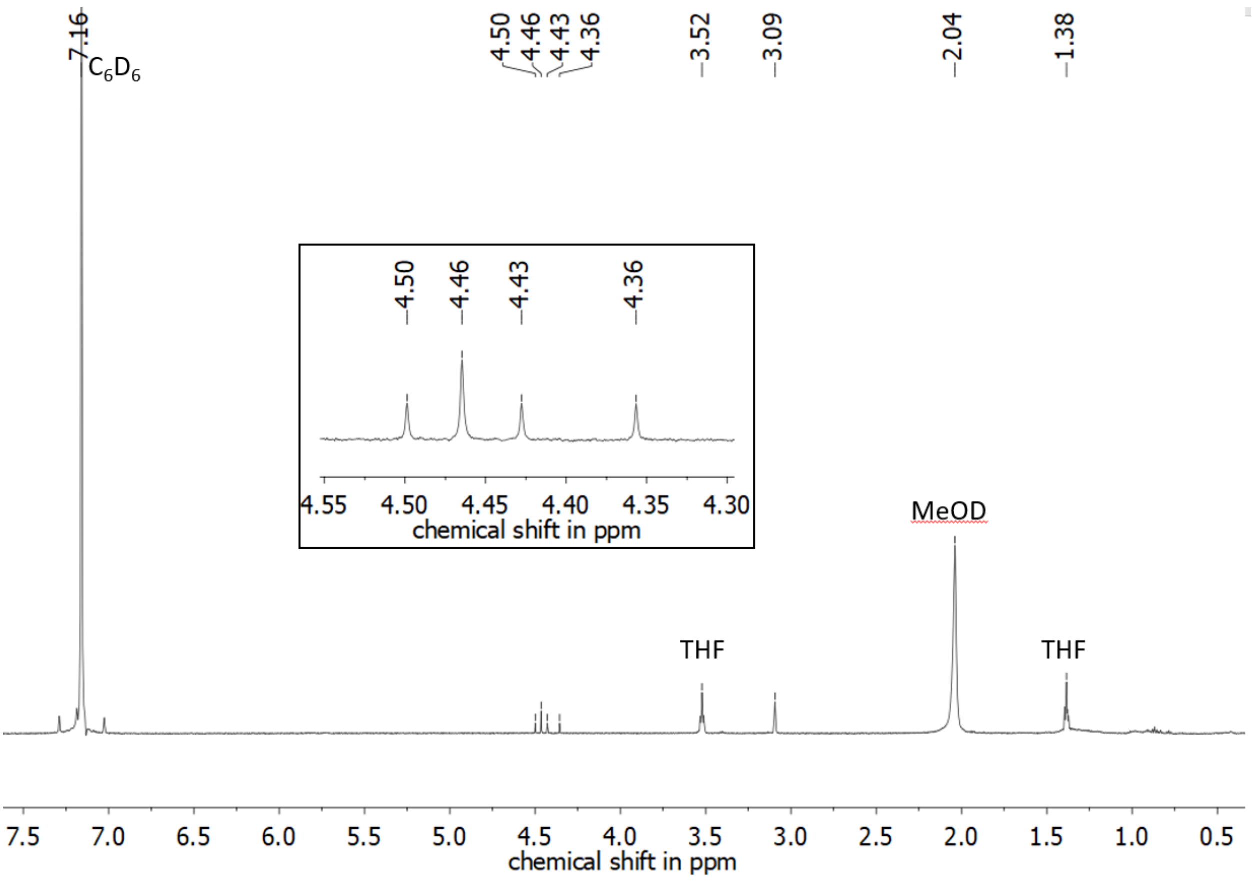


**Figure S63: $^1$H NMR spectrum (600 MHz, $C_6D_6$, 25°C) of the reaction of $BaH_2$ (formed at RT) and MeOD showing the characteristic signal pattern of H-D. The signal at 4.47 ppm is $H_2$ which is formed by H-D exchange between H-D and $BaH_2$.**

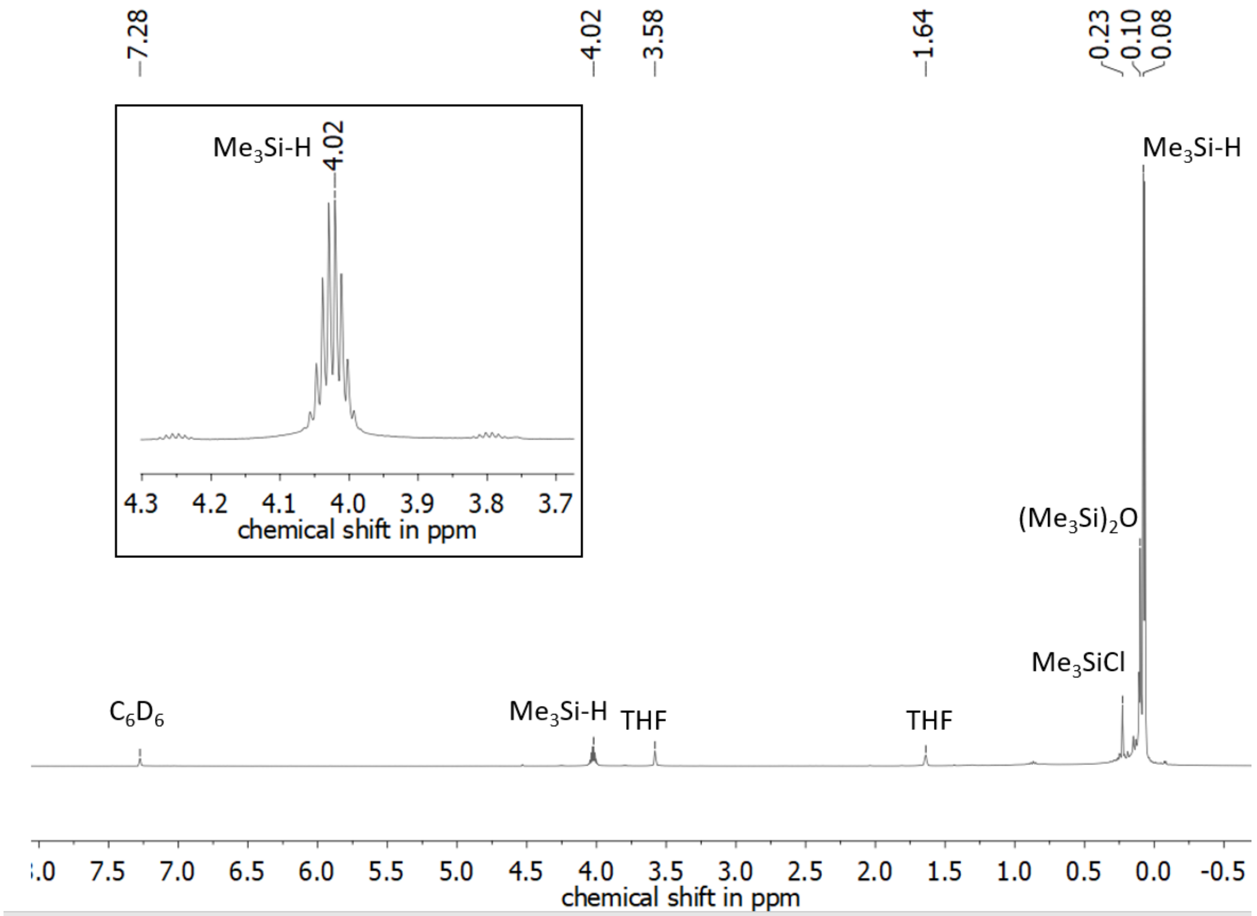


**Figure S64: $^1H$ NMR spectrum (600 MHz, THF-$d_8$/$C_6D_6$ (9/1) 25°C) of the reaction of $BaH_2$ and $Me_3SiCl$ showing the characteristic signals of $Me_3SiH$. $(Me_3Si)_2O$ is formed by reaction with traces of moisture over time.**

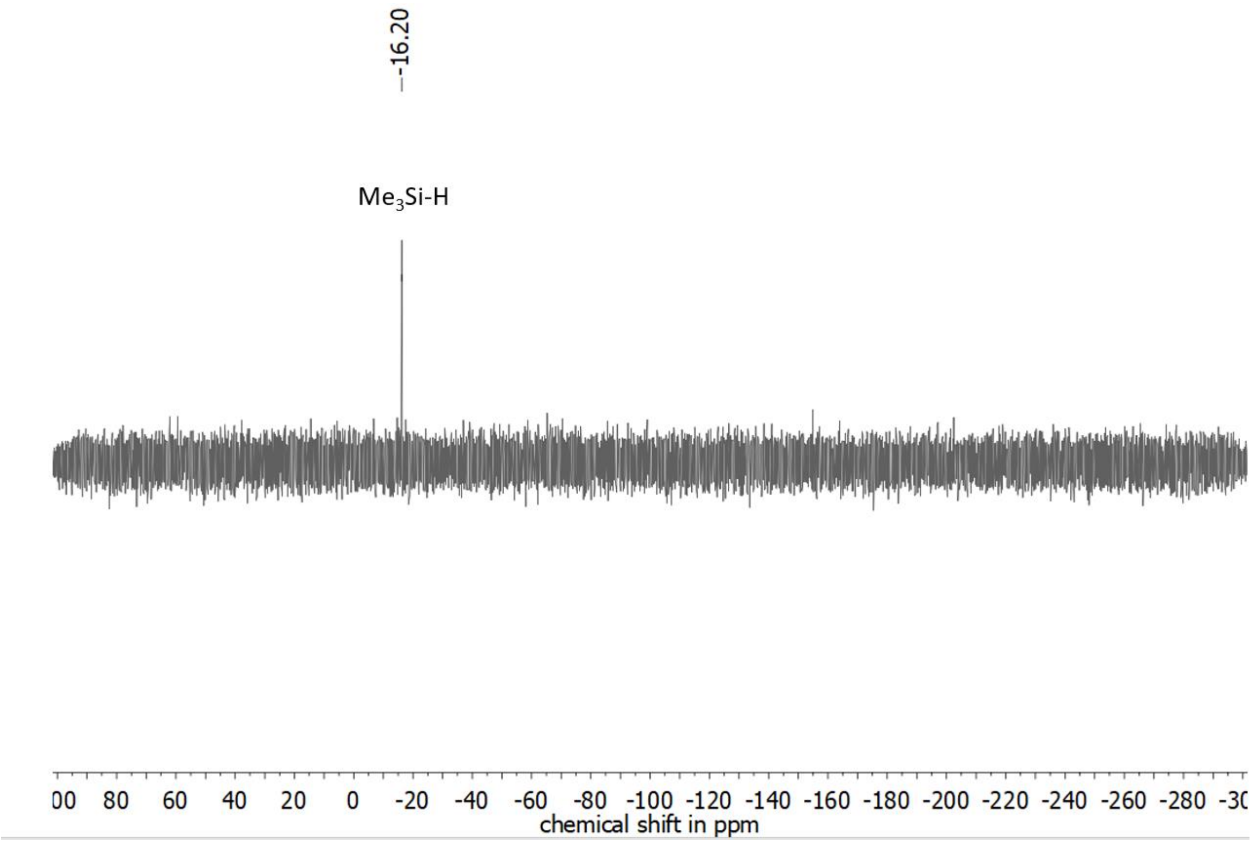


**Figure S65: $^{29}Si\{^1H\}$-NMR spectrum (119 MHz, THF-$d_8$ 25°C) of the reaction of $BaH_2$ and $Me_3SiCl$.**

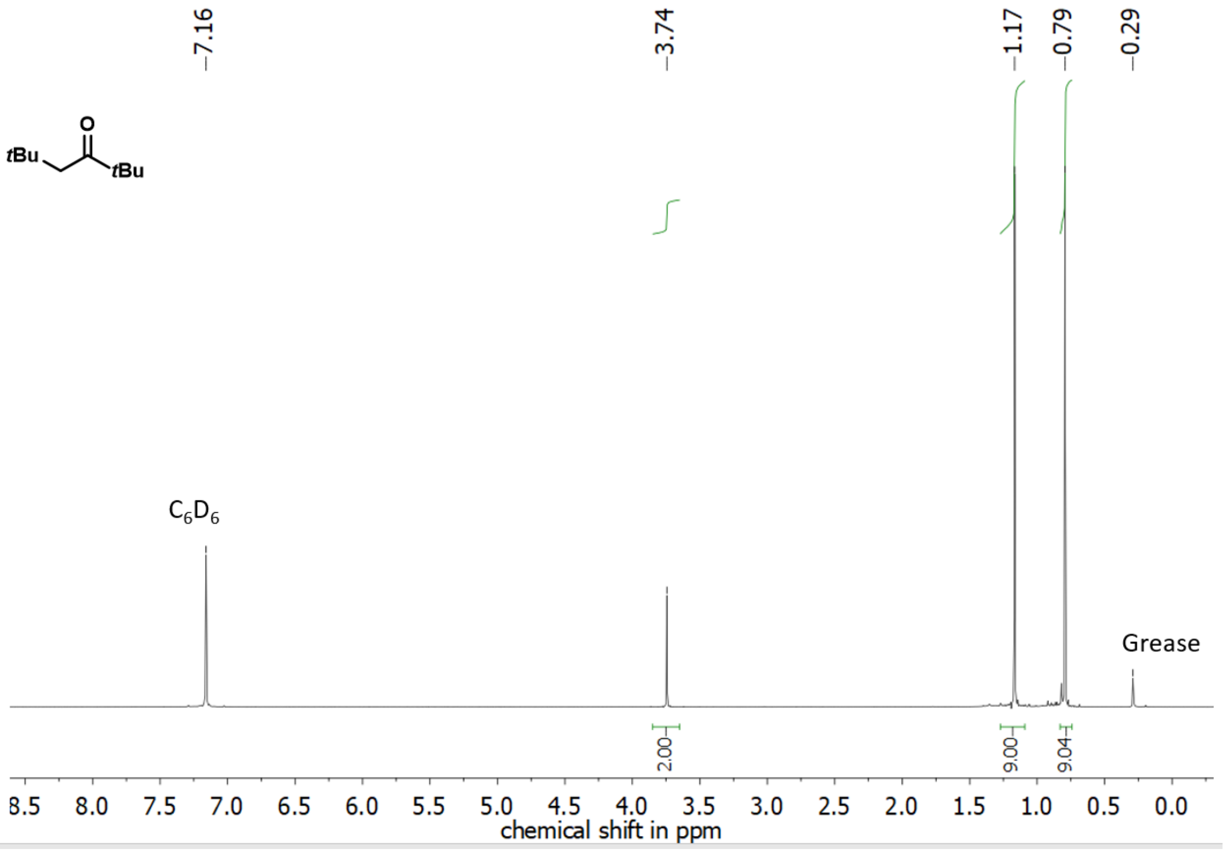


**Figure S66: $^{1}$H NMR spectrum (600 MHz, $C_6D_6$, 25°C) of the reaction of Ba(0)/$BaH_2$ and pivaldehyde, showing exclusively 2,2,5,5-tetramethyl-3-hexanone. This ketone was synthesized by elimination of BaO from the corresponding diol (2,2,5,5-tetramethylhexane-3,4-diol) formed by the reductive coupling of tBuCHO and Ba(0).**

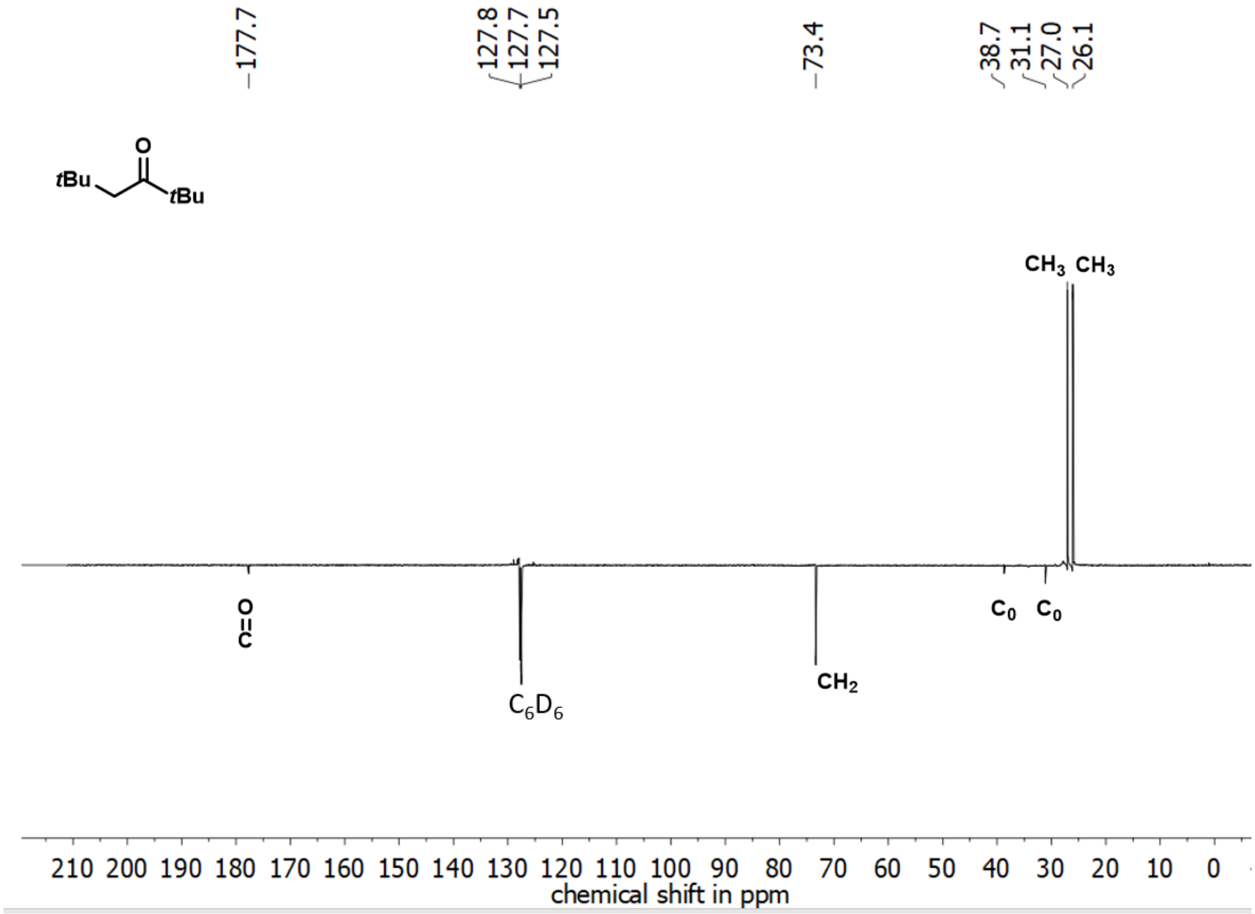


**Figure S67: $^{13}$C APT NMR spectrum (151 MHz, $C_6D_6$, 25°C) of the reaction of Ba(0)/$BaH_2$ and pivaldehyde, showing exclusively 2,2,5,5-tetramethyl-3-hexanone. This ketone was synthesized by elimination of BaO from the corresponding diol (2,2,5,5-tetramethylhexane-3,4-diol) formed by the reductive coupling of tBuCHO and Ba(0).**

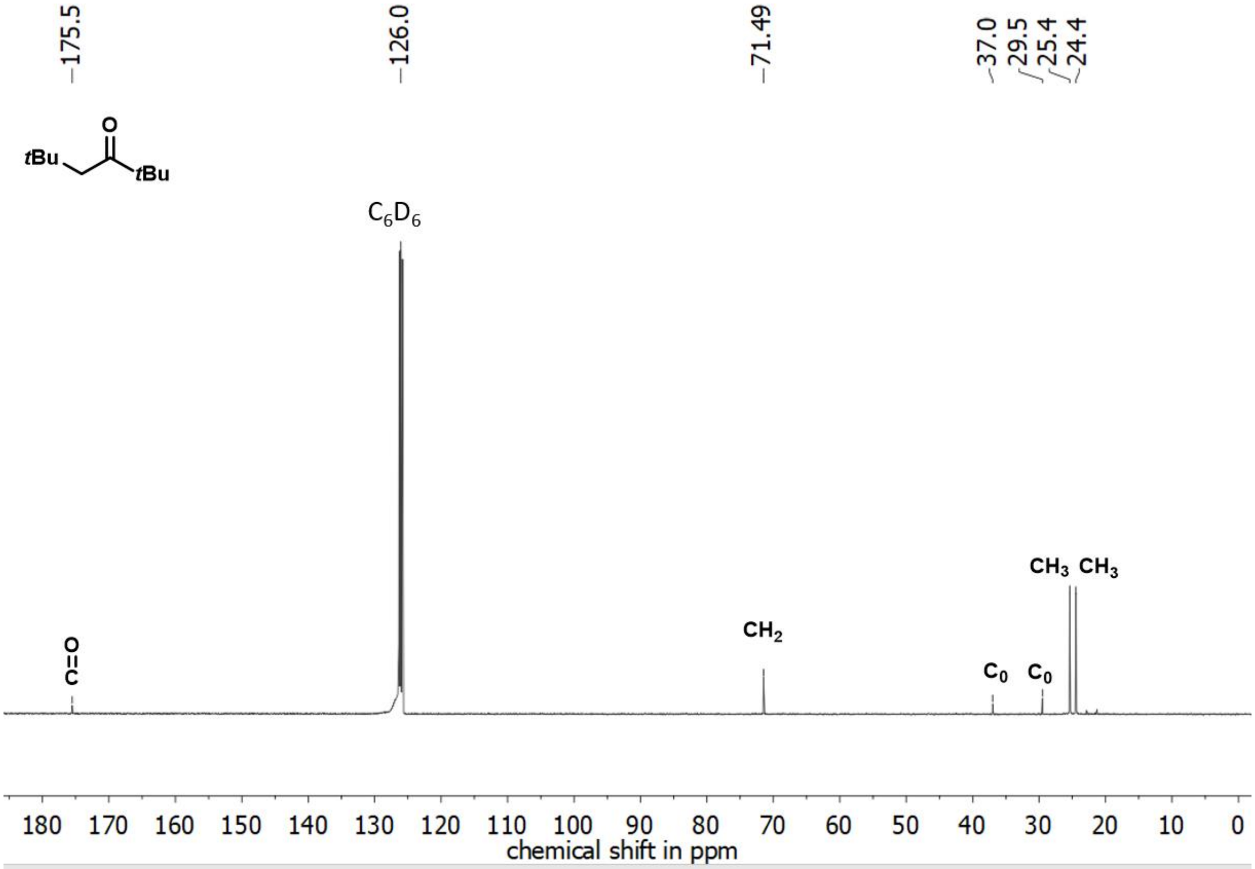


**Figure S68: $^{13}C$ NMR spectrum (101 MHz, $C_6D_6$, 25°C) of the reaction of Ba(0)/$BaH_2$ and pivaldehyde, showing exclusively 2,2,5,5-tetramethyl-3-hexanone. This ketone was synthesized by elimination of BaO from the corresponding diol (2,2,5,5-tetramethylhexane-3,4-diol) formed by the reductive coupling of tBuCHO and Ba(0).**

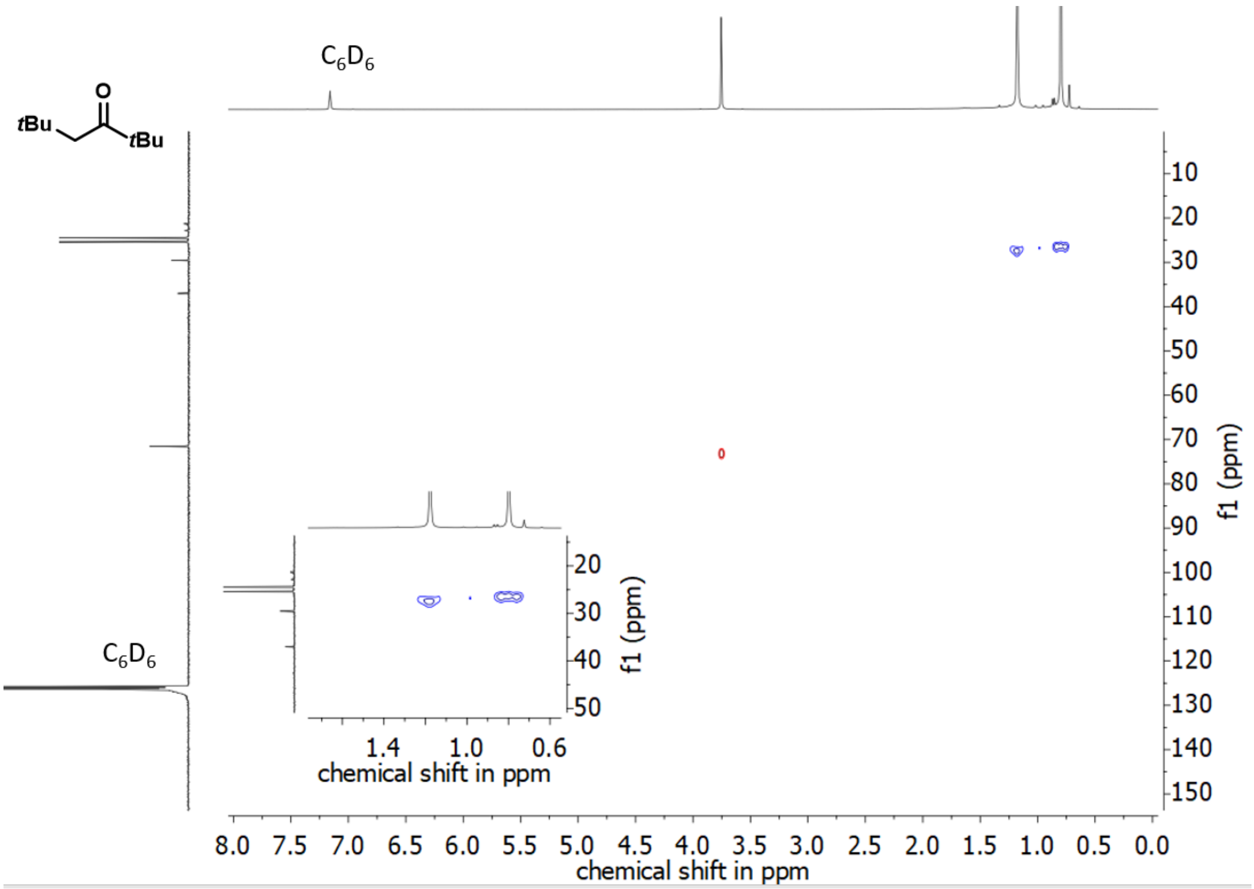


**Figure S69: $^{1}H$ $^{13}C$ HSQC spectrum (101 MHz, $C_6D_6$, 25°C) of the reaction of Ba(0)/$BaH_2$ and pivaldehyde, showing exclusively 2,2,5,5-tetramethyl-3-hexanone. This ketone was synthesized by elimination of BaO from the corresponding diol (2,2,5,5-tetramethylhexane-3,4-diol) formed by the reductive coupling of tBuCHO and Ba(0).**

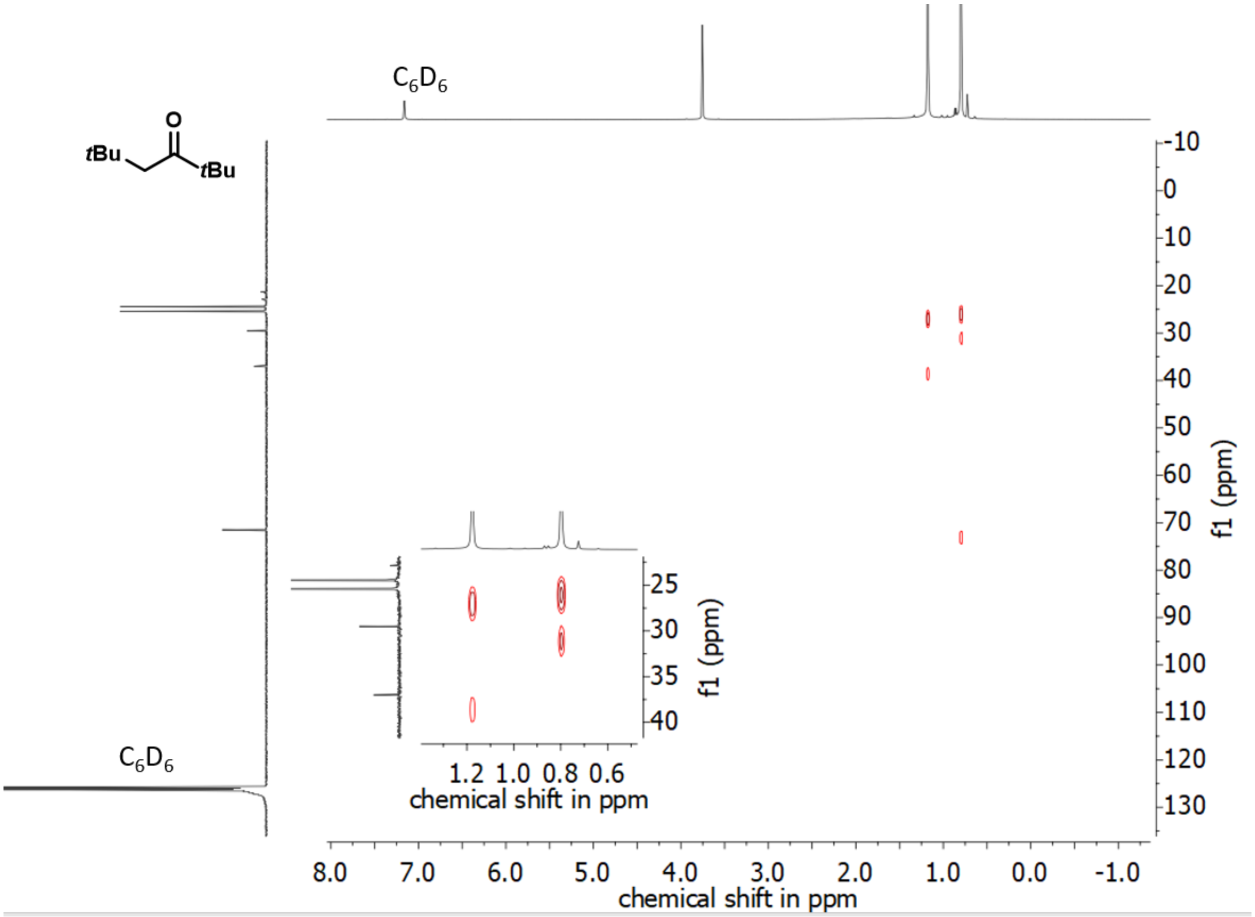


**Figure S70: $^1$H $^{13}$C-HMBC spectrum (101 MHz, $C_6D_6$, 25°C) of the reaction of Ba(0)/$BaH_2$ and pivaldehyde, showing exclusively 2,2,5,5-tetramethyl-3-hexanone. This ketone was synthesized by elimination of BaO from the corresponding diol (2,2,5,5-tetramethylhexane-3,4-diol) formed by the reductive coupling of tBuCHO and Ba(0).**

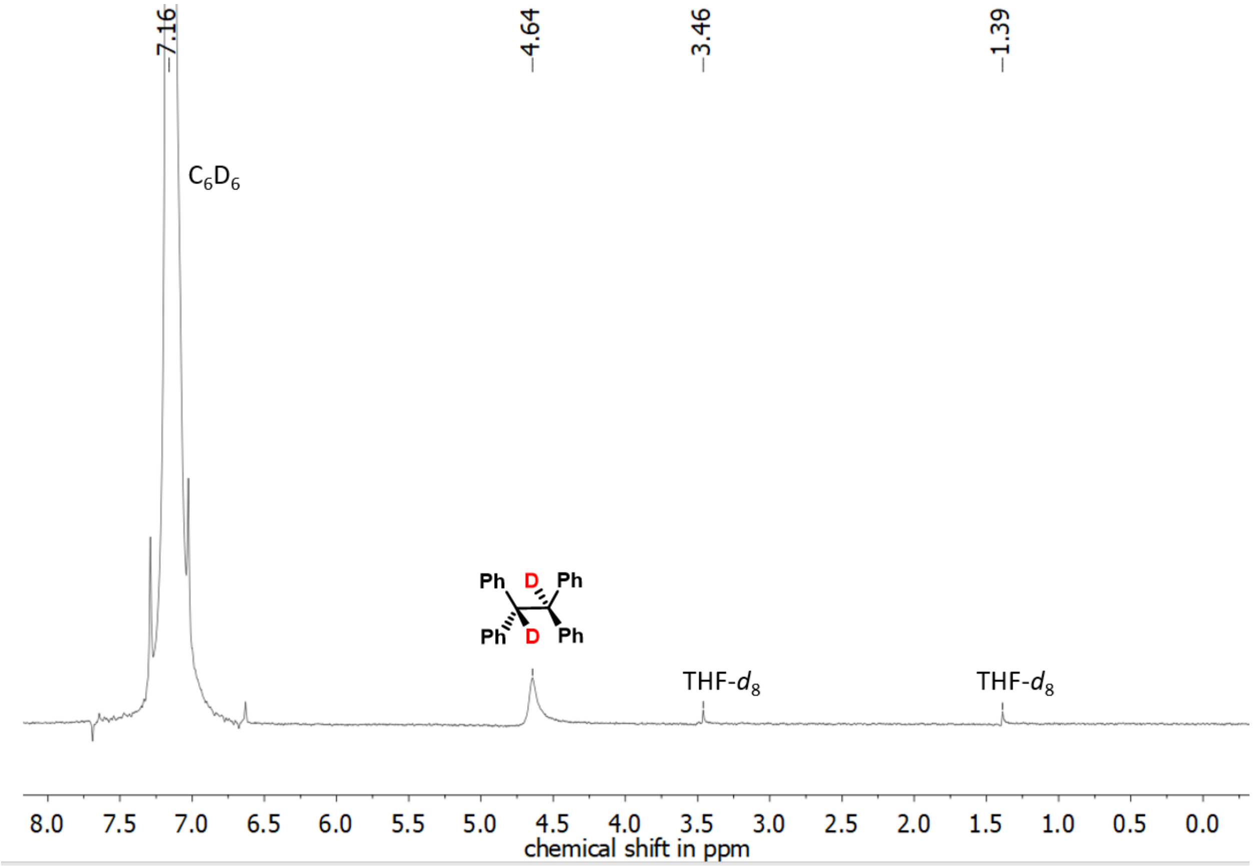


**Figure S71: $^{2}D$ NMR spectrum (600 MHz, $C_6D_6$, 25°C) of the reaction of Ba(0) and tetraphenylethylene after quenching with MeOD showing formation of $Ph_2C(D)$-$(D)CPh_2$.**

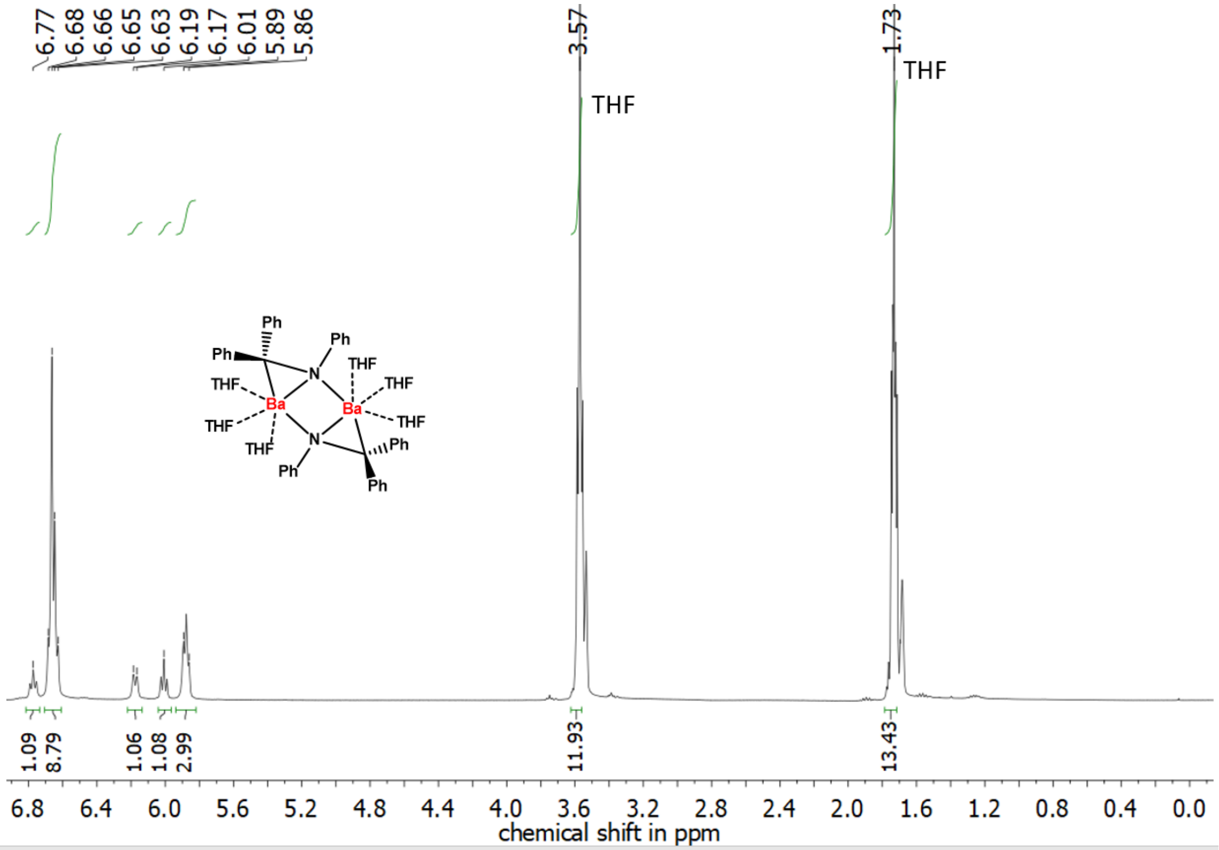


**Figure S72: $^{1}$H NMR spectrum (400 MHz, THF-$d_8$, 25°C) of [Ba(TPMA)(THF)$_3$]$_2$.**

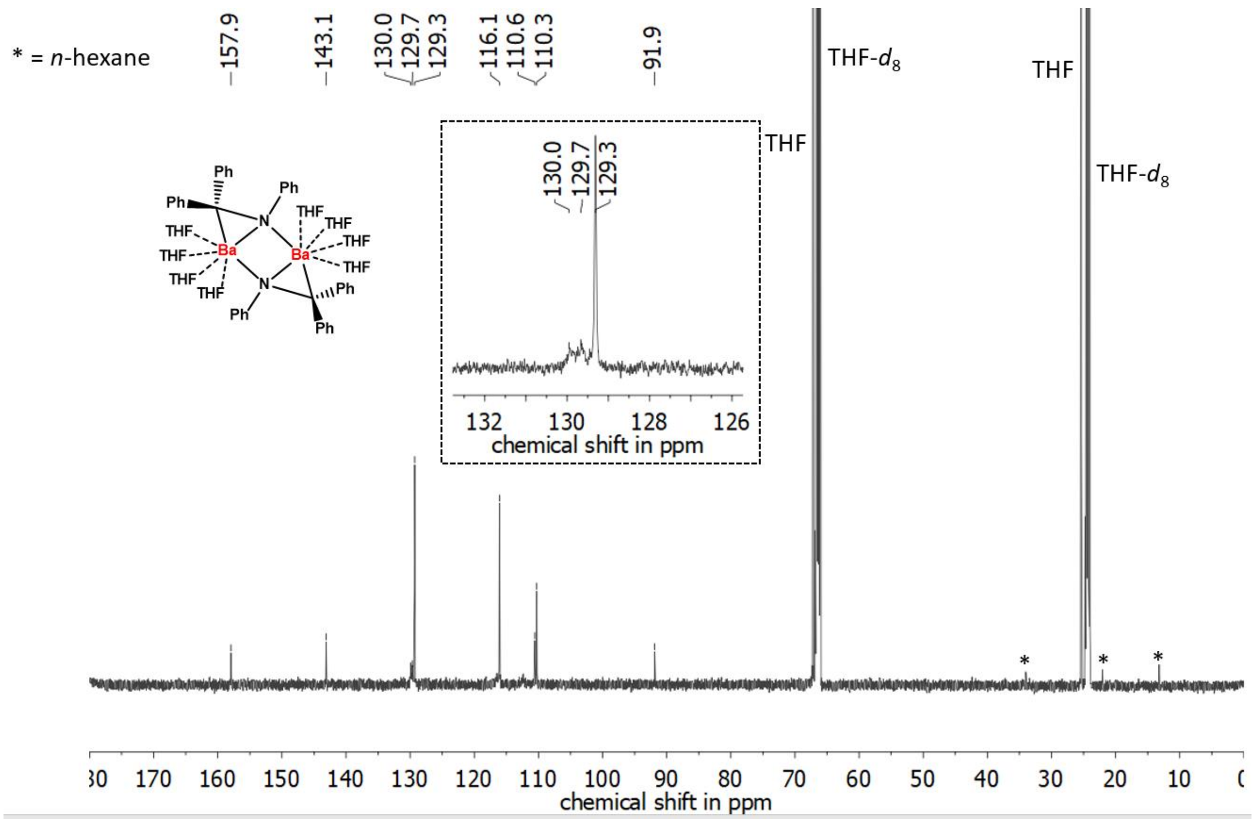


**Figure S73: $^{13}$C NMR spectrum (151 MHz, THF-$d_8$, 55°C) of $[Ba(TPMA)(THF)_3]_2$. The broad signals (130.6 ppm, 130.8 ppm) are likely related to molecular dynamics. Due to the low solubility characterization in other solvents was not possible.**

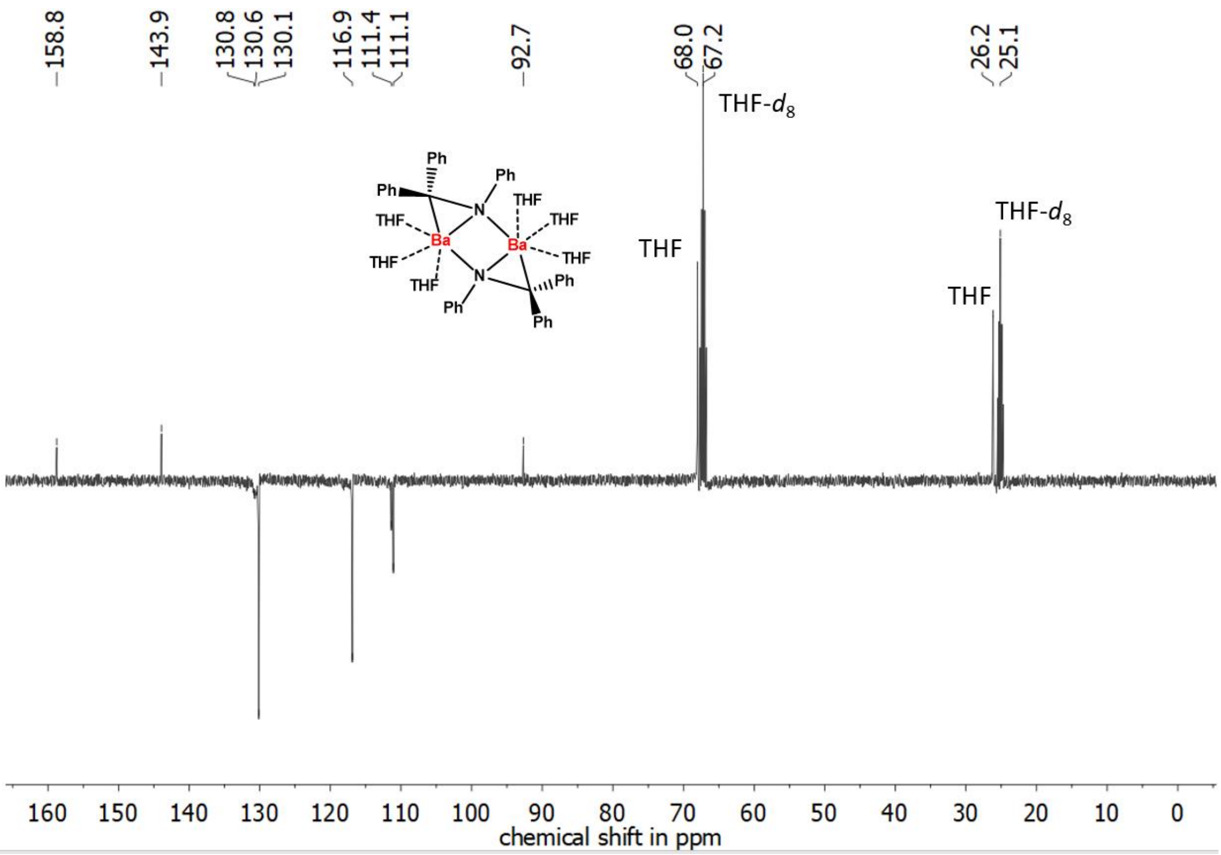


**Figure S74: $^{13}C$ APT NMR spectrum (101 MHz, THF-$d_8$, 55°C) of $[Ba(TPMA)(THF)_3]_2$. The broad signals (130.6 ppm, 130.8 ppm) are likely related to molecular dynamics. Due to the low solubility characterization in other solvents was not possible.**

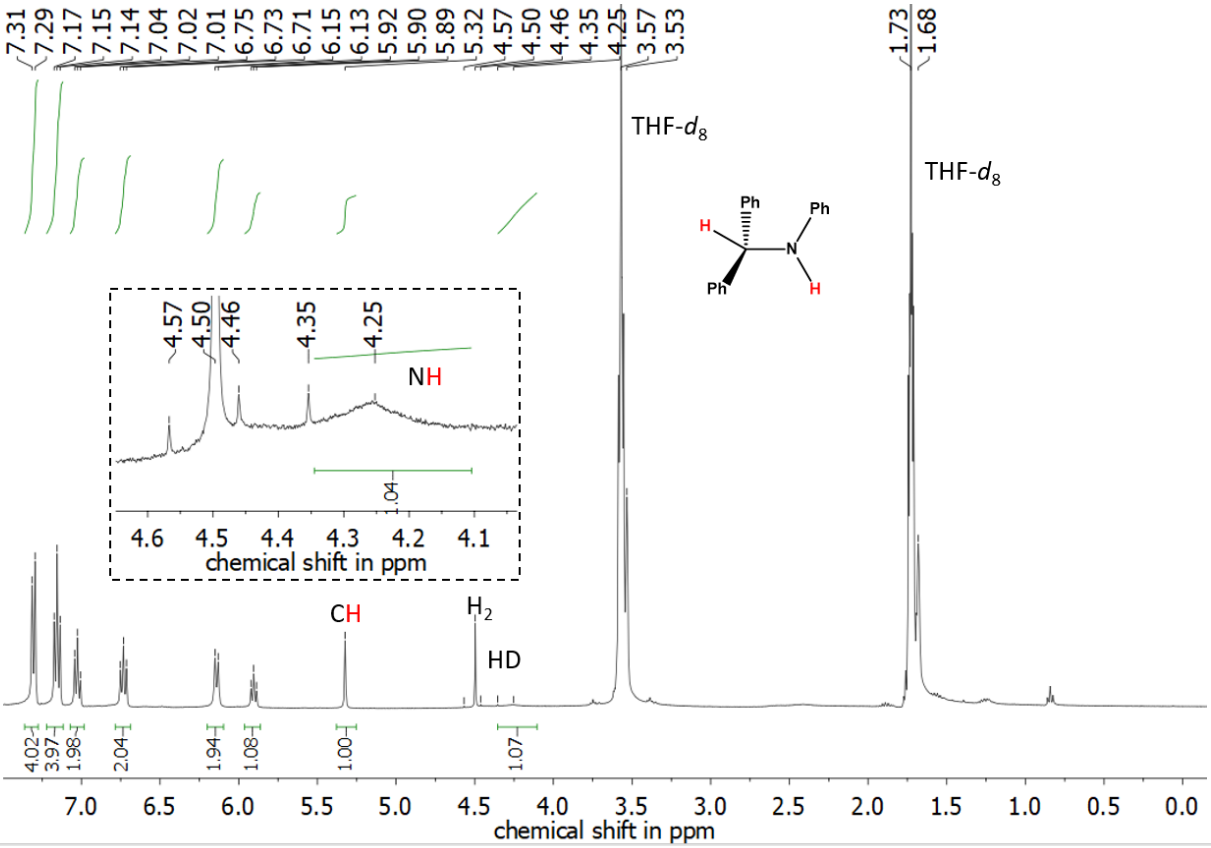


**Figure S75: $^{1}$H NMR spectrum (600 MHz, THF-*d*$_8$, 25°C) after hydrogenolysis of [Ba(TPMA)(THF)$_3$]$_2$ forming *N*-benzhydrylaniline. The formation of H-D likely results from H-D exchange with the deuterated solvent.**

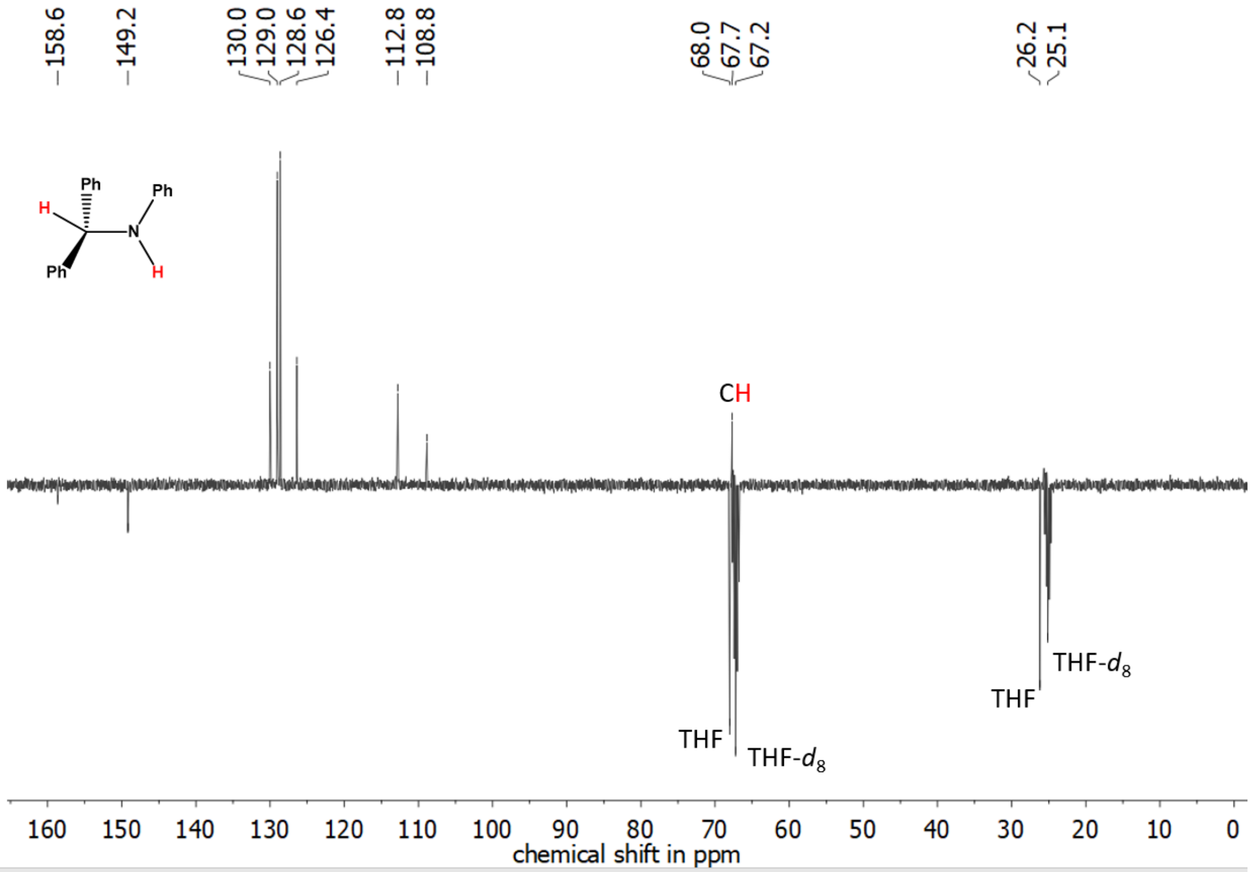


**Figure S76: $^{13}$C APT NMR spectrum (151 MHz, THF-$d_8$, 25°C) after hydrogenolysis of $[Ba(TPMA)(THF)_3]_2$ forming *N*-benzhydrylaniline.**

## 1.7 Crystal Structure Determination

### Structure determination of $[Ba(TPMA)(THF)_3]_2$

A red crystal of compound $[Ba(TPMA)(THF)_3]_2$ was embedded in inert perfluoropolyalkylether (viscosity 1800 cSt; ABCR GmbH) and mounted using a Hampton Research CryoLoop. The crystal was then flash cooled to 100.01(10) K in a nitrogen gas stream and kept at this temperature during the experiment. The crystal structure was measured on a SuperNova diffractometer with Atlas S2 detector using a CuKα microfocus source. The measured data was processed with the CrysAlisPro (v40.53) software package.[S7] Using Olex2 [S8], the structure was solved with the ShelXT [S9] structure solution program using Intrinsic Phasing and refined with the ShelXL [S10] refinement package using Least Squares minimization. All non-hydrogen atoms were refined anisotropically.

Disorder of two THF ligands coordinated to the barium metal atom was observed, and was modeled with the help of similarity restraints (SIMU, SADI) and a rigid bond restraint (RIGU)[S11]. The relative occupancies of the two alternative orientations were refined to 0.605(15)/0.395(15) and 0.54(3)/0.46(3), respectively.

One benzene and one THF molecule were found superimposed on the same position and were modeled with the help of similarity restraints (SIMU, SADI) and a rigid bond restraint (RIGU)[S11]. The relative occupancies of the two molecules were refined to 0.75 for benzene and 0.25 for the THF molecule.

The crystal structure data has been deposited with the Cambridge Crystallographic Data Centre. CCDC 2039004 contains the supplementary crystallographic data for complex $[Ba(TPMA)(THF)_3]_2$.

This data can be obtained free of charge from The Cambridge Crystallographic Data Centre via www.ccdc.cam.ac.uk/data_request/cif.

Crystallographic and refinement data are summarized in Table S3.

Table S3: Crystal data and structure refinement for $[Ba(TPMA)(THF)_3]_2$.

| | |
|---|---|
| **Identification code** | hasj200915a |
| **Empirical formula** | $C_{85}H_{103}Ba_2N_2O_{6.5}$ |
| **Formula weight** | 1531.37 |
| **Temperature/K** | 100.01(10) |
| **Crystal system** | triclinic |
| **Space group** | P-1 |
| **a/Å** | 11.2852(5) |
| **b/Å** | 11.5202(7) |
| **c/Å** | 15.3234(5) |
| **α/°** | 83.437(4) |
| **β/°** | 86.489(3) |
| **γ/°** | 69.297(5) |
| **Volume/Å$^3$** | 1850.84(16) |
| **Z** | 1 |
| **$\rho_{calc}$g/cm$^3$** | 1.374 |
| **μ/mm$^{-1}$** | 8.646 |
| **F(000)** | 791.0 |
| **Crystal size/mm$^3$** | 0.097 × 0.074 × 0.054 |
| **Radiation** | Cu Kα (λ = 1.54184) |
| **2Θ range for data collection/°** | 5.808 to 145.17 |
| **Index ranges** | -13 ≤ h ≤ 13, -14 ≤ k ≤ 13, -18 ≤ l ≤ 18 |
| **Reflections collected** | 35101 |
| **Independent reflections** | 7221 [Rint = 0.0474, Rsigma = 0.0359] |
| **Data/restraints/parameters** | 7221/280/508 |
| **Goodness-of-fit on F$^2$** | 1.036 |
| **Final R indexes [I>=2σ (I)]** | $R_1$ = 0.0277, $wR_2$ = 0.0684 |
| **Final R indexes [all data]** | $R_1$ = 0.0297, $wR_2$ = 0.0698 |
| **Largest diff. peak/hole / e Å$^{-3}$** | 0.58/-1.01 |
| **CCDC number** | 2039004 |

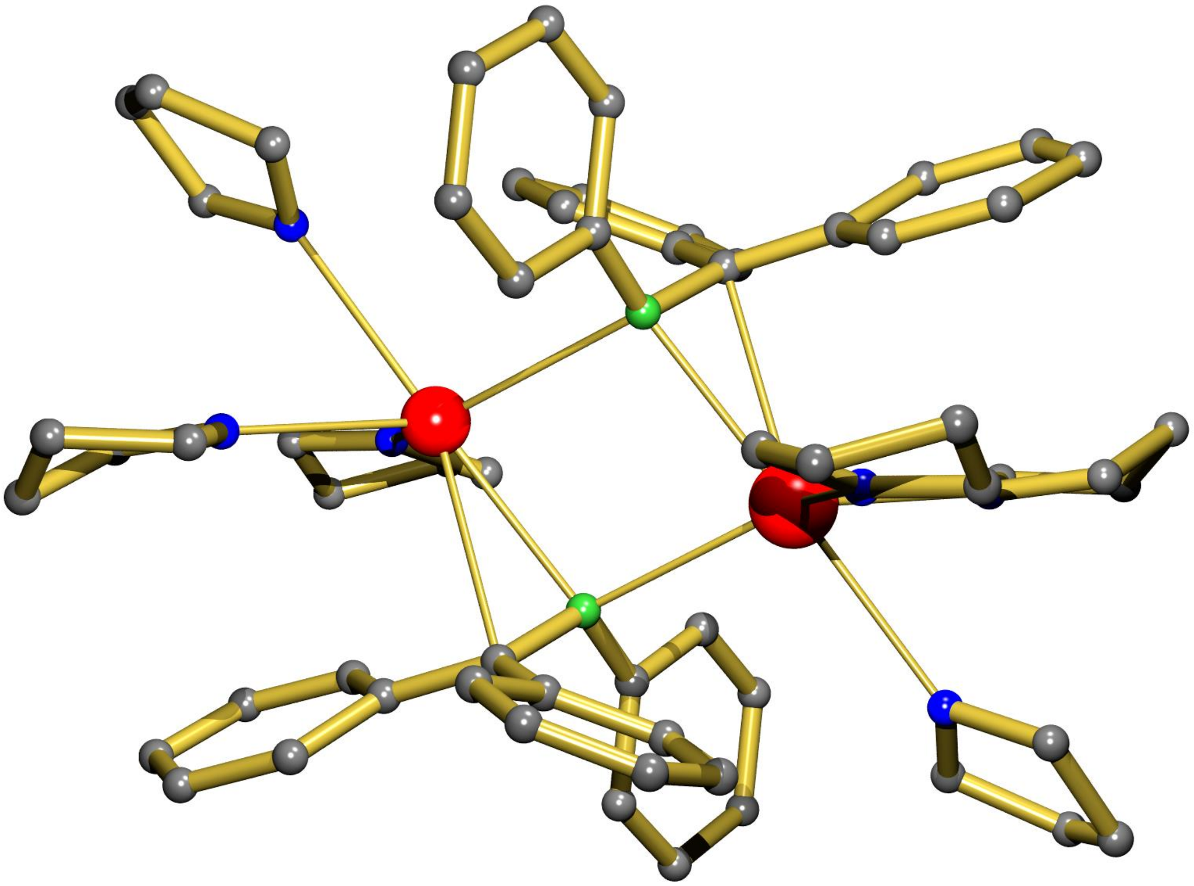

**Figure S77: Crystal Structure of [Ba(TPMA)(THF)$_3$]$_2$.**

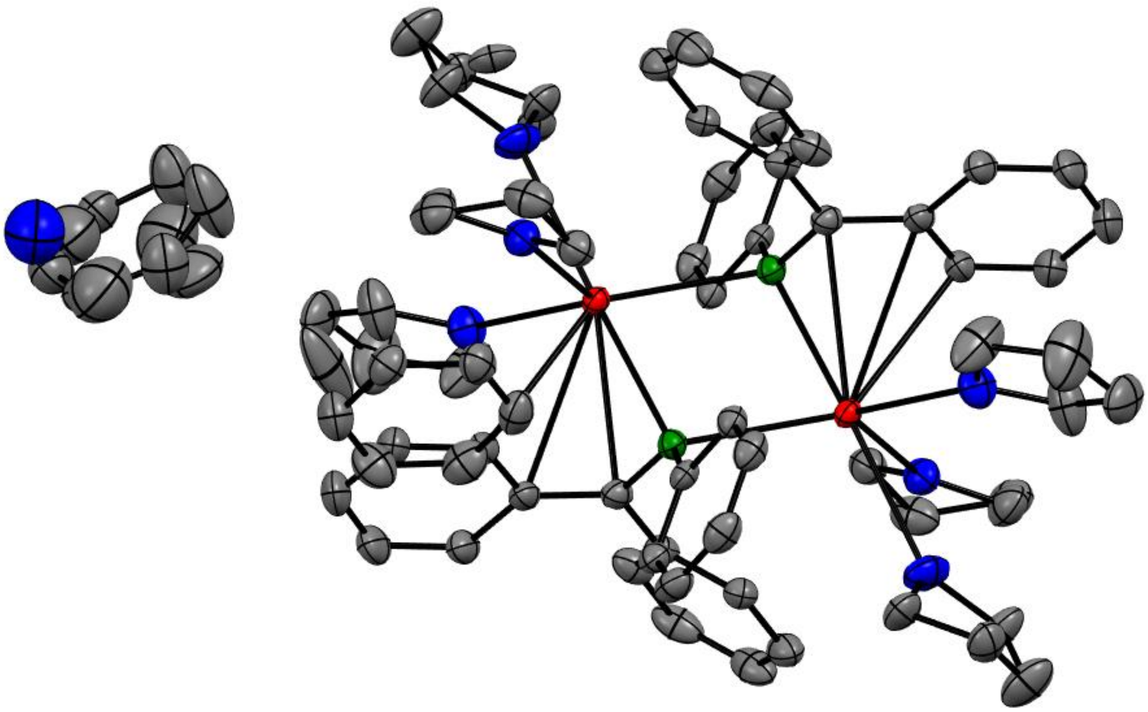

**Figure S78: ORTEP plot of [Ba(TPMA)(THF)$_3$]$_2$·(THF)/(C$_6$H$_6$). Ellipsoids are at 50% probability.**

## 1.8 Temperature and Pressure Dependency

### General procedure for examining the temperature and pressure dependency

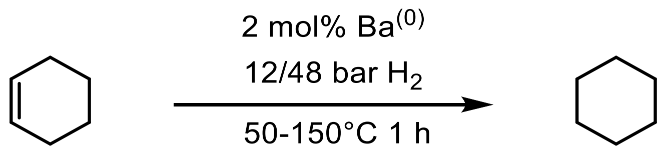


**Figure S79: Catalytic hydrogenation of cyclohexene.**

An oven-dried miniature stainless steel autoclave (15 mL) containing a magnetic stir bar was charged with activated Ba(0) (0.1 mmol, 13.7 mg) and cyclohexene (5.0 mmol, 0.51 mL) in an $N_2$-filled glovebox. The tightly sealed pressure vessel was transferred out of the glovebox and connected to a $H_2$ gas manifold. The supply line was evacuated for 1 minute and subsequently purged with dry gas. This process was repeated for three times using $N_2$ and for three further times using $H_2$ to ensure the exclusion of air and moisture. The reactor was then pressurized with $H_2$ (12 bar or 48 bar) and heated to the desired temperature (50 °C, 80 °C, 100 °C, 130 °C or 150 °C) in a pre-heated aluminum metal block. After stirring the reaction mixture for 1 h, the autoclave was cooled to room temperature in a water bath and subsequently vented. A 100 µL aliquot of the product mixture was diluted with $C_6D_6$ (500 µL), filtered through a glass microfiber filter in a Pasteur pipette and analyzed by $^1$H NMR spectroscopy. Catalytic conversion was determined by integration of characteristic $^1$H NMR resonances versus the olefin signal at 5.68 ppm.

**Table S4.** Catalytic cyclohexene hydrogenation; reactions performed neat (2 mol%, 1 h).

| $H_2$ [bar] | T [°C] | Conv. [%] | TOF [$h^{-1}$] |
|---|---|---|---|
| 12 | 50 | 13 | 6.5 |
| 12 | 80 | 53 | 26.5 |
| 12 | 100 | 57 | 28.5 |
| 12 | 130 | 60 | 30.0 |
| 12 | 150 | 68 | 34.0 |
| 48 | 50 | 19 | 9.5 |
| 48 | 80 | 34 | 17.0 |
| 48 | 100 | 55 | 27.5 |
| 48 | 130 | 80 | 40.0 |
| 48 | 150 | 90 | 45.0 |

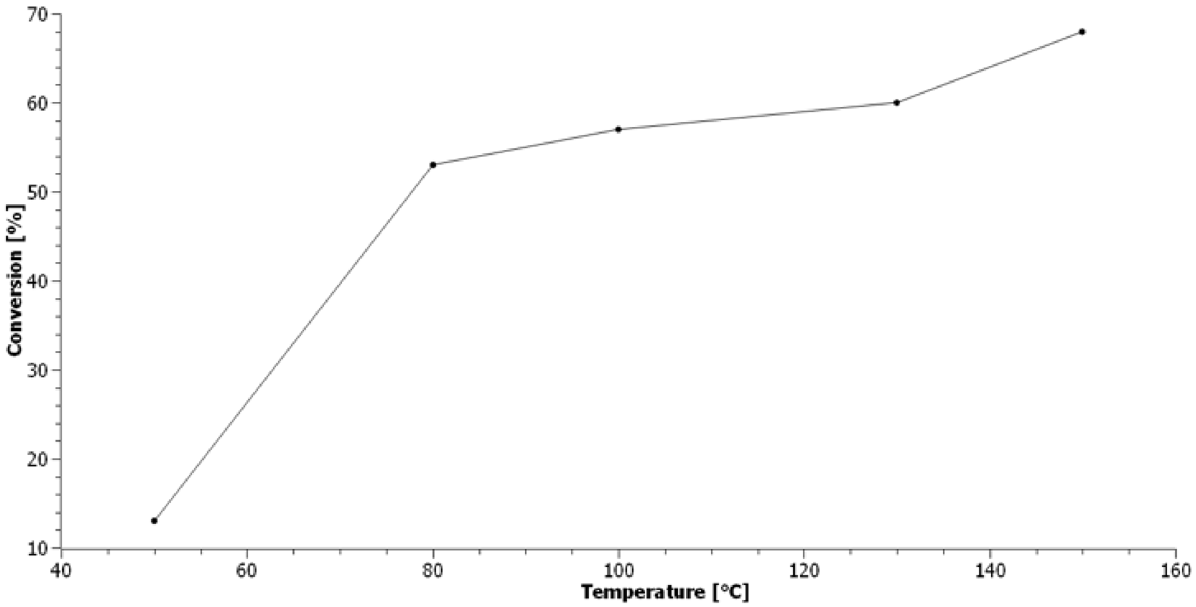


**Figure S80: Temperature dependent conversions in the hydrogenation of cyclohexene using 12 bar of $H_2$.**

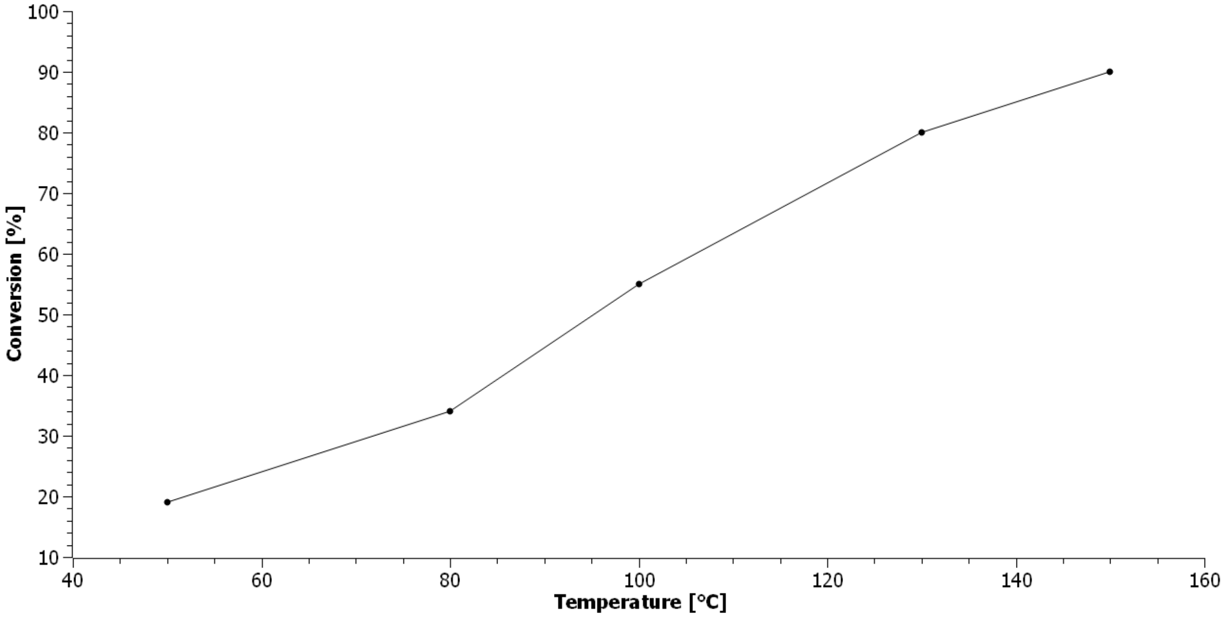


**Figure S81: Temperature dependent conversions in the hydrogenation of cyclohexene using 48 bar of $H_2$.**

## 1.9 Ba activation using ammonia

Activation of Ba metal with liquid ammonia has been investigated. The metal was dissolved in dry liquid ammonia resulting in the typical blue color related to solvated electrons. Evaporation of ammonia was expected to give highly activated Ba metal. It was found that evaporation of ammonia at room temperature led to major formation of $Ba(NH_2)_2$.[S12] However, in case the ammonia was removed at lower temperatures, $Ba(NH_2)_2$ formation could be partially prevented.

### Synthesis of barium amide

Ammonia (N38, approx. 100 mL) was condensed into a Schlenk flask charged with barium chunks (5.06 g, 36.8 mmol). Ammonia was evaporated from the dark blue solution under a continuous stream of dry nitrogen over night at room temperature. The resulting off-white solid was dried under reduced pressure at room temperature to yield $Ba(NH_2)_2$. Yield: 5.78 g, 34.1 mmol, 93 %.

### Synthesis of finely divided Ba(0) powder using ammonia

Ammonia (N38, approx. 100 mL) was condensed into a Schlenk flask charged with barium chunks (5.56 g, 40.5 mmol) while cooling with a dry ice/acetone bath. After the metal had been fully dissolved, the dark blue solution was dried under reduced pressure while cooling to -78 °C was continued. The golden precipitate converted slowly into a grey foam, which liquefied into a dark blue solution upon warming to circa -40 °C. The cooling bath was removed and remaining ammonia (circa 20 mL) was pumped off under high vacuum while thawing. After thorough drying at room temperature, a grey metallic powder was obtained. Yield: 5.79 g. Assuming $Ba(NH_2)_2$ as the only contaminant, this corresponds to a Ba metal content of 82%.

## Catalytic activity of $Ba(NH_2)_2$ and Ba(0) activated by dissolution in liquid ammonia

An oven-dried miniature stainless steel autoclave (15mL) containing a magnetic stir bar was charged with $Ba(NH_2)_2$ (42.3 mg, 0.25 mmol) or Ba(0) (34.3 mg, 0.25 mmol) in an $N_2$-filled glovebox. Either cyclohexene (0.51 ml, 5.0 mmol) or benzene (0.45 ml, 5.0 mmol) was added and the tightly sealed pressure vessel was transferred out of the glovebox and connected to a $H_2$ gas manifold. The supply line was evacuated for 1 minute and subsequently purged with dry gas. This process was repeated for three times using $N_2$ and for three further times using $H_2$ to ensure the exclusion of air and moisture. The reactor was then pressurized with 12 bar $H_2$ (cyclohexene) or 48 bar $H_2$ (benzene) and heated to the desired temperature of 120°C (cyclohexene) or 150°C (benzene) in a pre-heated aluminum metal block. After stirring the reaction mixture for 2 h (cyclohexene) or 24h (benzene), the autoclave was cooled to room temperature in a water bath and subsequently vented. A 200 μL aliquot of the product mixture was diluted with THF-$d_8$ (300 μL), filtered through a glass microfiber filter in a Pasteur pipette and analyzed by $^1H$ NMR spectroscopy. The conversion of substrates was estimated via integration of characteristic $^1H$ NMR resonances.

**Table S5.** Catalytic cyclohexene and benzene hydrogenation; reactions performed neat.

| Entry | Catalyst | Substrate | mol% | $H_2$ [bar] | T [°C] | t [h] | Conv. [%] |
|---|---|---|---|---|---|---|---|
| **1** | $Ba(NH_2)_2$ | 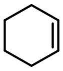 | 5 | 12 | 120 | 2 | 0 |
| **2** | Ba(0) $NH_3$ activ. | 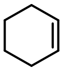 | 5 | 12 | 120 | 2 | 8 |
| **3** | Ba(0) metal vapor | 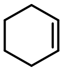 | 2 | 12 | 120 | 2 | 99 |
| **4** | $Ba(NH_2)_2$ | 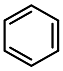 | 5 | 50 | 150 | 24 | 0 |
| **5** | Ba(0) $NH_3$ activ. | 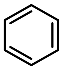 | 5 | 50 | 150 | 24 | 0 |
| **6** | Ba(0) metal vapor | 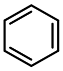 | 5 | 50 | 150 | 24 | 25 |

[a] = synthesized by dissolution in ammonia, [b] = synthesized by metal vapor synthesis

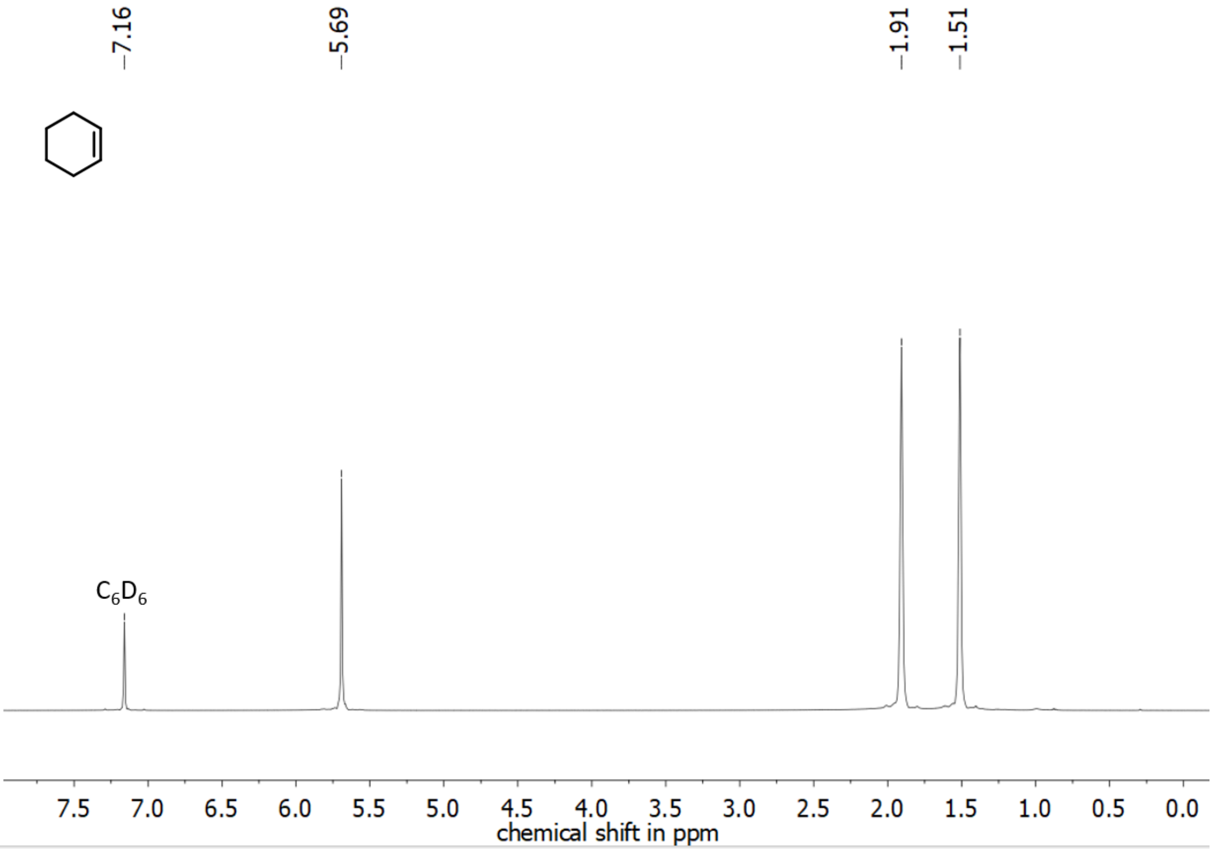


**Figure S82: Attempted hydrogenation of cyclohexene (neat) using 5 mol% $Ba(NH_2)_2$ and 12 bars of $H_2$ at 120°C (2 h).**

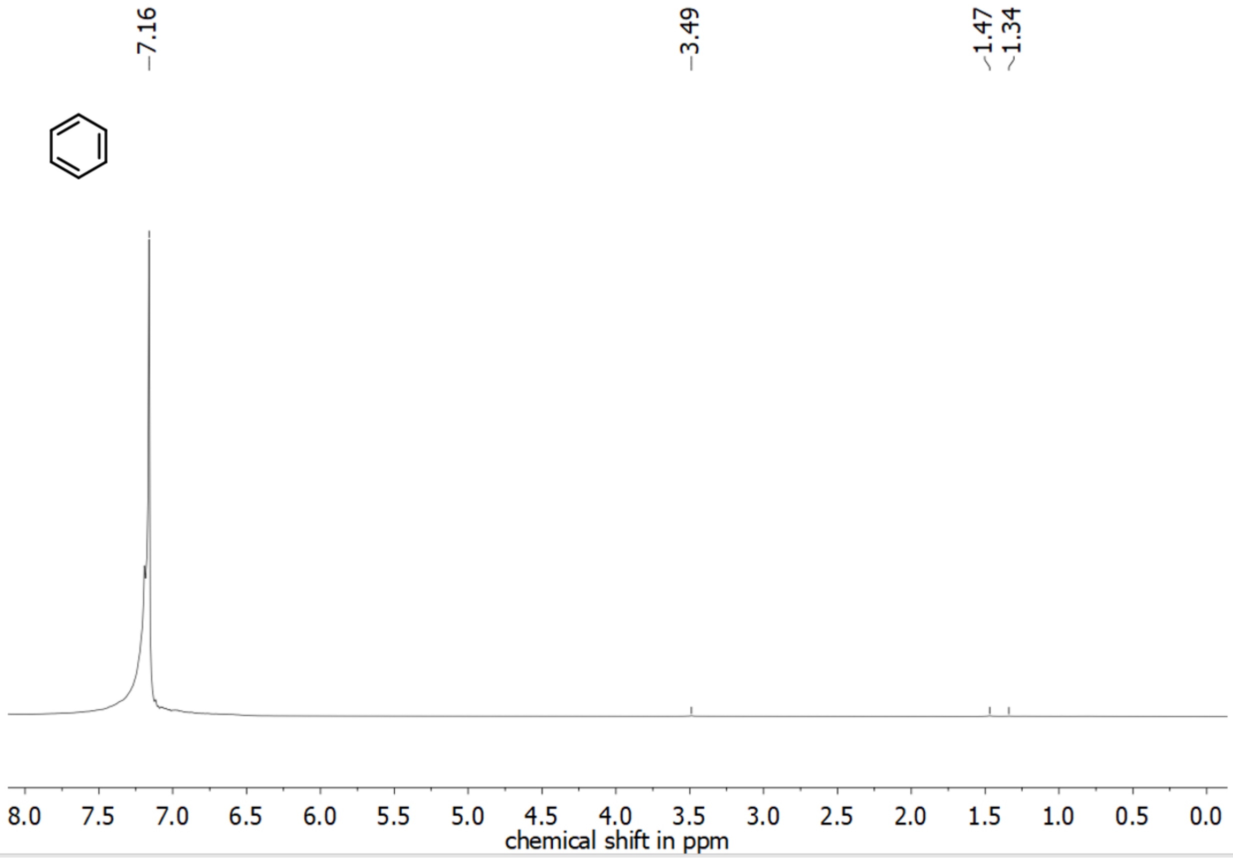


**Figure S83: Attempted hydrogenation of benzene (neat) using 5 mol% $Ba(NH_2)_2$ and 50 bars of $H_2$ at 150°C (24 h).**

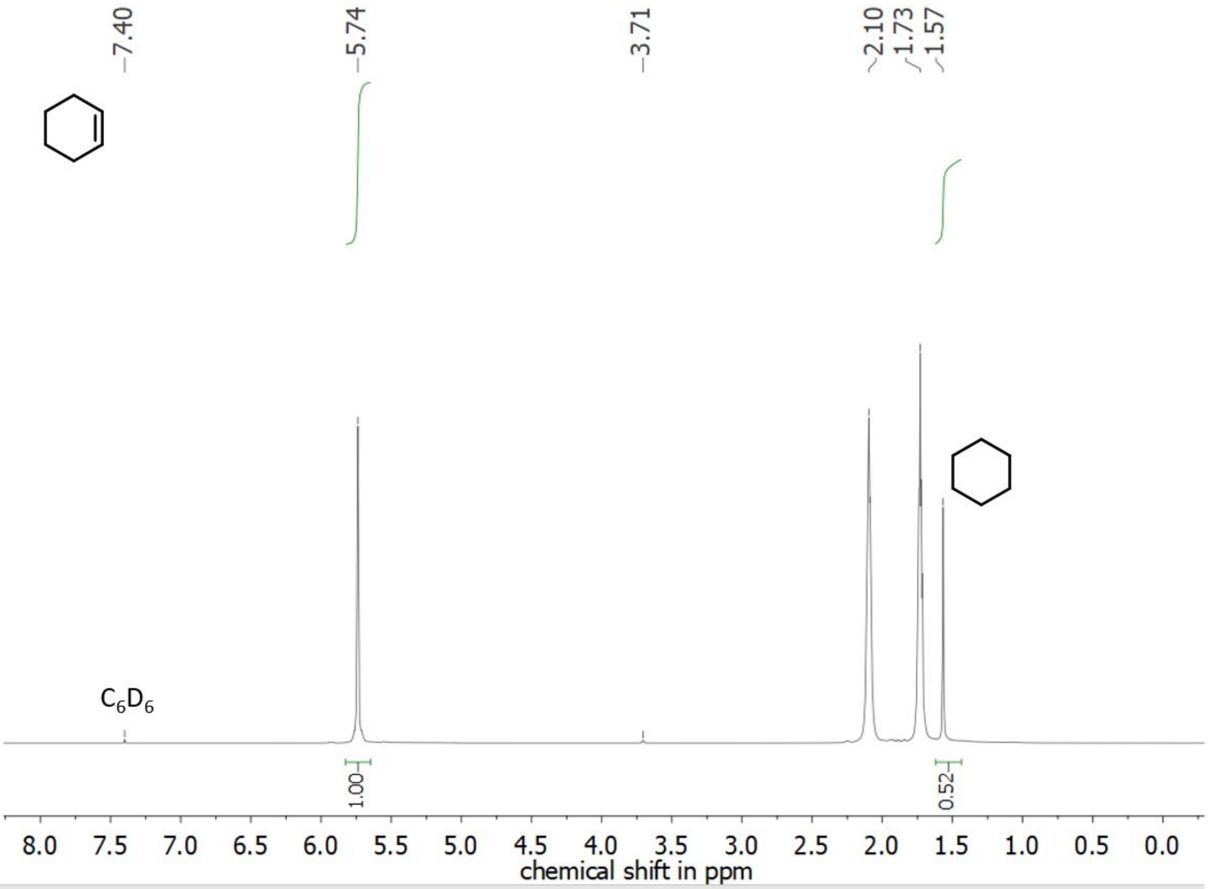


**Figure S84: Hydrogenation of cyclohexene (neat) using 5 mol% Ba(0) and 12 bars of $H_2$ at 120°C (2 h).**

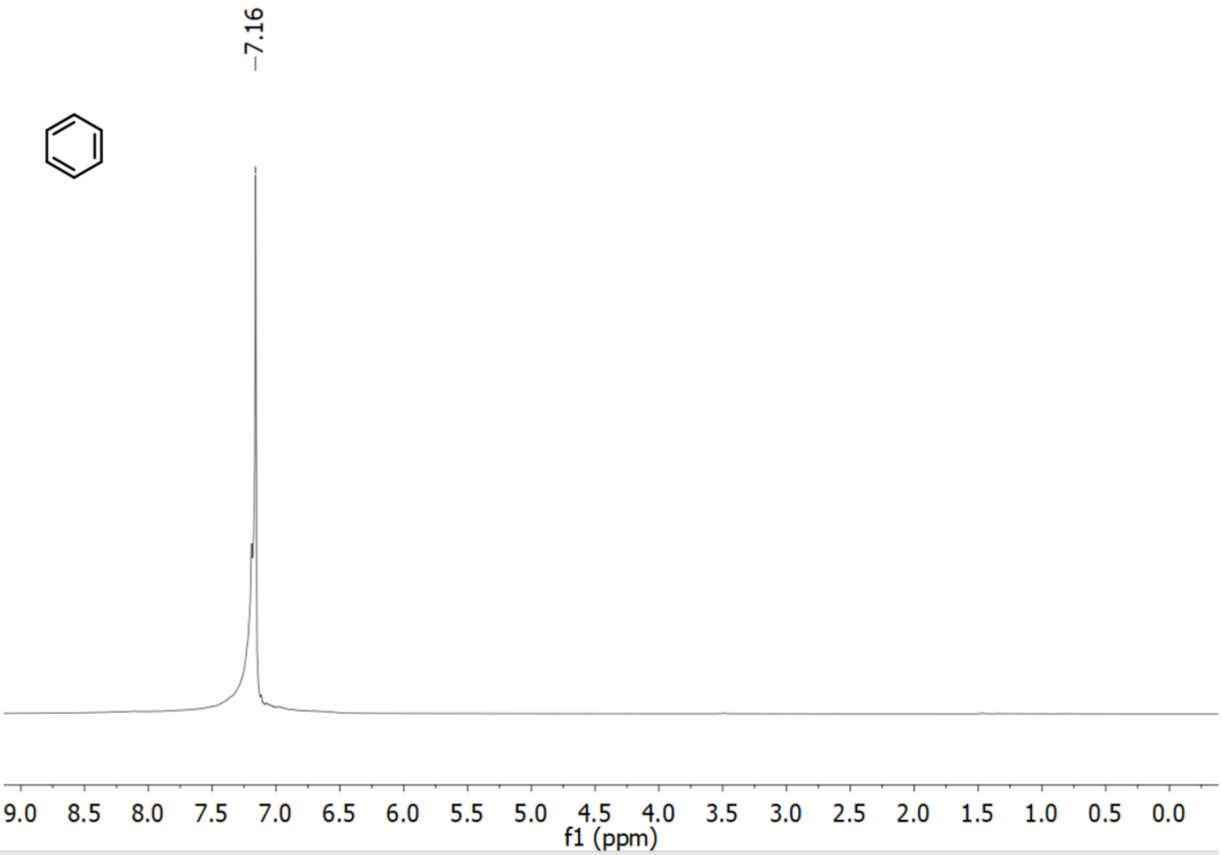


**Figure S85: Attempted hydrogenation of benzene (neat) using 5 mol% Ba(0) and 50 bars of $H_2$ at 150°C (24 h).**

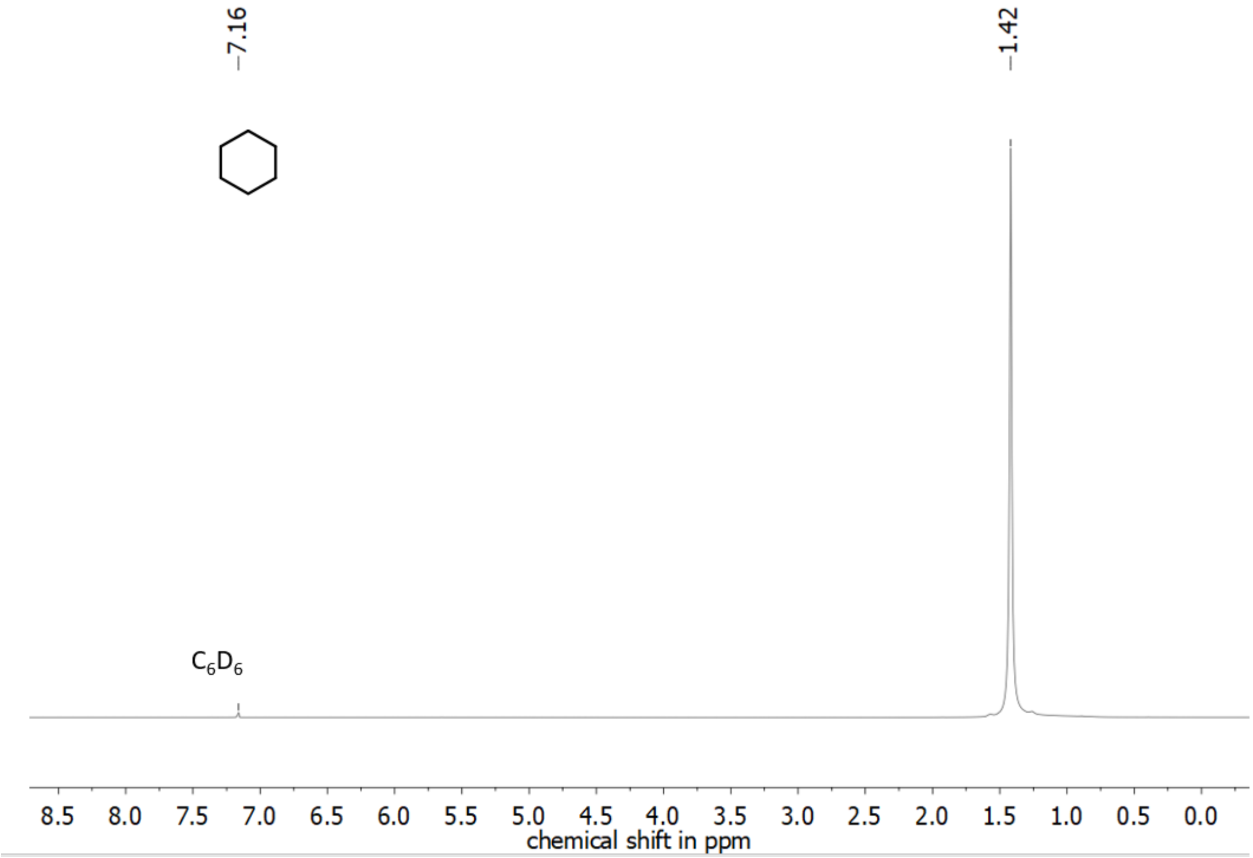


**Figure S86: Hydrogenation of cyclohexene (neat) using 2 mol% Ba(0) (metal vapour synthesis) and 12 bars of $H_2$ at 120°C (2 h).**

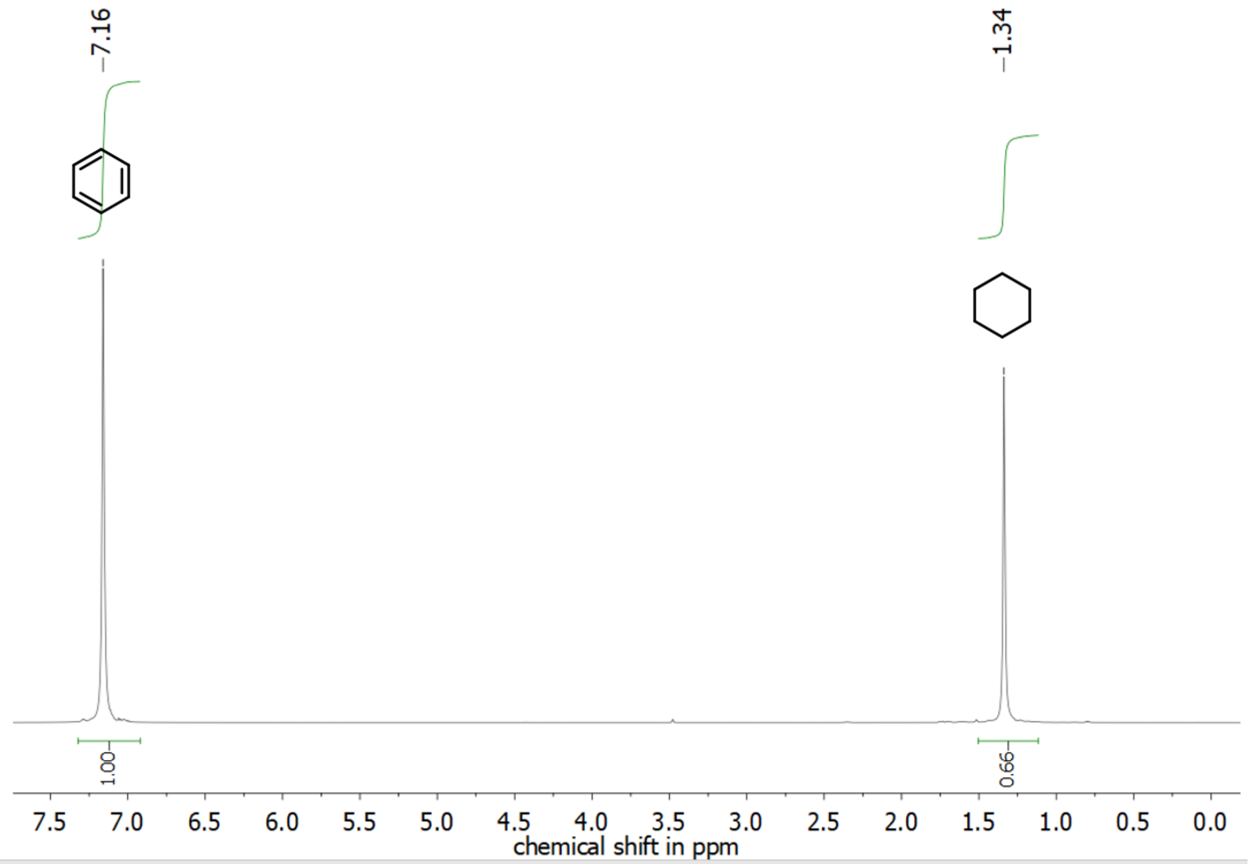


**Figure S87: Hydrogenation of benzene (neat) using 5 mol% Ba(0) (metal vapour synthesis) and 50 bars of $H_2$ at 150°C (24 h).**

# 1.10 DFT calculations

All calculations were carried out using Gaussian 16a.[S13] All structures were fully optimized without the use of symmetry restraints on a B3PW91/def2tzvpp[S14-16] level of theory using dispersion correction according to Grimme with Becke-Johnson dampening (GD3BJ).[S17] Charges were calculated using NBO7.[S18]

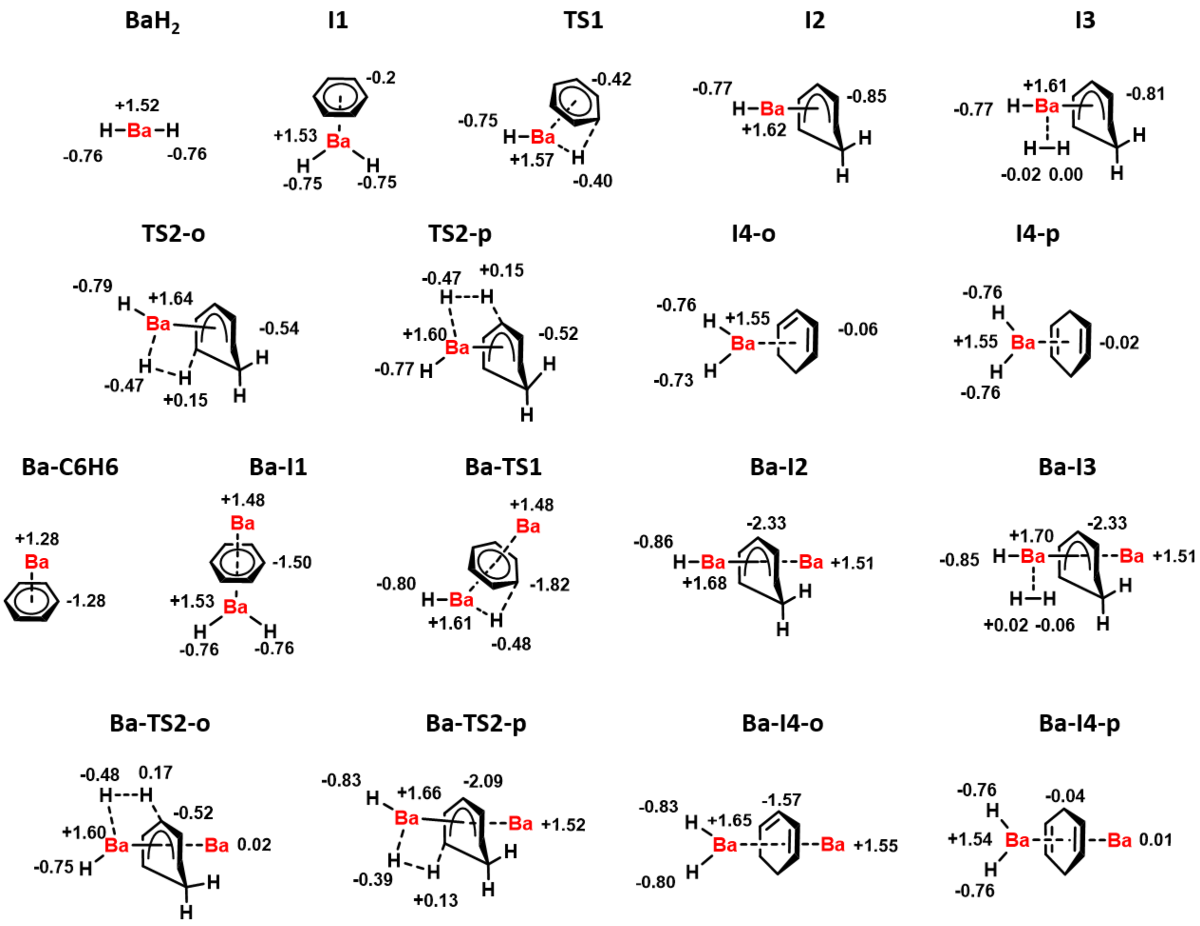


**Figure S88: NPA charges calculated on a B3PW91/def2TZVPP (GD3BJ) level of theory.**

**XYZ-Files:**

```
14
1,4-Cyclohexadiene
H	-2.163855	-0.000005	-0.867404
C	1.489891	0.000003	0.000001
C	-0.663657	1.245964	0.000000
H	-1.197683	2.190753	-0.000001
C	0.663652	1.245966	-0.000001
H	1.197675	2.190757	0.000000
C	-1.489890	-0.000002	0.000000
C	0.663657	-1.245964	0.000000
H	1.197684	-2.190752	-0.000001
C	-0.663653	-1.245966	0.000000
H	-1.197676	-2.190757	-0.000002
H	2.163855	0.000002	-0.867405
H	-2.163853	-0.000004	0.867407
H	2.163853	0.000004	0.867407

14
1,3-Cyclohexadiene
H	-2.031392	1.266079	0.165871
C	1.249781	-0.720384	-0.111545
C	-1.183597	-0.720092	0.251985
H	-2.031387	-1.266088	-0.165870
C	0.108816	-1.414202	-0.063960
H	0.110256	-2.490689	-0.190678
C	-1.183600	0.720087	-0.251985
C	1.249777	0.720389	0.111545
H	2.193373	1.221608	0.292902
C	0.108810	1.414203	0.063960
H	0.110244	2.490691	0.190679
H	2.193380	-1.221597	-0.292901
H	-1.322199	0.728927	-1.342887
H	-1.322195	-0.728934	1.342887

12
Benzene
H	2.438058	-0.407108	0.000943
C	-1.369684	0.228648	0.000062
C	0.486833	-1.300416	-0.000710
H	0.866450	-2.314863	-0.000602
C	-0.882931	-1.071783	0.000440
H	-1.571478	-1.907913	0.000439
C	1.369712	-0.228698	0.000370
```

| | | | |
|---|---|---|---|
| C | -0.486820 | 1.300417 | -0.000431 |
| H | -0.866569 | 2.314816 | 0.000174 |
| C | 0.882900 | 1.071830 | 0.000073 |
| H | -2.438013 | 0.407147 | 0.000267 |
| H | 1.571496 | 1.907933 | -0.000039 |

| | | | |
|---|---|---|---|
| | 3 | | |
| $BaH_2$ | | | |
| Ba | 0.00000 | 0.04306 | 0.00000 |
| H | 1.89535 | -1.20543 | 0.00000 |
| H | -1.89535 | -1.20594 | 0.00000 |

| | | | |
|---|---|---|---|
| | 15 | | |
| I1 | | | |
| H | 1.435756 | 1.932303 | 1.160578 |
| H | -1.903523 | 2.146282 | -1.179850 |
| Ba | 1.275962 | -0.000001 | -0.078531 |
| C | -1.692642 | -1.208465 | 0.742787 |
| C | -1.692618 | 1.208380 | 0.742936 |
| H | -1.604531 | 2.142321 | 1.279939 |
| C | -1.623266 | -0.000086 | 1.431208 |
| H | -1.484568 | -0.000153 | 2.504419 |
| C | -1.844916 | 1.207694 | -0.643265 |
| C | -1.844938 | -1.207607 | -0.643414 |
| H | -1.903567 | -2.146127 | -1.180116 |
| C | -1.928839 | 0.000088 | -1.333351 |
| H | -2.061351 | 0.000156 | -2.408906 |
| H | -1.604576 | -2.142473 | 1.279677 |
| H | 1.435785 | -1.932300 | 1.160581 |

| | | | |
|---|---|---|---|
| | 15 | | |
| TS1 | | | |
| Ba | 1.132632 | -0.018730 | -0.062003 |
| H | -1.716506 | -2.130658 | 1.250971 |
| H | 0.204446 | -1.870505 | 0.979817 |
| C | -1.553199 | 1.267532 | -0.668216 |
| C | -1.617750 | -1.163358 | -0.687696 |
| H | -1.716019 | -2.083221 | -1.250855 |
| C | -1.661041 | 0.049070 | -1.352005 |
| H | -1.791070 | 0.051002 | -2.429106 |
| C | -1.423630 | -1.233909 | 0.728513 |
| C | -1.512127 | 1.221547 | 0.735409 |
| H | -1.496591 | 2.143361 | 1.301787 |
| C | -1.488054 | 0.018543 | 1.414292 |

| | | | |
|---|---|---|---|
| H | -1.615360 | 2.210996 | -1.191307 |
| H | -1.459533 | 0.025685 | 2.496306 |
| H | 1.698070 | 1.745660 | 1.292774 |

15

I2

| | | | |
|---|---|---|---|
| H | -1.669169 | -1.959907 | -1.243695 |
| Ba | 1.157912 | -0.013905 | -0.053697 |
| C | -1.296304 | 1.288532 | 0.851206 |
| C | -1.560603 | 0.198866 | -1.318167 |
| H | -1.586174 | 0.284485 | -2.399442 |
| C | -1.406737 | 1.331749 | -0.556714 |
| H | -1.252281 | 2.282458 | -1.063107 |
| C | -2.033274 | -1.091889 | -0.684780 |
| C | -1.354651 | 0.004151 | 1.461398 |
| H | -1.139242 | -0.079835 | 2.524534 |
| C | -1.538324 | -1.142361 | 0.743175 |
| H | -1.476276 | -2.102912 | 1.238278 |
| H | -1.233742 | 2.192015 | 1.441081 |
| H | -3.136187 | -1.172679 | -0.720583 |
| H | 1.789377 | -2.199262 | 0.253245 |

17

I3

| | | | |
|---|---|---|---|
| H | -1.769982 | -2.258164 | 0.258663 |
| Ba | 1.121287 | -0.167862 | -0.053887 |
| C | -1.247752 | 1.566145 | -0.136797 |
| C | -1.626772 | -0.623130 | -1.151056 |
| H | -1.693746 | -1.226264 | -2.050418 |
| C | -1.408953 | 0.727927 | -1.263068 |
| H | -1.249053 | 1.149019 | -2.253419 |
| C | -2.106083 | -1.221235 | 0.153954 |
| C | -1.335648 | 0.946939 | 1.143809 |
| H | -1.093448 | 1.535124 | 2.026039 |
| C | -1.585683 | -0.384165 | 1.300503 |
| H | -1.546666 | -0.825710 | 2.287956 |
| H | -1.170411 | 2.638956 | -0.240950 |
| H | -3.210430 | -1.278099 | 0.182392 |
| H | 1.945188 | 2.663773 | 0.592972 |
| H | 1.220879 | 2.655857 | 0.384046 |
| H | 1.640948 | -1.729091 | 1.546299 |

17

TS2-o

| | | | |
|---|---|---|---|
| H | 1.250308 | -1.902934 | -1.099912 |
| Ba | -1.186079 | -0.024113 | -0.099608 |
| C | 1.352142 | 1.369082 | 0.765538 |
| C | 1.464506 | -1.100611 | 0.875904 |
| H | 1.957725 | -1.830499 | 1.515898 |
| C | 1.383283 | 0.210655 | 1.483690 |
| H | 1.268399 | 0.257424 | 2.562181 |
| C | 1.908988 | -1.170549 | -0.590655 |
| C | 1.593250 | 1.317029 | -0.662316 |
| H | 1.602602 | 2.245146 | -1.223354 |
| C | 1.842552 | 0.154157 | -1.295097 |
| H | 2.080482 | 0.165357 | -2.353633 |
| H | 1.234940 | 2.325075 | 1.257200 |
| H | 2.916873 | -1.586959 | -0.706234 |
| H | -0.585403 | -2.297658 | 0.336437 |
| H | 0.337137 | -1.728150 | 0.805033 |
| H | -2.910937 | 1.024951 | 1.022080 |

17
TS2-p

| | | | |
|---|---|---|---|
| Ba | 1.192694 | -0.092578 | 0.019557 |
| C | -1.604426 | -0.525190 | 1.292297 |
| C | -1.222655 | 1.541986 | -0.025425 |
| H | -1.656610 | 2.541629 | -0.008400 |
| C | -1.375885 | 0.804938 | 1.225636 |
| H | -1.240422 | 1.354876 | 2.153408 |
| C | -1.557994 | 0.744459 | -1.220316 |
| C | -1.935776 | -1.359322 | 0.082772 |
| H | -2.956214 | -1.759729 | 0.177555 |
| C | -1.813774 | -0.569581 | -1.191195 |
| H | -1.968994 | -1.105796 | -2.119536 |
| H | -1.666392 | -1.004665 | 2.263548 |
| H | 1.105986 | 2.200380 | -0.653386 |
| H | 0.021137 | 1.986993 | -0.285905 |
| H | -1.502138 | 1.245902 | -2.181133 |
| H | -1.304870 | -2.256692 | 0.021590 |
| H | 1.440718 | -1.842246 | -1.445537 |

17
I4-o

| | | | |
|---|---|---|---|
| H | 2.028078 | -1.949619 | -1.115593 |
| Ba | -1.318145 | -0.039968 | -0.103412 |
| C | 1.441403 | 1.349336 | 0.764004 |
| C | 1.647705 | -1.121314 | 0.834767 |

| | | | |
|---|---|---|---|
| H | 2.202378 | -1.789259 | 1.495419 |
| C | 1.476347 | 0.212640 | 1.481765 |
| H | 1.314952 | 0.246860 | 2.552855 |
| C | 2.311853 | -1.061740 | -0.545454 |
| C | 1.603834 | 1.312701 | -0.685683 |
| H | 1.444857 | 2.224473 | -1.251400 |
| C | 1.968194 | 0.182588 | -1.311159 |
| H | 2.114188 | 0.179371 | -2.385844 |
| H | 1.273648 | 2.301371 | 1.251549 |
| H | 3.405417 | -1.099058 | -0.454391 |
| H | -1.022088 | -2.329954 | -0.028767 |
| H | 0.663521 | -1.649202 | 0.732493 |
| H | -2.304876 | 0.857979 | 1.765292 |

| | | | |
|---|---|---|---|
| | 17 | | |
| I4-p | | | |
| H | 0.894011 | -2.211202 | 0.020612 |
| Ba | -1.289410 | -0.000013 | -0.052586 |
| C | 1.731150 | 1.493115 | 0.015509 |
| C | 1.652169 | -0.666297 | 1.261550 |
| H | 1.563776 | -1.206003 | 2.196565 |
| C | 1.652126 | 0.666300 | 1.261564 |
| H | 1.563697 | 1.205980 | 2.196591 |
| C | 1.731254 | -1.493079 | 0.015477 |
| C | 1.724200 | 0.668144 | -1.233137 |
| H | 1.741211 | 1.200693 | -2.179172 |
| C | 1.724246 | -0.668082 | -1.233152 |
| H | 1.741290 | -1.200609 | -2.179198 |
| H | 0.893854 | 2.211178 | 0.020663 |
| H | 2.626634 | -2.127077 | 0.033666 |
| H | -1.367381 | -1.973452 | 1.137262 |
| H | 2.626482 | 2.127179 | 0.033711 |
| H | -1.367482 | 1.973424 | 1.137256 |

| | | | |
|---|---|---|---|
| | 16 | | |
| Ba-I1 | | | |
| H | -2.917774 | -1.956943 | -1.207495 |
| H | -0.119734 | -1.225617 | -2.087536 |
| Ba | -2.632763 | 0.000000 | 0.031538 |
| C | 0.152744 | 0.688197 | 1.281909 |
| C | 0.357018 | -1.445442 | 0.051348 |
| H | 0.127014 | -2.501090 | 0.048804 |
| C | 0.152744 | -0.688196 | 1.281909 |
| H | 0.025311 | -1.221053 | 2.218577 |

| | | | |
|---|---|---|---|
| C | 0.098614 | -0.685831 | -1.174813 |
| C | 0.357028 | 1.445445 | 0.051351 |
| H | 0.127022 | 2.501093 | 0.048812 |
| C | 0.098617 | 0.685839 | -1.174812 |
| H | -0.119729 | 1.225629 | -2.087532 |
| H | 0.025311 | 1.221053 | 2.218579 |
| H | -2.917727 | 1.956947 | -1.207494 |
| Ba | 2.605436 | -0.000002 | -0.028789 |

16
Ba-TS1

| | | | |
|---|---|---|---|
| Ba | -2.512625 | -0.011852 | 0.018183 |
| H | 0.195150 | 2.231210 | -1.166738 |
| H | -1.745790 | 2.081125 | -0.638672 |
| C | 0.081071 | -1.186245 | 0.804534 |
| C | 0.083504 | 0.097257 | -1.328076 |
| H | -0.039373 | 0.099649 | -2.403820 |
| C | 0.124894 | -1.204544 | -0.622300 |
| H | -0.081895 | -2.116376 | -1.164240 |
| C | 0.035291 | 1.313513 | -0.621243 |
| C | 0.164612 | 0.015320 | 1.503886 |
| H | 0.159859 | 0.000059 | 2.587934 |
| C | 0.348396 | 1.295491 | 0.804086 |
| H | 0.017535 | -2.121886 | 1.350084 |
| H | 0.200036 | 2.208798 | 1.363806 |
| H | -3.795934 | -1.378164 | -1.428635 |
| Ba | 2.513764 | -0.041526 | -0.049345 |

16
Ba-I2

| | | | |
|---|---|---|---|
| H | 0.794749 | 2.379287 | 0.000022 |
| H | -0.900061 | 2.152865 | 0.000019 |
| Ba | -2.407214 | 0.001663 | -0.000001 |
| C | 0.046314 | -1.426440 | -0.000011 |
| C | 0.204210 | 0.729099 | -1.266938 |
| H | 0.109328 | 1.247566 | -2.213432 |
| C | 0.052806 | -0.717771 | -1.232254 |
| H | 0.025505 | -1.268255 | -2.164967 |
| C | 0.051515 | 1.554288 | 0.000014 |
| C | 0.052806 | -0.717793 | 1.232244 |
| H | 0.025503 | -1.268295 | 2.164947 |
| C | 0.204211 | 0.729074 | 1.266953 |
| H | 0.109327 | 1.247525 | 2.213455 |
| H | 0.000342 | -2.508910 | -0.000021 |

| | | | |
|---|---|---|---|
| H | -4.661977 | -0.827175 | 0.000008 |
| Ba | 2.421967 | -0.038401 | -0.000001 |

18

Ba-I3

| | | | |
|---|---|---|---|
| H | 0.801018 | -2.201909 | 0.000039 |
| Ba | 2.358747 | -0.096681 | -0.000001 |
| C | -0.057611 | 1.404250 | -0.000009 |
| C | -0.261952 | -0.746301 | -1.267988 |
| H | -0.180268 | -1.267128 | -2.214306 |
| C | -0.082617 | 0.697009 | -1.232624 |
| H | -0.043723 | 1.247212 | -2.165055 |
| C | -0.131398 | -1.575229 | 0.000025 |
| C | -0.082624 | 0.697035 | 1.232620 |
| H | -0.043733 | 1.247260 | 2.165039 |
| C | -0.261968 | -0.746273 | 1.268017 |
| H | -0.180281 | -1.267079 | 2.214346 |
| H | 0.009762 | 2.485439 | -0.000021 |
| H | -0.897503 | -2.379305 | 0.000030 |
| H | 3.768916 | 2.300674 | 0.000011 |
| H | 3.151463 | 2.746866 | 0.000049 |
| H | 4.727849 | 0.338448 | -0.000092 |
| Ba | -2.463113 | 0.067513 | -0.000004 |

18

Ba-TS2-o

| | | | |
|---|---|---|---|
| H | -0.499919 | 2.075382 | 1.145804 |
| Ba | -2.484583 | -0.039915 | 0.009509 |
| C | 0.076299 | -1.283655 | -0.532909 |
| C | 0.058267 | 1.242623 | -0.758251 |
| H | 0.593116 | 2.011501 | -1.329770 |
| C | 0.142443 | -0.121925 | -1.360888 |
| H | -0.000965 | -0.217949 | -2.430371 |
| C | 0.276114 | 1.391082 | 0.753604 |
| C | 0.152488 | -1.171565 | 0.866483 |
| H | 0.120503 | -2.073051 | 1.468006 |
| C | 0.311704 | 0.091616 | 1.528424 |
| H | 0.216763 | 0.145066 | 2.605664 |
| H | 0.001576 | -2.264636 | -0.987467 |
| H | 1.205366 | 1.993656 | 0.935868 |
| H | -2.105694 | 2.356597 | -0.639419 |
| H | -1.185450 | 1.900625 | -0.829905 |
| H | -4.331117 | -1.401680 | -0.560648 |
| Ba | 2.482474 | -0.056774 | -0.051590 |

18
Ba-TS2-p

| | | | |
|---|---|---|---|
| Ba | 3.040582 | -0.107043 | -0.100112 |
| C | 0.260353 | 0.627857 | -1.206277 |
| C | 0.306934 | -1.182939 | 0.494329 |
| H | -0.277531 | -2.093168 | 0.657204 |
| C | 0.223397 | -0.686956 | -0.881016 |
| H | 0.163187 | -1.419842 | -1.681921 |
| C | 0.064066 | -0.125171 | 1.503261 |
| C | 0.223512 | 1.724832 | -0.177370 |
| H | -0.598209 | 2.421109 | -0.416547 |
| C | 0.049478 | 1.182299 | 1.214624 |
| H | -0.085630 | 1.906350 | 2.009810 |
| H | 0.202131 | 0.916729 | -2.250595 |
| H | 2.549586 | -1.978341 | 1.268118 |
| H | 1.451303 | -1.700866 | 0.854611 |
| H | -0.051087 | -0.440366 | 2.535141 |
| H | 1.123025 | 2.361384 | -0.205024 |
| H | 3.520155 | 1.906489 | 0.850658 |
| Ba | -3.304213 | -0.091511 | -0.066080 |

18
Ba-I4-o

| | | | |
|---|---|---|---|
| H | -0.595460 | 2.002610 | 1.077069 |
| Ba | -2.601357 | -0.051751 | -0.000002 |
| C | 0.128068 | -1.170913 | -0.699441 |
| C | 0.272469 | 1.392571 | -0.779625 |
| H | 1.139245 | 2.011031 | -1.112298 |
| C | 0.293234 | 0.034395 | -1.444905 |
| H | 0.174039 | 0.011882 | -2.521116 |
| C | 0.271884 | 1.392319 | 0.780208 |
| C | 0.128110 | -1.171153 | 0.699276 |
| H | 0.042325 | -2.112811 | 1.229893 |
| C | 0.293522 | 0.033905 | 1.445096 |
| H | 0.174416 | 0.011036 | 2.521312 |
| H | 0.042169 | -2.112379 | -1.230382 |
| H | 1.137752 | 2.011433 | 1.113755 |
| H | -2.569682 | 2.339556 | -0.000791 |
| H | -0.594039 | 2.003712 | -1.077059 |
| H | -4.564989 | -1.370468 | -0.000992 |
| Ba | 2.552973 | -0.088648 | -0.000053 |

18

Ba-I4-p

| | | | |
|---|---|---|---|
| H | 1.094995 | -2.154204 | -0.023981 |
| Ba | 3.348302 | -0.000003 | 0.011888 |
| C | 0.213117 | 1.495405 | 0.055687 |
| C | 0.130399 | -0.667310 | -1.189382 |
| H | 0.100805 | -1.205190 | -2.129423 |
| C | 0.130399 | 0.667305 | -1.189391 |
| H | 0.100805 | 1.205171 | -2.129440 |
| C | 0.213118 | -1.495392 | 0.055708 |
| C | 0.252748 | 0.669112 | 1.302921 |
| H | 0.254348 | 1.200626 | 2.249333 |
| C | 0.252746 | -0.669081 | 1.302930 |
| H | 0.254345 | -1.200580 | 2.249350 |
| H | 1.094988 | 2.154223 | -0.024019 |
| H | -0.634294 | -2.196144 | 0.107177 |
| H | 3.220540 | -1.954702 | -1.185626 |
| H | -0.634301 | 2.196154 | 0.107148 |
| H | 3.220585 | 1.954699 | -1.185621 |
| Ba | -3.620230 | -0.000002 | -0.013062 |

13

Ba-$C_6H_6$

| | | | |
|---|---|---|---|
| H | 1.661025 | -1.218602 | 2.142643 |
| C | 1.469543 | 0.683856 | -1.218531 |
| C | 1.213438 | -1.435325 | -0.000018 |
| H | 1.397138 | -2.500436 | -0.000032 |
| C | 1.469542 | -0.683824 | -1.218548 |
| H | 1.661028 | -1.218546 | -2.142674 |
| C | 1.469540 | -0.683856 | 1.218531 |
| C | 1.213438 | 1.435326 | 0.000019 |
| H | 1.397138 | 2.500436 | 0.000033 |
| C | 1.469541 | 0.683824 | 1.218549 |
| H | 1.661025 | 1.218547 | 2.142675 |
| H | 1.661029 | 1.218602 | -2.142642 |
| Ba | -1.058368 | 0.000000 | 0.000000 |

## 2. References


[S1] J. Barluenga, A. Jimenez-Aquino, F. Aznar, C. Valdes, *J. Am. Chem. Soc.* **2009**, *131*, 4031–4041

[S2] R. Wang, M. Ma, X. Gong, G. B. Panetti, X. Fan, P.J. Walsh, *Org. Letters* **2018**, *20*, 2433-2436.

[S3] (a) P. L. Timms, *J. Chem. Educ.* **1972**, *49*, 782-784; (b) U. Zenneck, Chemie in unserer Zeit **1993**, *27*, 208-219.

[S4] J. Martin, C. Knüpfer, J. Eyselein, C. Färber, S. Grams, J. Langer, K. Thum, M. Wiesinger, S. Harder, *Angew. Chem. Int. Ed.* **2020**, *132*, 9187-9197.

[S5] M. Yalpani, R. Köster, *Chem. Ber.* **1990**, *123*, 719-724.

[S6] A. S. Pedersen, *Diffusion and Defect Data* 1996, *49-50*, 35-70.

[S7] Rigaku Oxford Diffraction, **2019**, CrysAlisPro Software system, version 1.171.40.53, Rigaku Corporation, Oxford, UK.

[S8] O. V. Dolomanov, L. J. Bourhis, R.J. Gildea, J. A. K. Howard and H. Puschmann, *J. Appl. Cryst.,* **2009**, 42, 339–341.

[S9] G. M. Sheldrick, *Acta Cryst. A,* **2015**, 71, 3–8.

[S10] G. M. Sheldrick, *Acta Cryst. C,* **2015**, 71, 3–8.

[S11] A. Thorn, B. Dittrich and G. M. Sheldrick, *Acta Cryst. A,* **2012**, 68, 448–451.

[S12] Q. R. Wang, Y. Q. Guan, W. B. Gao, J. P. Guo, P. Chen, *ChemPhysChem.* **2019**, *20*, 1376 –1381.

[S13] M. J. Frisch, G. W. Trucks, H. B. Schlegel, G. E. Scuseria, M. A. Robb, J. R. Cheeseman, G. Scalmani, V. Barone, G. A. Petersson, H. Nakatsuji, X. Li, M. Caricato, A. V. Marenich, J. Bloino, B. G. Janesko, R. Gomperts, B. Mennucci, H. P. Hratchian, J. V. Ortiz, A. F. Izmaylov, J. L. Sonnenberg, D. Williams-Young, F. Ding, F. Lipparini, F. Egidi, J. Goings, B. Peng, A.

Petrone, T. Henderson, D. Ranasinghe, V. G. Zakrzewski, J. Gao, N. Rega, G. Zheng, W. Liang, M. Hada, M. Ehara, K. Toyota, R. Fukuda, J. Hasegawa, M. Ishida, T. Nakajima, Y. Honda, O. Kitao, H. Nakai, T. Vreven, K. Throssell, J. A. Montgomery, J. E. Peralta, F. Ogliaro, M. J. Bearpark, J. J. Heyd, E. N. Brothers, K. N. Kudin, V. N. Staroverov, T. A. Keith, R. Kobayashi, J. Normand, K. Raghavachari, A. P. Rendell, J. C. Burant, S. S. Iyengar, J. Tomasi, M. Cossi, J. M. Millam, M. Klene, C. Adamo, R. Cammi, J. W. Ochterski, R. L. Martin, K. Morokuma, O. Farkas, J. B. Foresman, D. J. Fox, *Gaussian 16 Rev. A.03*, Wallingford CT, **2016**.

[S14] A. D. Becke, *J. Chem. Phys.* **1993**, *98*, 1372–1377.

[S15] J. P. Perdew, *Electronic Structure of Solids*, Akademie Verlang, Berlin, **1991**.

[S16] F. Weigend, R. Ahlrichs, *Phys. Chem. Chem. Phys.* **2005**, 3297–3305.

[S17] S. Grimme, J. Antony, S. Ehrlich, H. Krieg, *J. Chem. Phys.* **2010**, *132*, 154104.

[S18] A. E. Reed, R. B. Weinstock, F. Weinhold, *J. Chem. Phys.* **1985**, *83*, 735–746.
